# Triple-negative breast carcinomas of low malignant potential: review on diagnostic criteria and differential diagnoses

**DOI:** 10.1007/s00428-021-03174-7

**Published:** 2021-08-30

**Authors:** L. Cima, H. Kaya, C. Marchiò, R. Nishimura, H. Y. Wen, V. P. Fabbri, M. P. Foschini

**Affiliations:** 1 Pathology Unit, Department of Clinical Services, Santa Chiara Hospital, Trento, Italy; 2 Department of Pathology, Pendik Research Training Hospital, Marmara University, Muhsin Yazicioglu Cad. No: 10, Pendik, 34899 Istanbul, Turkey; 3 Division of Pathology, Candiolo Cancer Institute FPO-IRCCS, Candiolo, Italy; 4 Department of Medical Sciences, University of Turin, Turin, Italy; 5 Department of Pathology, Nagoya Medical Center, 4-1-1 Sannomaru, Naka-ku, Nagoya, Aichi 460-0001 Japan; 6 Department of Pathology, Memorial Sloan Kettering Cancer Center, New York, NY USA; 7 Department of Biomedical and Neuromotor Sciences, University of Bologna, 40139 Bologna, Italy; 8 Section of Anatomic Pathology, Bellaria Hospital, Via Altura 3, 40139 Bologna, Italy

**Keywords:** Triple-negative breast carcinoma, Adenoid cystic carcinoma, Adenomyoepithelioma, Acinic cell carcinoma, Mucoepidermoid carcinoma, Tall cell carcinoma with reverse polarity, Secretory carcinoma

## Abstract

Triple-negative breast carcinomas constitute a wide spectrum of lesions, mostly being highly aggressive. Nevertheless, some special histologic subtypes can have low malignant potential. The purpose of the present paper is to review diagnostic criteria and prognostic parameters of breast neoplasms of special histotypes. Specifically, adenoid cystic carcinoma, adenomyoepithelioma, acinic cell carcinoma, mucoepidermoid carcinoma, tall cell carcinoma with reverse polarity, and secretory carcinoma will be discussed. For each tumour, definition and morphological and molecular features, together with prognostic parameters, will be presented. Paradigmatic cases will be illustrated.

**Supplementary Information:**

The online version contains supplementary material available at 10.1007/s00428-021-03174-7.

## Introduction

Triple-negative breast carcinomas (TNBCs) are invasive breast carcinomas lacking oestrogen receptor (ER) or progesterone receptor (PR) expression and HER2 amplification. The term TNBC has acquired an ominous meaning as in most of the cases it refers to poorly differentiated breast carcinomas, with highly aggressive behaviour. Normal breast glands are composed of different cells, not all expressing hormone receptors but all of them having the ability to transform into malignant tumours of varying clinical behaviour. Therefore, even if rarely, TNBC of low malignant potential can occur [1]. Despite the attention toward these histologic subtypes has grown in the last decade, knowledge on TNBC of low malignant potential is still limited due to their rarity. Lack of knowledge can result in inappropriate treatment.

The purpose of the present paper is to review the recent literature on TNBC of low malignant potential, focusing on diagnostic criteria and prognostic features. The main features of each tumour are summarized in Table 1.

**Table 1:** Summary of the low malignant potential triple-negative breast carcinomas

Tumour type	Histology	Immunohistochemistry	Molecular profile	Differential diagnosis	Prognosis
Adenoid cystic carcinoma, classical variant.	Epithelial and myoepithelial cells Architecture: tubular, cribriform, solid.	CD117 and MYB diffuse positivity	*MYB-NFIB* fusion gene *MYBL1* rearrangements *MYB* amplification	Microglandular adenosis Adenomyoepithelioma Adenoid cystic carcinoma solid-basaloid and with high-grade transformation Cribriform carcinoma Neuroendocrine carcinoma	Good for the classical variant More aggressive for the solid-basaloid and the high-grade transformation variants
Adenomyoepithelioma	Glands lined by epithelial cells and by a prominent layer of myoepithelial cells.	Epithelial and myoepithelial markers Antibody for Q61R mutant NRAS	PI3K pathway genes mostly in ER +ve HRAS Q61 and PI3K pathway genes mostly in ER-ve	Pleomorphic adenoma; adenoid cystic carcinoma Low-grade adenosquamous carcinomas Microglandular adenosis Intraductal papilloma with florid myoepithelial hyperplasia	Good Local recurrences possible Distant metastases possible but rare More aggressive prognosis in cases with malignant transformation
Mucoepidermoid carcinoma	Glands lined by mucoid, epidermoid, and intermediate cells	CK 14, CK5/6, p63; anti-mitochondrial antigen staining the basaloid and epidermoid cells CK7 and EMA staining the mucoid cells	MAML2 rearrangements	Adenosquamous carcinoma Metaplastic carcinoma	Good for low and intermediate grade Aggressive for high-grade cases
Acinic cell carcinoma	Cells with evidence of serous acinar differentiation Presence of intracytoplasmic granules Neoplastic cells arranged in small glands and/or solid nests	Low-weight CK S-100 EMA Lysozyme Alpha-1-antichimotrypsin Amylase	No specific mutational profile Similar to other triple-negative breast carcinoma with TP53 mutation, PIK3CA hotspot mutations.	Microglandular adenosis Invasive carcinoma of no special type	Good Recurrences and metastases are possible even if rare
Tall cell carcinoma with reverse polarity	Cells oval with eosinophilic and finely granular cytoplasm; nuclear polarity toward the periphery of the neoplastic solid nests or papillae. Architecture: solid and solid-papillary patterns	CK high- and low-weight +ve GATA 3 +ve Mammaglobin: +ve Anti-mitochondrial antigen: +ve TTF1 and thyroglobulin: -ve Neuroendocrine markers: -ve	Dehydrogenase 2 (IDH2) and phosphatidylinositol 3-kinase catalytic alpha (PIK3CA) hotspot mutations	Metastases from thyroid carcinoma Neuroendocrine carcinoma Intraductal papilloma	Good Recurrences and axillary node metastases possible but exceedingly rare
Secretory carcinoma	Polygonal cells, with granular or vacuolated cytoplasm. Mild to moderate nuclear pleomorphism Architecture: usually microcystic growth	CK high- and low-weight +ve S-100: +ve	ETV6-NTRK3 fusion gene TERT promotor mutations and loss of CDKN2A/B in aggressive cases	Carcinoma with apocrine differentiation Acinic cell carcinoma Tall cell carcinoma with reversed polarity	Good especially in young patients

*F* female, *ER* oestrogen, *+ve* positive, *-ve* negative, *CK* cytokeratin, *EMA* epithelial membrane antigen

## Tumours with epithelial and myoepithelial differentiation

### Adenoid cystic carcinoma (AdCC)

AdCC is an invasive carcinoma composed of epithelial and myoepithelial cells, arranged in tubular, cribriform and solid structures [2]. It is morphologically similar to the salivary gland counterpart, but of low malignant potential when affecting the breast.

AdCC affects mainly adult and elderly women, but on rare occasions can affect young and male patients (review in ref. [3]). It presents more frequently as a single, palpable nodule located in the retroareolar region.

The increased use of screening mammography has led to the detection of small forms arising in all breast quadrants. On very rare occasions AdCC can be multifocal [4].

AdCC presents as a mass with regular margins, which can be misdiagnosed as fibroadenoma on imaging [5].

Pre-operative diagnosis can be challenging, if the possibility of a salivary gland-like carcinoma is not kept in mind. Fine-needle aspiration cytology can be difficult to interpretate, characterized by basaloid cells surrounding hyaline globules with a microcystic pattern. The differential diagnosis between AdCC and collagenous spherulosis can be very difficult and requires histology [6, 7]. Pre-operative diagnosis based on core needle biopsy (CNB) can be made based on the same criteria applied on surgical specimens with the aid of immunohistochemistry.

On histology, breast AdCC can exhibit three different subtypes, with prognostic impact [2].

#### AdCC classic variant (C-AdCC) (Fig. 1).

Most frequently shows cribriform architecture with glandular spaces surrounded by epithelial cells and pseudo-glandular spaces lined by myoepithelial cells. Peripheral areas with tubular architecture can be seen. Necrosis is absent and mitotic figures are rare. Perineural invasion is rare. On immunohistochemistry, markers of basement membrane such as collagen IV and laminin highlight the content of the pseudovascular spaces. Epithelial and myoepithelial markers are useful to show the biphasic nature of the tumour. CD117 is usually diffusely positive especially staining the epithelial cells. Strong and diffuse MYB expression by immunohistochemistry can be used to support a diagnosis of AdCC, reported in 65–100% of the cases [8–10].

**Fig. 1:**
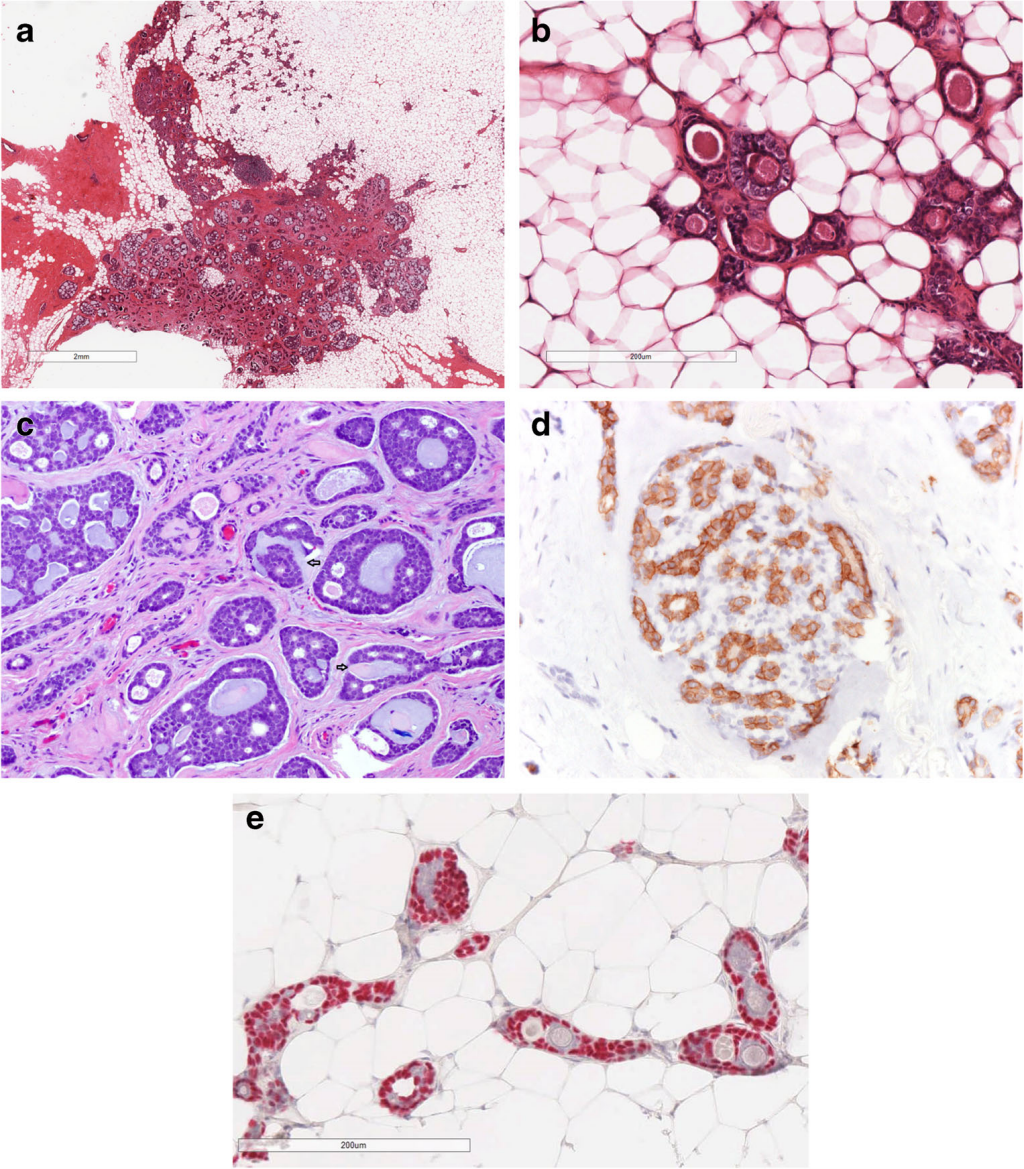
C-AdCC; **a** the central component of the tumor shows the typical cribrifom architecture, while in the peripheral part the tubular achitecture predominates. **b** AdCC with tubular architecture is composed of glands lined by epithelial and myoepithelial cells. The glands contain mucous or basal membrane. **c** C- AdCC with cribriform architecture is composed of neoplastic nests showing glandular structures containing mucous and of pseudoglandular spaced containing basal membrane invaginations (arrows); **d** The epithelial cells are CD117 positive; **e** p63 highlights the presence of myoepithelial cells in the tubular areas

#### AdCC solid-basaloid variant (SB-AdCC) (Fig. 2*)*.

In addition to the classic AdCC features, it is composed of solid areas, with nuclear atypia and high mitotic count [11]. Necrosis and perineural invasion are frequently detected [12].

**Fig. 2:**
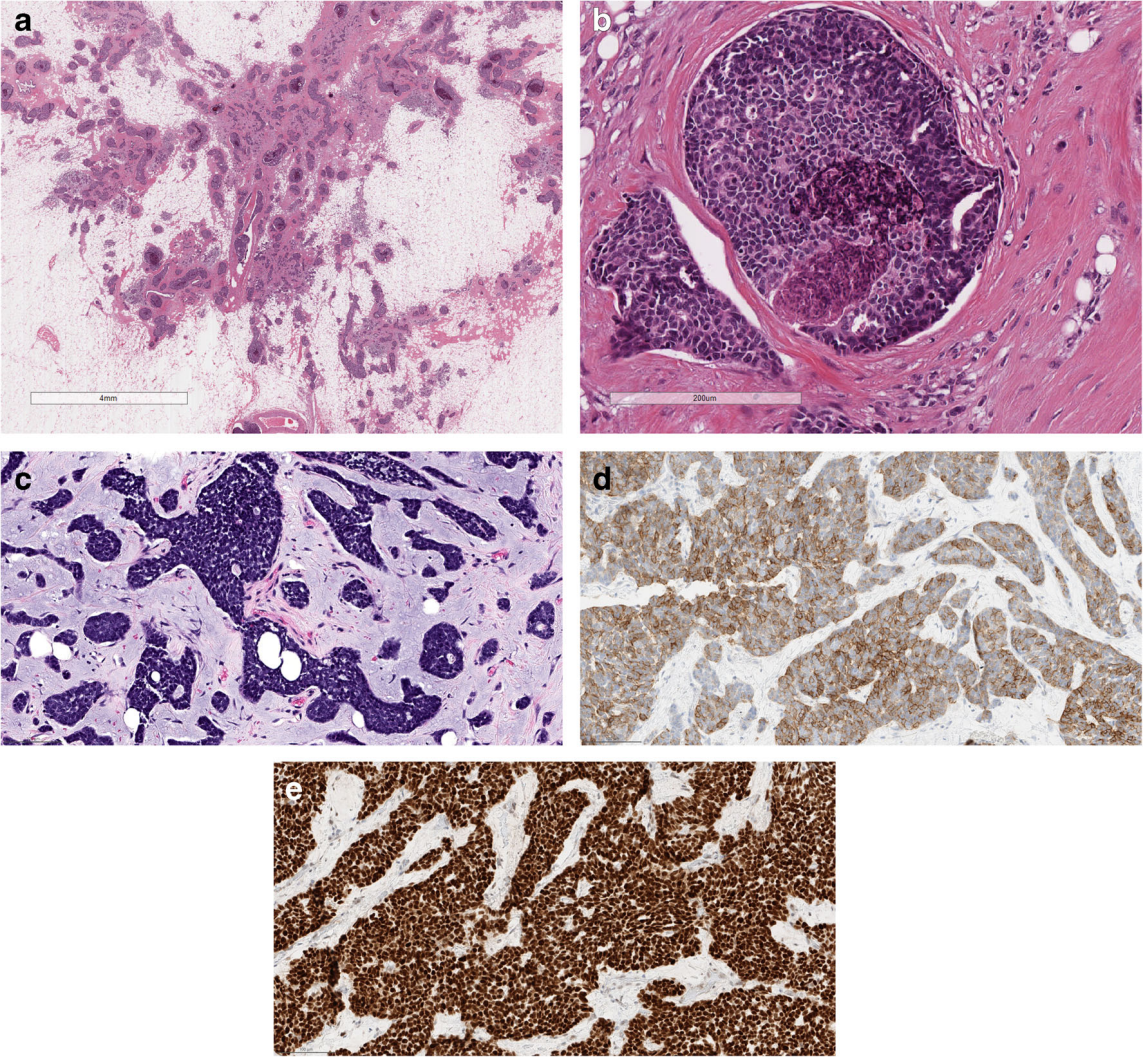
SB-AdCC; **a** at low power view it is composed of multiple solid neoplastic nests; **b** at higher power the neoplastic nests are composed of markedly atipical cells, necrosis is often present; **c** SB AdCC is composed of cells with markedly atipica nucleus and scanty cytoplasm that must be differentiated from neuroendocrine carcinoma; **d** Neoplastic cells are diffusely CD117 positive; **e** Nuclear positivity for MYC

#### AdCC with high-grade transformation (HG-AdCC)

Very rare cases of AdCC associated with different types of cancer have been reported [13]. The carcinomas associated with AdCC are frequently aggressive types, such as small cell carcinoma [14], invasive carcinoma of no special type [15]. In addition, rare cases of AdCC associated with adenomyoepithelioma (AME) have been reported [16].

#### In situ AdCC

Intraductal component of AdCC has been originally reported and illustrated by Rosen [17] as a polypoid intraductal growth.

#### Molecular data

Breast AdCC is usually devoid of ER/PR expression or HER2 amplification. In a minority of cases, rare epithelial cells can express ER [18]. Vranic et al. demonstrated that AdCC can express a rare ER isoform, namely ER-α36 [19]. Recently, Yiğit et al. [20] reported androgen receptor positivity in 7 cases of breast AdCC. The therapeutic value of these findings has yet to be investigated.

On molecular analyses breast AdCC frequently harbours *MYB-NFIB* fusion gene or *MYBL1* rearrangements or *MYB* amplification. These alterations can activate downstream signals that are potentially therapeutic targets [21]

Recently an unusual case of AdCC with adipocytic differentiation carrying BRAF mutation has been reported [22].

Most of the published data are based on C-AdCC. More recently, Massè et al. [23] compared 16 cases of C-AdCC with 17 cases of SB-AdCC by RNA-seq expression analysis, demonstrating 549 genes having a differential expression profile. Most importantly, *NOTCH* and *CREBBP* mutations were only detected in SB-AdCC. Notch signalling pathway can be a potential therapeutic target.

C-AdCC should be differentiated from several lesions. C-AdCC with prominent cribriform architecture can be easily differentiated from cribriform carcinoma of the breast as the latter is strongly and diffusely ER positive and lacks positivity for myoepithelial markers. The distinction between AdCC and collagenous spherulosis (CS) of the breast can be more challenging. CS is a benign condition, characterized by spherules of basement membrane in association with florid epiheliosis/usual ductal hyperplasia. Diffuse and strong positivity for several myoepithelial markers, together with absence of CD117 and MYB positivity favour the diagnosis of CS [24, 25].

C-AdCC with tubular growth pattern should be differentiated from microglandular adenosis (MGA) [26]. AdCC tubular variant is composed of epithelial and myoepithelial cells, both being evident by the application of appropriate immunohistochemical markers, at variance with MGA that is composed of epithelial cells only.

SB-AdCC can simulate neuroendocrine carcinoma. The differential diagnosis can be easily made based on immunohistochemistry as neuroendocrine markers are completely negative in SB-AdCC. Differentiation from conventional TNBC could be more difficult, and the diagnosis of SB-AdCC should be based on the presence of at least focal features of C-AdCC, strong and diffuse expression of MYB by immunohistochemistry or MYB-NFIB rearrangement by FISH [27].

The prognosis of AdCC mostly depends on the stage at presentation and AdCC variants. C-AdCC usually carries a good prognosis, despite the triple-negative phenotype. Almost all studies but one [28] demonstrated that C-AdCC recurrences or progression to metastases are exceedingly rare. Nevertheless, care should be taken to radically excise AdCC. We observed a case of C-AdCC recurring as HG-AdCC after 11 years. An 84-year-old woman presented in 2008 with a 5.5-cm tumour mass affecting the left breast. After the diagnosis of C-AdCC, the patients underwent surgery. The tumour was excised, but the margins were not clear. Due to the advanced age of the patient and low malignant potential tumour, a wait and see approach was advised. In 2019, 11 years later, the patient presented with a huge tumour mass, involving the whole residual breast. On histology the AdCC showed features of SB-AdCC and of HG-AdCC, in addition to features of C-AdCC (Fig. 1, Supplementary materials).

Therefore, radical surgery is considered the treatment of choice. Recurrences and metastases can occur in SB-AdCC [2, 3, 12].

The prognosis of AdCC with associated other types of carcinoma depends mainly on the associated malignancy.

### Adenomyoepithelioma and its malignant counterpart

Adenomyoepithelioma (AME), first described by Hamperl in 1970 [29], is a rare biphasic neoplasm characterized by the proliferation of ductal epithelial cells and myoepithelial cells [2]. Since the original description, numerous papers on AME have been reported [30]. Nevertheless, due to its rarity, most papers were single case reports or small series. Only rare studies were based on series larger than 15 cases (Table 1 in supplementary files).

AME predominantly affects elderly women over 60 years and is rarely seen in males [31]. It usually presents as a solitary palpable mass or as screen detected small nodule. It can arise in the retroareolar region [2]. Rare cases of multicentric AME have been reported [32]. Mammography reveals round or oval mass with lobulated margins, sometimes associated with rough heterogeneous calcifications [33]. Ultrasound identifies hypoechogenic lesions. MRI exhibits isointensity on T1W1 and high signal intensity on T2W1 [34].

AME diagnosis can be very difficult on fine-needle aspiration cytology and sometimes it can simulate fibroadenoma. A more accurate diagnosis can be rendered on core needle biopsies [35].

Macroscopically, AMEs are solid, well-circumscribed, often multi-lobulated lesions. Some AMEs have a papillomatous component with focal cystic change and calcification. On the other hand, malignant AMEs (AME-M) are not well-circumscribed and larger tumours [36].

On histology, AME exhibits a variable morphological spectrum. Diagnosis is based on the classical proliferation of both epithelial and myoepithelial cells.

The correlation between histological variants and prognosis is controversial.

**Fig. 3:**
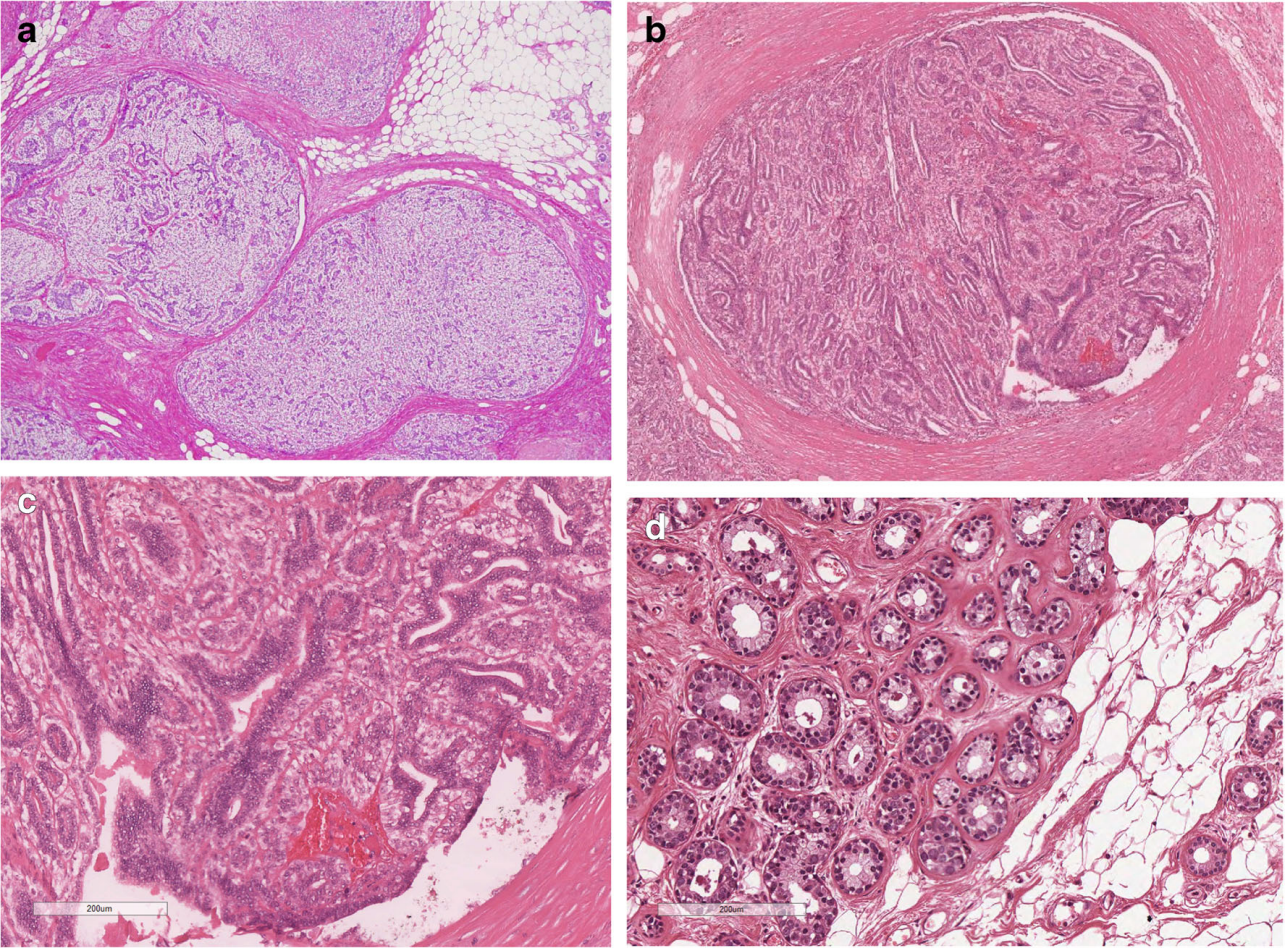
C-AME; **a** C-AME with lobulated architecture; **b** C-AME with intraductal papillary component; **c** At high power C-AME is composed of glandular strucutres lined by a inner layer of eosinophilic epithelial cells and by an outer layer of clear, myoepithelial cells; **d** C-AME with tubular architecture: at a variance of MGA, glands are lined by a double cell layer

Aspects of difficult interpretation may be encountered, which are summarized as follows:

*Classical AME (C-AME)* is the best known and illustrated form. It is characterized by glands lined by a double cell layers, the inner epithelial layer and the outer myoepithelial layer (Fig. 3). Based on architectural and cytological features, AME can be subdivided into: spindle cell, tubular, lobulated, papillary, and mixed [2, 30, 36–38]. Myoepithelial cells can have spindle shape or epithelioid appearance with clear cytoplasm. Apocrine, squamous, or sebaceous metaplasia can occur in luminal epithelial cells [36]. The tubular AME is characterized by small glandular structures which can grow by infiltrating the surrounding tissue. The tubular pattern has been illustrated in early papers [39, 40] and named as apocrine adenosis [40].

AME can exhibit a prominent *intraductal, papillary, growth* (IP-AME) [38]. IP-AME can grow along the ductal system of a breast lobe, resulting in a multinodular proliferation.

**Fig. 4:**
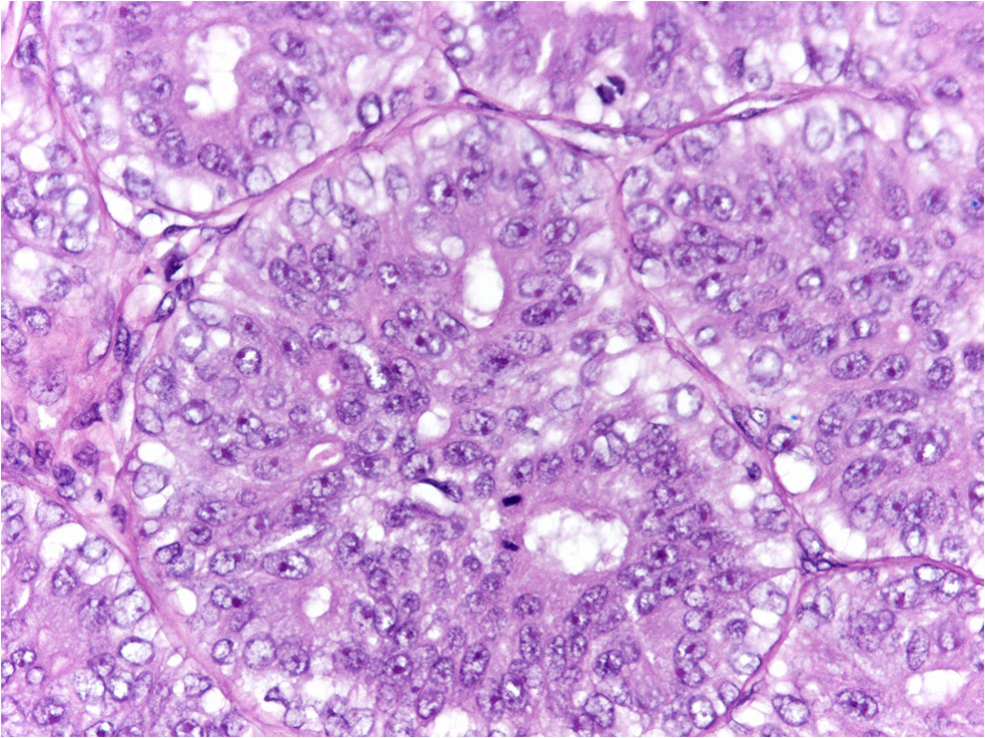
A-AME is composed of large cells, with atipical nucleous with coarse chromatin and evident nucleoli. Mitotic figures are frequent

*Atypical AME (A-AME)* (Fig. 4): Cytological atypia can be encountered in both epithelial and myoepithelial components. Criteria to define cytological atypia in breast AME have not been standardized. Applying the same criteria used in salivary glands [41], atypia should be considered when neoplastic cells (both epithelial or myoepithelial) are 3 times larger than normal cells (internal reference used: epithelial cells of normal mammary glands), have larger nucleus with coarse chromatin and irregular nuclear contour, nucleolus can be prominent. Cytological atypia is more frequently observed in the myoepithelial cells. A-AME can show features of myoepithelial overgrowth, when the myoepithelial cell proliferation is so prominent that it blurs the epithelial component.

**Fig. 5:**
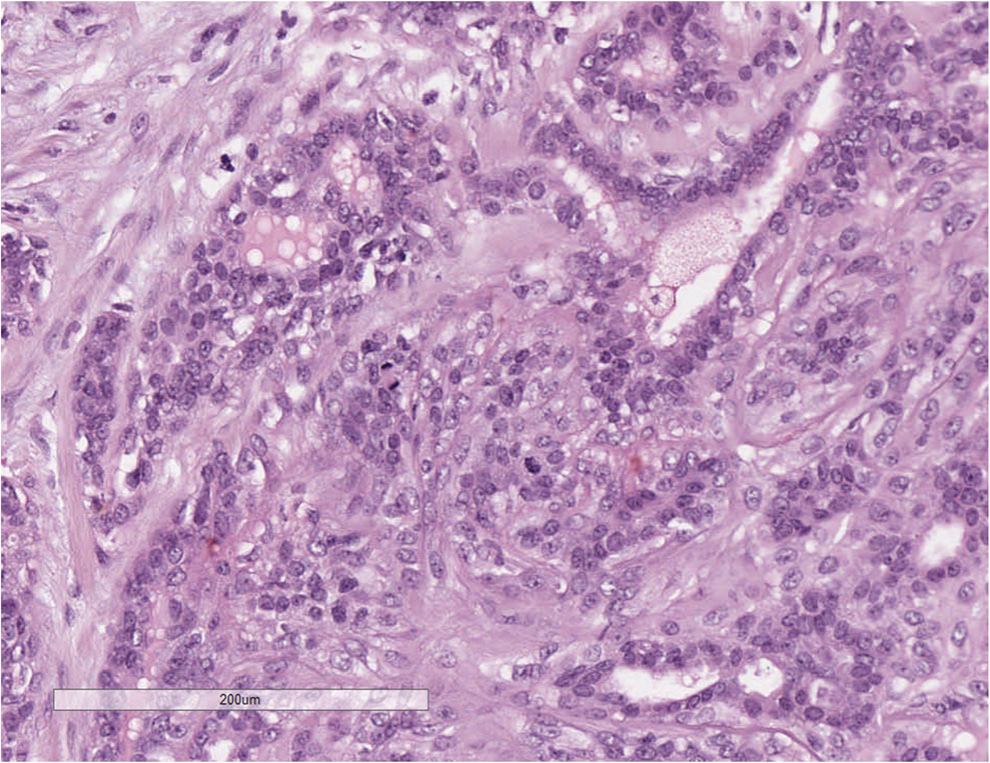
MA-AME has the same cell composition of C-AME, but presents frequent mitotic figures

*Mitotically active AME (MA-AME)* (Fig. 5). The number of mitotic figures is highly variable, ranging from none to more than 10 in 10 high-power fields (HPF) (summary in table 2 supplementary files). The definition of MA-AME should be applied in those C-AME, devoid of any atypia, with a high number of mitotic figures. The prognostic impact of the mitotic count in C-AME is not clear. The AFIP book [42] suggests that cases with more than 3 mitotic figures in 10 HPF are at higher risk of recurrence. Nevertheless, no clinical validation has been obtained on this number. Reviewing the reported cases (table 2 supplementary files), a high mitotic count has been reported mainly in AME with malignant transformation; therefore, the impact of mitotic count in otherwise C-AME is still unknown.

**Fig. 6:**
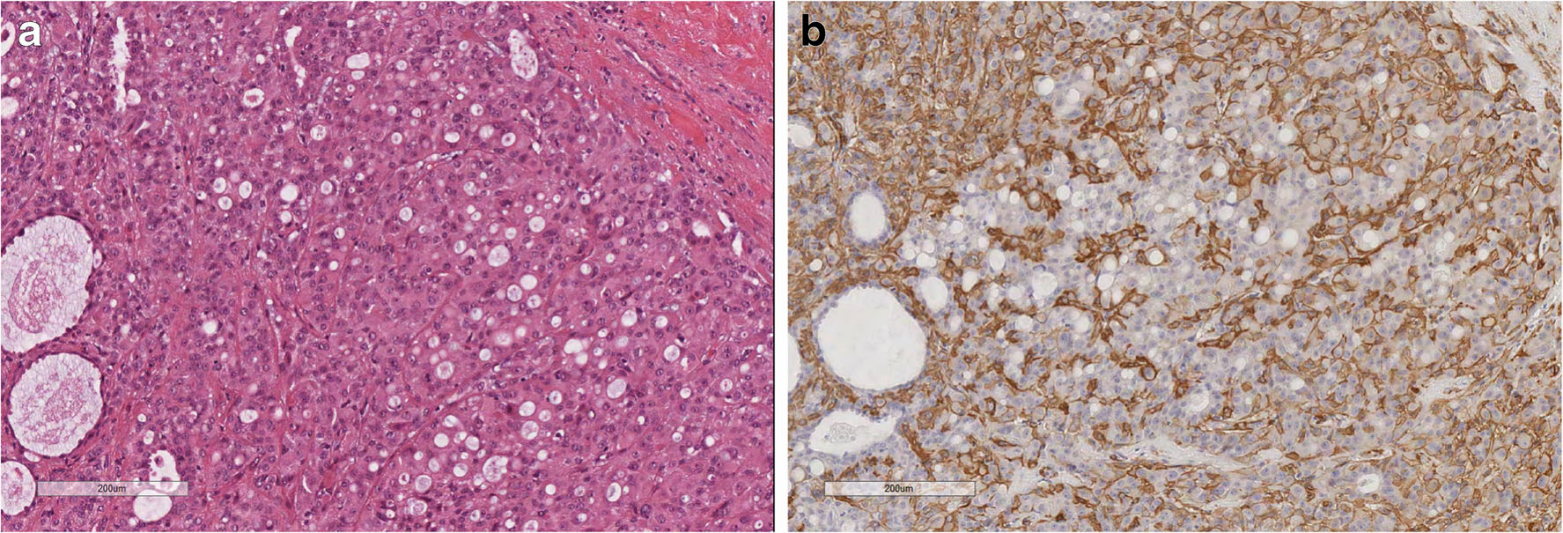
MIS-AME; **a** malignant transformation of the epithelial componenti is seen; **b** Smooth muscle actin evidences the myoepithelial cells compressed by the epithelial proliferation

*Malignant in situ AME (MIS-AME)*: According to Rakha et al. [30], MIS-AME should be diagnosed when the epithelial component of C-AME shows features of ductal carcinoma in situ. In MIS-AME, the malignant epithelial cells do not invade the stroma outside the AME mass (Fig. 6).

Conventional ductal or lobular carcinoma in situ can be associated with AME, but outside the AME neoplastic mass [43–45]. These latter cases should not be identified as MIS-AME.

**Fig. 7:**
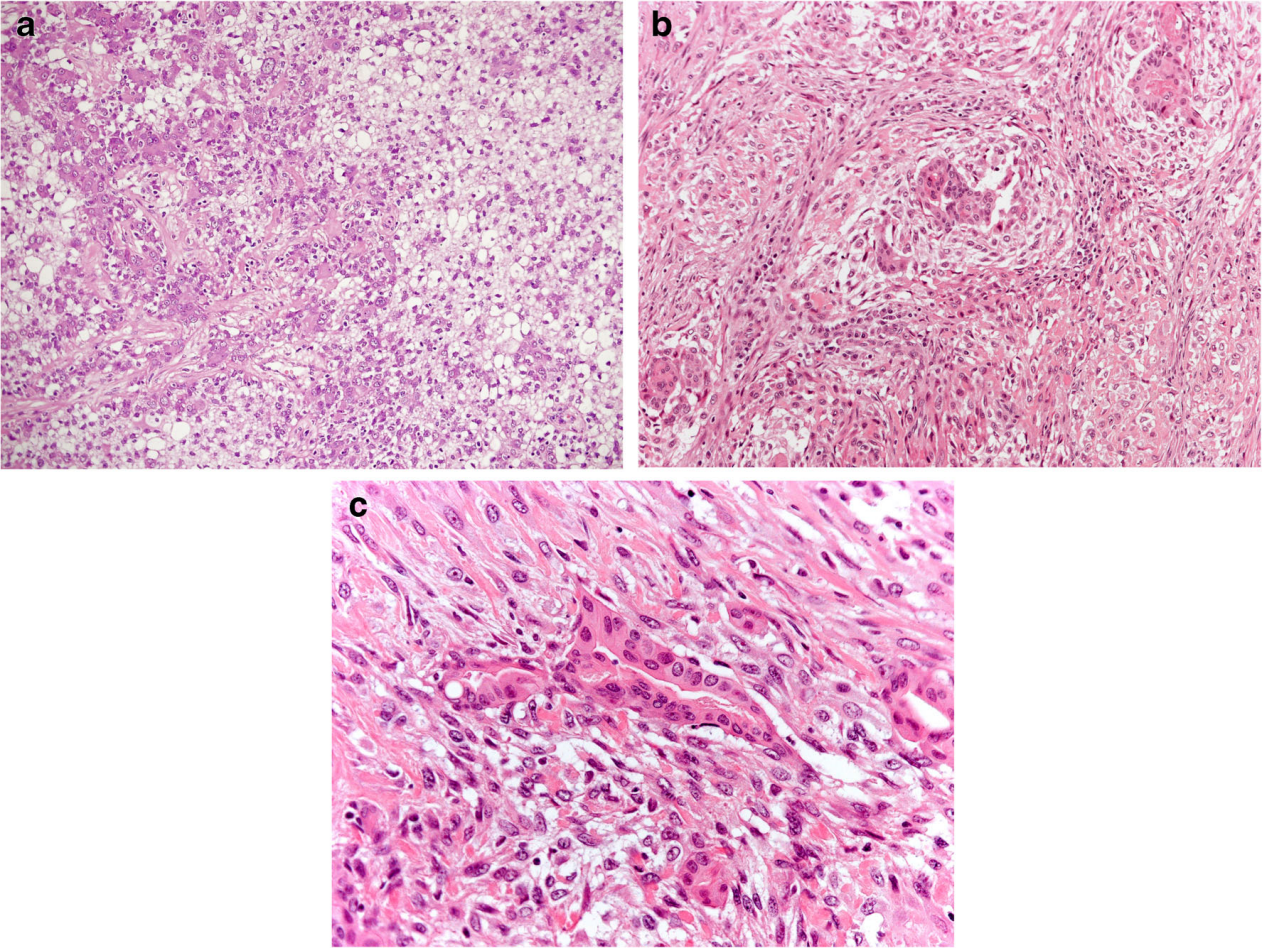
M-AME malignant transformation is evident in both, epithelial and myoepithelial component. **a**, **b** at low power view the myoepithelial cells predominate; **c** Both epithelial and myoepithelial cells show marked nuclear atypia

*Malignant AME (M-AME)* (Fig. 7): M-AME is diagnosed when either luminal epithelial and/or myoepithelial cell component becomes malignant. M-AME may develop from a long-standing C-AME [39] or de novo (Fig. 2 Supplementary files). Cases showing malignant transformation of both epithelial and myoepithelial cells are named epithelial-myoepithelial carcinoma [2].

M-AME is diagnosed when it shows marked cytologic atypia, numerous mitotic figures, necrosis, and infiltrative growth pattern. On some occasions, M-AME can exhibit a multinodular growth pattern.

When malignant transformation occurs in the epithelial component, it can show a great variety of morphological patterns. Specifically, the reported malignant components are low-grade adenosquamous and sarcomatoid carcinomas [46], multifocal adenosquamous carcinoma [47], matrix producing metaplastic carcinoma, acantholitic variant of squamous carcinoma, carcinosarcoma, spindle cell carcinomas [48–54], and mucoid carcinoma [46].

Dual differentiation of the neoplasm can be demonstrated by immunohistochemistry (IHC). Myoepithelial cells express p63, smooth muscle actin (SMA), cytokeratin 5/6 and 14, and CD10. Luminal epithelial cells express low molecular weight cytokeratins (Cam5.2, CK7, and CK8/18). Paradoxical expression of high-molecular weight cytokeratins in the epithelial component can be observed [55].

Oestrogen or progesterone receptor (ER, PR) can be positive or negative; Her2-neu is negative. Genetic alterations of AMEs differ according to their ER status. Oestrogen receptor-positive AMEs have mutations in phosphoinositide 3-kinase (PI3K) pathway genes, whereas ER-negative AMEs usually harbour concurrent mutations affecting the HRAS Q61 hotspot and PI3K pathway genes [56–60]. However, a recent ER-positive AME has been reported presenting both HRAS and PIK3CA mutations [60]. EGFR gene amplification has been recently reported [58].

Malignant transformation can alter ER expression status in AME as reported in one case showing ER-positive AME associated with ER-negative carcinoma [55].

Pleomorphic adenoma, adenoid cystic carcinoma, and low-grade adenosquamous carcinomas are included in the differential diagnosis.

Pleomorphic adenoma of the breast similar to AME is a biphasic tumour, characterized by glandular structures lined by a double cell layers (epithelial and myoepithelial) in chondromyxoid matrix [2]. At variance with C-AME, myoepithelial cells in pleomorphic adenoma glands are flat and not prominent. Both adenoid cystic carcinomas and low-grade adenosquamous carcinomas have infiltrative growth pattern. Adenoid cystic carcinomas usually have a cribriform pattern and basaloid myoepithelial cells. Low-grade adenosquamous carcinomas typically have abundant desmoplastic stroma and less myoepithelial component [60]. Nevertheless, all these tumours are part of the spectrum of breast tumours composed of epithelial and myoepithelial cells; therefore, their features can merge. Mixed tumours showing features of C-AME associated with pleomorphic adenoma [31], C-AME and adenoid cystic carcinoma [61], and C-AME and low-grade adenosquamous carcinoma [46] have been reported.

C-AME with papillary growth must be differentiated from benign intraductal papilloma. Intraductal papilloma can show focal myoepithelial hyperplasia, therefore rendering this differential diagnosis quite subjective and challenging. C-AME with papillary growth should be diagnosed when myoepithelial hyperplasia is prominent and diffuse in the whole lesion. Recently, monoclonal antibodies recognizing Q61R mutant NRAS and KRAS have been generated and demonstrated to stain positive in most ER-negative C-AME [57]. The application of these antibodies, where available, can be of help in the diagnosis of ER-negative C-AME. If immunohistochemistry is not available, diagnosis should be based on the presence of prominent myoepithelial layer.

Pure metaplastic carcinoma is the major differential diagnosis in de novo M-AME as both can have biphasic components. The association with C-AME or A-AME would be the clue to diagnose M-AME. Another evidence to exclude metaplastic carcinoma is HRAS Q61R mutations which occur with *PIK3CA* or *PIK3R1* mutations, recently found to be highly specific for ER-negative M-AME [56]

C-AMEs have a favourable clinical course. Nevertheless, local recurrences can occur. Some AMEs with multinodular growth pattern may have local recurrence. Existence of satellite nodules and peripheral intraductal growth seem to be associated with recurrence [36]. Rare cases of C-AME devoid of any atypical features at presentation developed distant metastases [62, 63]. Thus, most AMEs are cured by complete surgical resection with clear margins but follow-up should be advised for all patients with AME.

Other parameter indicating tendency to recur is the infiltrative growth pattern.

AMEs with large size, pure solid growth, myoepithelial component overgrowth, invasive growth pattern, cytological atypia, and abundant mitotic activity are suggestive of malignant transformation [30, 36].

M-AME has local recurrence and metastatic potential. The histologic subtype and the grade of the invasive carcinoma determine the prognosis. Lung is the most common site of metastasis followed by liver, bone and brain while lymph node metastases are rare [30].

## Tumours with pure epithelial differentiation

### Mucoepidermoid carcinoma (MEC)

Breast MEC is a carcinoma composed of mucoid, epidermoid, and intermediate cells [2]. It shares the same features of the salivary gland counterpart.

Clinical presentation is non-specific. MEC arises in adult women; any breast quadrant can be affected. It presents as a nodule, with well-defined margins and cystic component. The imaging appearance can mimic a benign lesion [64].

Fine-needle aspiration cytology can be difficult to interpret. In cases of high-grade MEC, the presence of atypical cells with both glandular and epidermoid differentiation can lead to the correct diagnosis of carcinoma [65, 66]. Ancillary studies can help to better specify the tumour histotype. The pre-operative diagnosis can be made on core needle biopsy. The most important clue is to keep in mind that salivary gland-like tumours can occur in the breast.

**Fig. 8:**
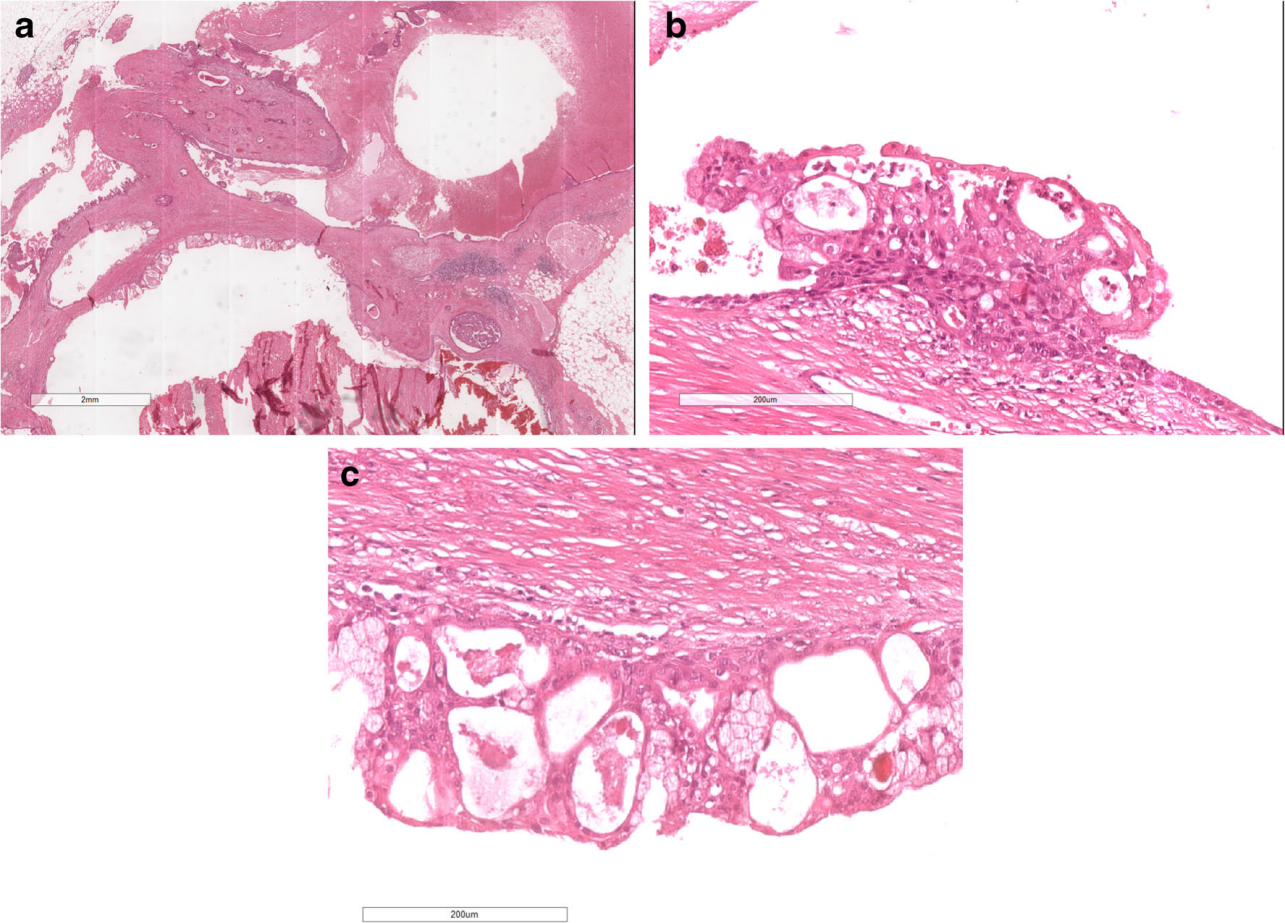
MEC: **a** Low power view of low-grade mucoepidermoid carcinoma with prominent cystic component; **b** and **c** at higher power the cysts are lined by epithelium with mucoid and epidermoid cells

On histology (Fig. 8), MEC is composed of mucous, intermediate and epidermoid cells. True keratinization with squamous pearls should be excluded.

Breast MEC morphology varies according to the grade. Grade should be performed either according to the breast criteria or applying salivary gland criteria [67].

Low-grade MEC usually shows a prominent cystic component and well-defined margins. The cysts are lined by mucous and eosinophilic cells. Mucous cells can, sometimes, have a signet ring appearance. The neoplastic cells are usually arranged in nests, with the mucous and eosinophilic cells located in the centre and epidermoid cells located at the periphery. The most peripheral cell layer is composed of basaloid cells.

High-grade MEC shows the same cell composition, but with higher degree of solid architecture and cytologic atypia. Necrosis can be present. Atypical mitotic figures are frequent.

Cases of intermediate grade can be encountered.

In situ component can be seen, characterized by an intraductal papillary proliferation, showing the classical mucous cells intermingled with the epidermoid cells.

Immunohistochemistry can help to demonstrate the peculiar architecture and cell composition. High-molecular weight cytokeratins (such as CK14 and CK5/6) and p63 typically stain the epidermoid and the basaloid cells located at the peripheral part of the neoplastic nests and cysts. Low molecular weight CK (such as CK7) stains the centrally located mucous and the eosinophilic cells [64, 67, 68].

Breast MEC can be positive for the anti-mitochondrial antigen [67].

Breast MEC do not express hormone receptors nor have HER2 amplification.

Salivary gland MEC typically shows *CRTC1/MAML2* translocation. Rare cases of breast MEC were shown to have MAML2 rearrangements [69–71].

Breast MEC should be differentiated from all other carcinomas with divergent, adenosquamous differentiation and from metaplastic carcinomas with squamous differentiation. Differential diagnosis can be difficult, especially for the high-grade MEC.

The presence of true keratinization and squamous pearls should discourage the diagnosis of MEC.

Detection of MAML2 rearrangements can be of help [70] as they are absent in other breast carcinomas. When molecular tools are not available, detecting the typical alternation of cytokeratins (high molecular weight in the peripheral part and low molecular weight in the central part of the neoplastic nests) can help.

Prognosis largely depends on MEC grade [72]. Ye et al. [64] reviewed 41 cases of breast MEC reported in the literature. Axillary lymph node metastases can occur both in low- and high-grade MEC, while distant metastases have been described in high-grade cases only. None of the low-grade MEC caused patient’s death, while death occurred in 4 of 11 high-grade MECs. One case of low-grade MEC recurred with high-grade transformation [73]; nevertheless, the patient was alive with no evidence of disease 156 months after presentation.

### Acinic cell carcinoma (ACC)

Breast ACC is composed of cells showing features of serous acinar differentiation, characterized by intracytoplasmic eosinophilic zymogen granules [2].

Clinical presentation is non-specific. ACC affects mainly adult women, with only 1 male patient reported in the literature [74]. ACC can arise in any breast quadrants, presenting as an infiltrative nodule; size varies sometimes reaching large dimensions [75, 76]. One case of ACC arising in a fibroadenoma was reported [76].

Pre-operative findings can be difficult to differentiate from invasive carcinoma NST. Both on fine-needle aspiration and on core needle biopsy, attention must be paid to the intracytoplasmic secretory granules.

**Fig. 9:**
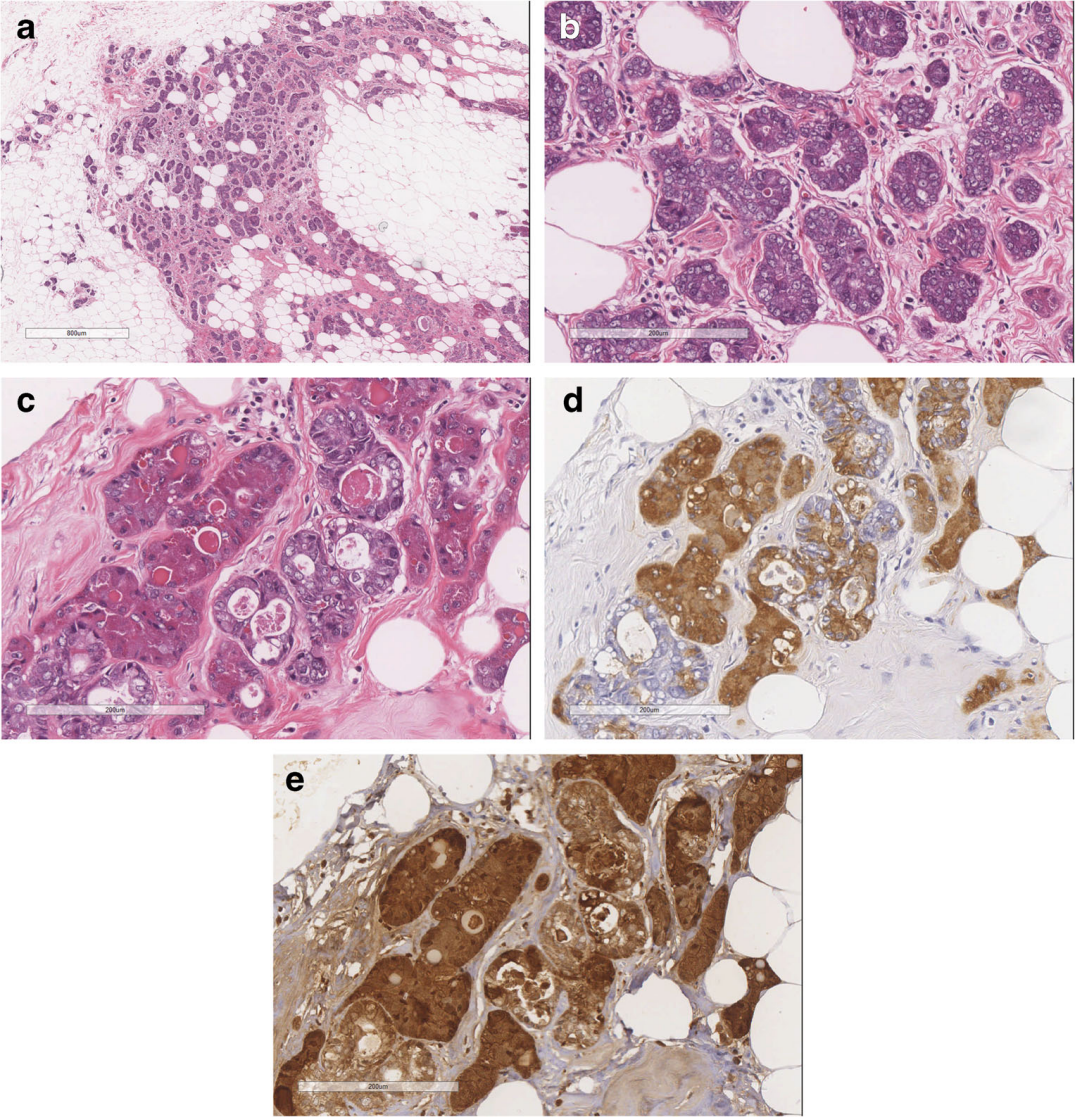
ACC; **a** ACC with prominent microglandular architecture is composed of small glands infiltrating the breast tissue. **b** the small glands are lined by atypical cells; **c** the neoplastic cells show a finely granular cytoplasm; **d** most of the neoplastic cells are EMA positive; **e** the neoplastic cells are strongly positive for Lysozyme

On histology (Fig. 9), breast ACC can exhibits a variety of architectural patterns, ranging from microcystic to solid. Histological diagnosis is mainly based on cytologic features. Neoplastic cells show abundant granular cytoplasm. Intracytoplasmic granules can be seen on H&E, but when not prominent, they are better appreciated on PAS with diastase.

ACC with microglandular architecture is characterized by small glandular structures lined by atypical cells, with granular cytoplasm. No myoepithelial cells or basement membrane are present. Areas of microglandular architecture can gradually merge with solid areas, composed of the same cell type.

In situ component can be present, showing features similar to duct carcinoma in situ, with comedo-type necrosis, but composed of cells with the typical granules.

Immunohistochemistry can help to reach the correct diagnosis. The typical immunohistochemical profile is the following: amylase, lysozyme, and alpha-1-antichimotrypsin positivity associated with strong and diffuse immunoreactivity for S-100 and EMA.

No hormone receptor positivity or HER2 amplification have been detected. On rare cases, androgen receptor positivity has been described [77].

The molecular profile of breast ACC is more similar to conventional TNBC than that of salivary gland ACC. Breast ACC is characterized by complex gene copy number alterations, recurrent *TP53* mutation and occasional *PIK3CA* hotspot mutations [78].

ACC should be differentiated from several benign and malignant breast lesions showing a microglandular architecture [26]. The diagnosis of ACC with microglandular pattern should be limited to those cases composed of neoplastic glands lined by atypical cells with intracytoplasmic serous granules, devoid of myoepithelial cells and basement membrane. The ACC glandular structures can be surrounded by fine capillary vessels [26]. One must pay attention not to confuse basement membrane of the capillaries with the basement membrane of the neoplastic glands.

The most controversial differential diagnosis is between ACC with microglandular architecture and microglandular adenosis [26]. Some authors consider the two entities as part of the same spectrum [78]. The presence of intracytoplasmic granules, nuclear atypia and mitotic figures (even if rare) should favour the diagnosis of ACC.

The relatively small number of cases reported in the literature together with the limited length of follow-up do not allow drawing firm conclusions on the prognosis. Despite these limitations, the cases reported to date seem to have good prognosis [3].

Axillary lymph node metastases were reported in 10 out of 31 cases and local relapse in 4 of 38 cases.

Distant metastases have been described in 4 patients (bone, liver, lung and meninges), 3 of whom died of disease [3]. It is important to note that metastases also occurred in cases that exhibited microglandular architecture on histology [79, 80].

### Tall cell carcinoma with reverse polarity (TCCRP)

TCCRP is an invasive carcinoma composed of elongated cells, with eosinophilic and finely granular cytoplasm, arranged in solid and solid-papillary patterns. The neoplastic cells show nuclear polarity toward the periphery of the neoplastic solid nests or papillae [2].

TCCRP was originally described in 2003 by Eusebi et al. [81] who defined it as “Breast tumour resembling the tall cell variant of papillary thyroid carcinoma”, due to its striking similarity with the thyroid papillary carcinoma. Despite the morphological similarities, no immunohistochemical markers in common with thyroid carcinoma or RET/PTC alterations were detected [82]. Papers published in the following years demonstrated that TCCRP is a unique entity of breast primary [83].

Several papers appeared in the literature proposed different names for this entity, thus creating an unclear terminology. Therefore, the latest edition of WHO classification proposed the unifying terminology of TCCRP [2].

TCCRP is a very rare breast tumour, affecting adult female patients and presenting as a palpable breast nodule. Nodules can affect all breast quadrants, usually being 1–2 cm in greatest dimension. Larger nodules have been reported [83, 84]

Needle core biopsy shows the same features as observed in surgical specimens; therefore, the same diagnostic criteria should be applied.

**Fig. 10:**
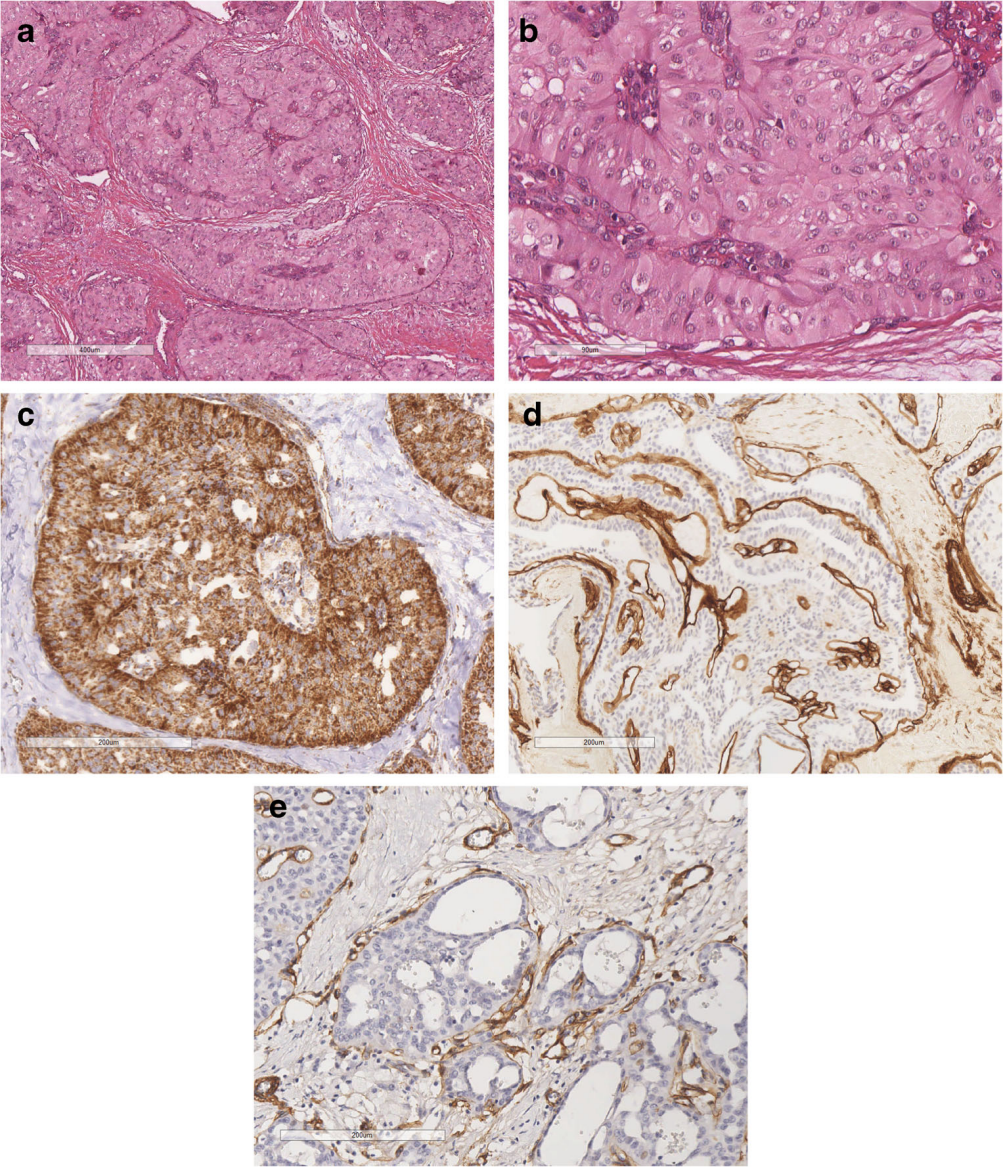
TCCRP; **a** TCCRP is composed of neoplastic nests with solid-papillary architecture; **b** at higher power the neoplastic cells are columnar, have granular and eosinophilic cytoplasm. Nuclei have fine chromatin and grooves; **c** Anti-mitochondrial antigen is strongly positive. Positivity is is condensed at the basal pole of the neoplastic cells; **d** Collagen IV surrounds the neoplastic nests, evidencing the basal membrane of small capillaries; **e** CD31 evidences small capillaries surrounding the neoplastic nests

On histology (Fig. 10), TCCRP is characterized by solid or solid-papillary architecture. Margins are pushing and multilobated. The neoplastic nests are composed of tightly packed papillae, resulting in solid appearance. Areas with follicular architecture can be encountered. Follicular structures are filled with dense, eosinophilic secretion; foamy macrophages are focally present.

The most striking feature of TCCRP is the typical cytological appearance of the neoplastic cells. Neoplastic cells are columnar in shape, with eosinophilic and finely granular cytoplasm.

The nucleus is oval, with fine and clear chromatin, nuclear grooves and pseudoinclusions, occupying about one third of the cell. The neoplastic cells are oriented perpendicular to the fibrovascular axis and have a basal pole, located on the opposite side of the fibrovascular pole (“reverse polarity”). Fine capillaries are arranged in a “garland-like fashion” around the neoplastic nests.

Mitotic figures are very rare, as well as vascular and perineural invasion. No necrosis is seen.

TCCRP shows expression of low and high-molecular weight cytokeratins, and markers of mammary epithelium origin, such as GATA3, mammaglobin, and GCDFP-15. In contrast, markers of thyroid origin, such as TTF1 and thyroglobulin are consistently negative in all cases [81, 83]. Most TCPCRPs show strong positivity for anti-mitochondrial antigen [83, 84]. Mitochondria are condensed at the basal pole of the neoplastic cells, therefore highlighting the “reverse polarity”.

In situ component has been described on rare occasions [84]. In most cases, myoepithelial markers fail to reveal a myoepithelial layer.

Hormone receptors and HER2 are usually negative. On rare occasions, a minority of the neoplastic cells can stain for oestrogen receptor [83]. Molecular studies detected isocitrate dehydrogenase 2 (*IDH2*) [85–87] and phosphatidylinositol 3-kinase catalytic alpha (*PIK3CA*) hotspot mutations [87].

Metastases from thyroid papillary carcinoma are easily excluded by the negativity of the thyroid markers. Negativity for markers of endocrine differentiation excludes the diagnosis of endocrine carcinoma.

Differential diagnosis can be more difficult with papillomas of the breast, which can focally present features similar to those of TCPCRP. Different from papillomas, TCCRP shows the peculiar features throughout the entire nodule. Myoepithelial cells are absent in TCCRP, while present in papillomas. In addition, the strong and basally located immunoreactivity for the anti-mitochondrial antigen is typical of TCPCRP and can help to reach the correct diagnosis.

TCPCRP is a low-grade carcinoma, with indolent clinical course in most of the cases. Two cases with lymph node metastases have been reported [83, 84]; both patients were alive and well after surgical removal of the tumour and the lymph node metastasis. Only one case behaved in an aggressive fashion [88].

### Secretory carcinoma

Secretory carcinoma represents an exceedingly rare breast neoplasm [89] with a predilection for children and young adults, which explains the term “juvenile breast carcinoma” adopted by McDivitt and Stewart in 1966 [90]. Nevertheless, secretory carcinoma can occur at any age and it is classified in the WHO blue book based on its distinctive histopathological features [2].

**Fig. 11:**
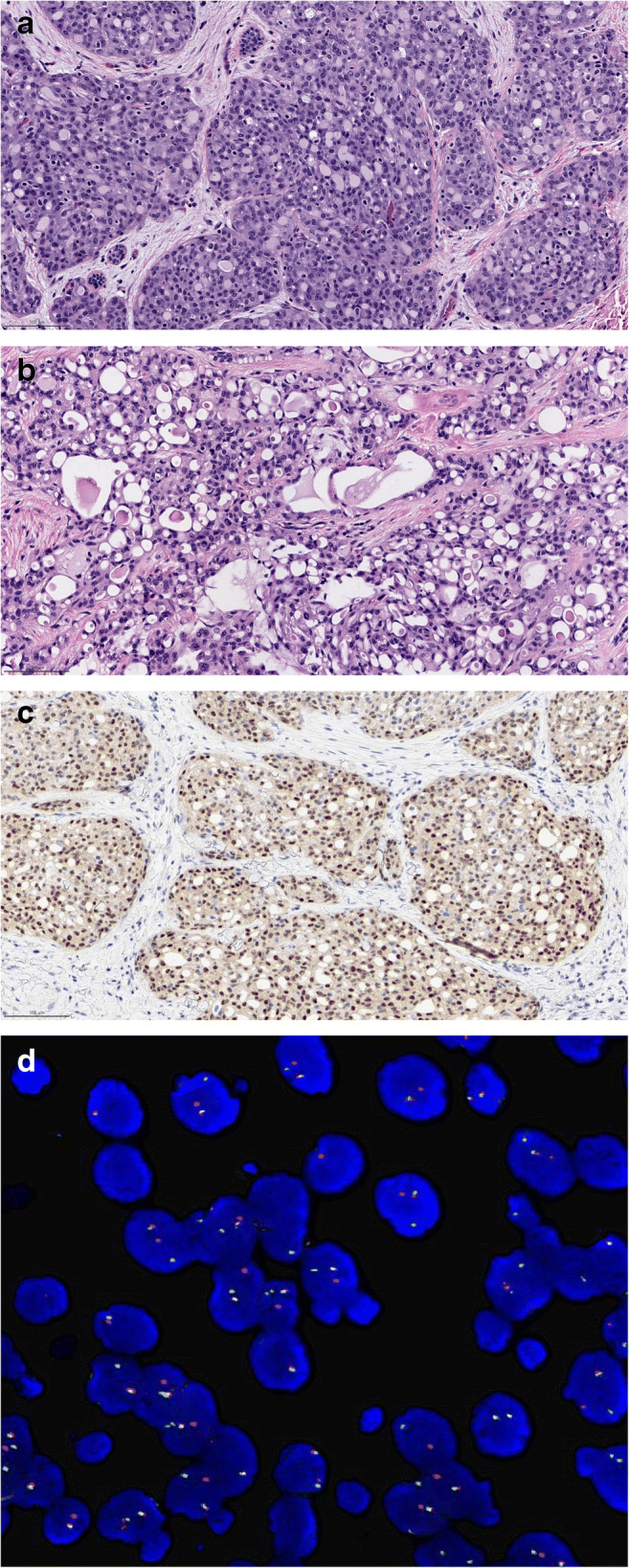
Secretory Carcinoma; **a** Secretory carcinoma shows a prominent microcystic pattern of growth; **b** at higher power, the neoplastic cells show vacuolated cytoplasm; **c** The chimeric NTRK3 protein is over-expressed; **d** Fluorescent in situ hybridzation evidences the ETV6-NTRK3 fusion

Histologically (Fig. 11), this tumour typically shows a microcystic growth pattern but tubular, solid, and even papillary features can be encountered and patterns are often admixed. Tumour cells are polygonal with eosinophilic granular or vacuolated cytoplasm. Nuclear pleomorphism is usually mild to moderate.

At the molecular level, the presence of a balanced translocation, t(12;15)(p13;q25), leading to the *ETV6*-*NTRK3* fusion gene is pathognomonic of secretory carcinomas, either of the breast or of the salivary gland [91, 92]. Overexpression of the chimeric *NTRK3* protein has been reported to be typically encountered in the nuclei of secretory carcinomas, as detected by panTRK antibodies [92, 93].

Although most of secretory carcinoma patients present with local disease and are surgically managed, this genetic alteration may be of interest for those rare patients developing distant metastases. TRK inhibitors have shown substantial and durable responses [94] and are FDA and EMA approved for metastatic patients as well as for patients with unresectable local disease.

A cohort of secretory carcinoma with aggressive behaviour has been recently reported, showing the presence of *TERT* promotor mutations and loss of *CDKN2A/B*, in addition to the typical *ETV6-NTRK3* fusion [95].

The spectrum of differential diagnoses for this tumour may encompass carcinomas with apocrine differentiation, acinic cell carcinoma, and tall cell carcinoma with reversed polarity. In these instances, investigation of the *ETV6-NTR3* fusion is of diagnostic support.

Most cases of secretory carcinomas are of triple-negative phenotype, however the clinical course of patients affected by secretory carcinoma is typically indolent, with an excellent prognosis even in patients with ipsilateral axillary lymph node metastases [95–98]. Although remarkably rare (reviewed in reference [96]) distant metastases can occur and advanced stage patients have a dismal prognosis.

## Conclusions

This review highlights the main histological, immunohistochemical, and molecular features of TNBC of low malignant potential. TNBC with low malignant potential are indeed rare breast tumours, each with unique features. Knowledge on their histological features and clinical behaviour are of utmost importance for pathologists, to avoid overdiagnoses and overtreatment. These lesions need to be appropriately discussed at the multidisciplinary team meetings, to explain differences from common forms of TNBCs.

## Supplementary Information

ESM 1 C-AdCC recurring as HG-AdCC; a) the tumor as appeared at presentation. It showed features of C-AdCC with prominent cribriform architecture; b) at higher power the tumor showed the typical features of C-AdCC; c) recurrent AdCC after 11 years. The tumour shows different features in the different areas; d) features of AdCC classical variant are still present. Here the AdCC shows mainly cribriform appearance; e) the classical features merge with solid-basaloid areas; f) areas composed of markedly atypical cells, with clear cytoplasm are present. These latter cells represent features of high-grade transformation; g) neoplastic cells of the high grade transformation areas are strongly ER positive, while the AdCC SB and classical areas are ER negative. (PNG 18079 kb)

High Resolution Image (TIF 2016 kb)

(PNG 15058 kb)

High Resolution Image (TIF 2016 kb)

(PNG 10301 kb)

High Resolution Image (TIF 13198 kb)

(PNG 9054 kb)

High Resolution Image (TIF 11744 kb)

(PNG 7199 kb)

High Resolution Image (TIF 9432 kb)

(PNG 10044 kb)

High Resolution Image (TIF 13439 kb)

ESM 2 AME showing transition between benign and malignant features. a) At low power the tumour is composed of a central solid component and a peripheral tubular component; b) the peripheral, tubular component shows small glands, lined by an inner epithelial and an outer myoepithelial layer; c, d) atypical areas intermingled with tubular areas; e) the bulk f the tumor is composed of highy atypical cells; a central area of necrosis is present. (PNG 14150 kb)

High Resolution Image (TIF 2272 kb)

(PNG 14561 kb)

High Resolution Image (TIF 2272 kb)

(PNG 15814 kb)

High Resolution Image (TIF 2272 kb)

(PNG 15269 kb)

High Resolution Image (TIF 2272 kb)

(PNG 16105 kb)

High Resolution Image (TIF 2272 kb)

ESM 3 (DOC 238 kb)

ESM 4 (DOC 112 kb)

ESM 5 (DOC 73 kb)

## Data Availability

Software application or custom code: not applicable.
