# Supplementary figures and images for: Triple-negative breast carcinomas of low malignant potential: review on diagnostic criteria and differential diagnoses
Source: Virchows Arch. 2021 Aug 30;480(1):109–26. doi: 10.1007/s00428-021-03174-7 (PMC8983547; doi:10.1007/s00428-021-03174-7)

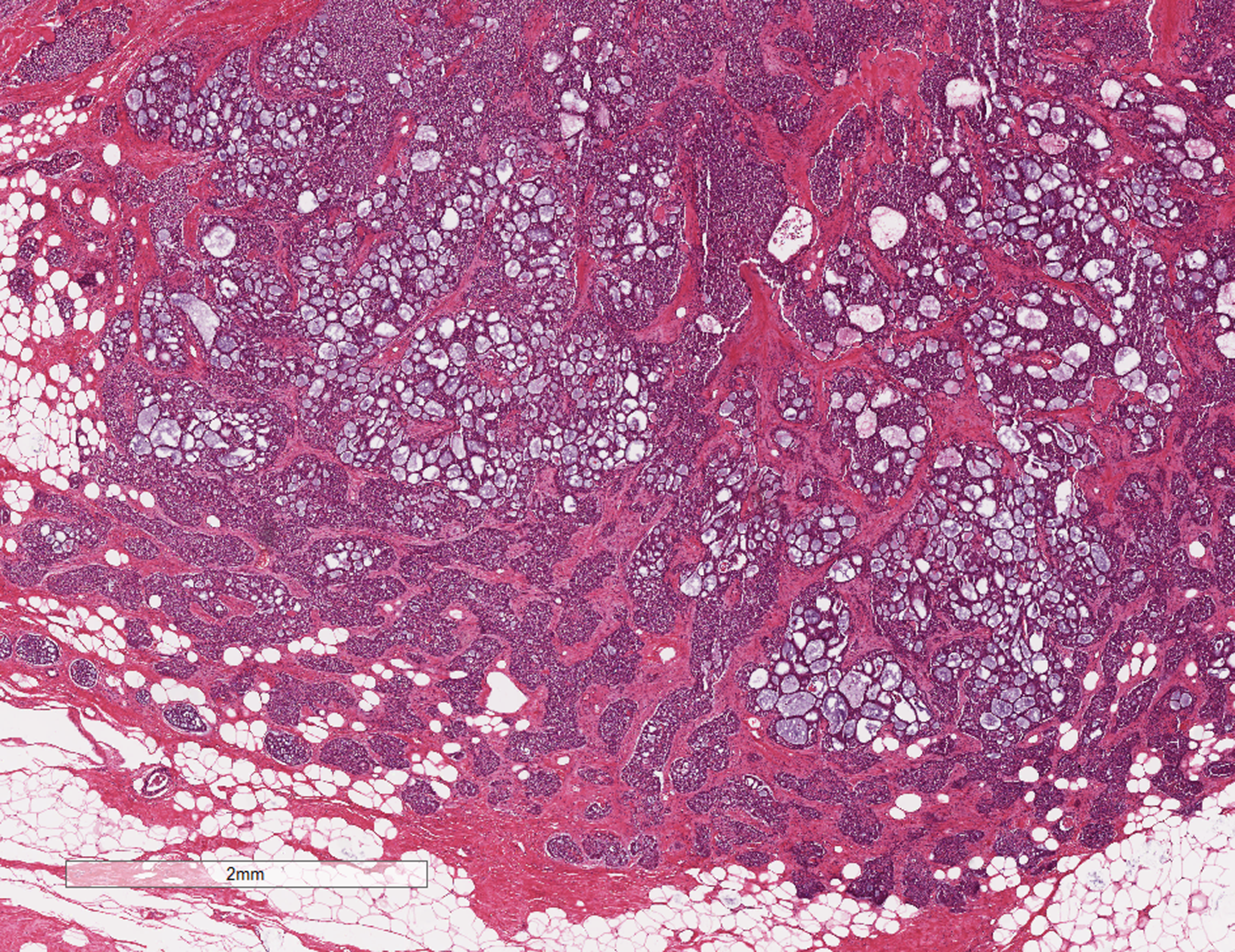

Supplement: Supplementary file 1 — C-AdCC recurring as HG-AdCC; a) the tumor as appeared at presentation. It showed features of C-AdCC with prominent cribriform architecture; b) at higher power the tumor showed the typical features of C-AdCC; c) recurrent AdCC after 11 years. The tumour shows different features in the different areas; d) features of AdCC classical variant are still present. Here the AdCC shows mainly cribriform appearance; e) the classical features merge with solid-basaloid areas; f) areas composed of markedly atypical cells, with clear cytoplasm are present. These latter cells represent features of high-grade transformation; g) neoplastic cells of the high grade transformation areas are strongly ER positive, while the AdCC SB and classical areas are ER negative. (PNG 18079 kb) [file 428_2021_3174_Fig12_ESM.png]

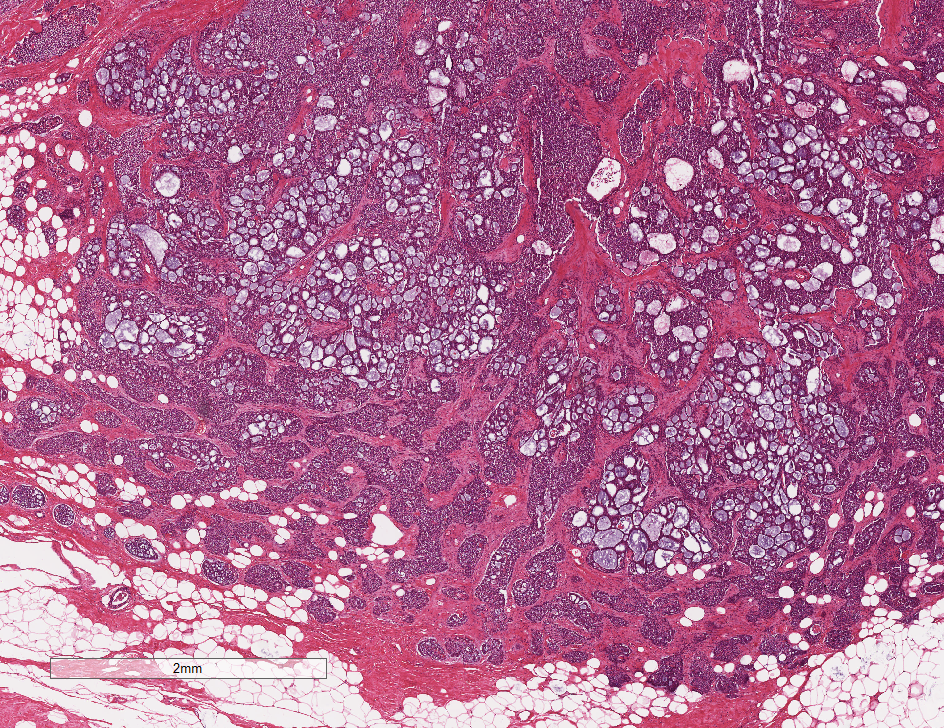

Supplement: Supplementary file 2 — High Resolution Image (TIF 2016 kb) [file 428_2021_3174_MOESM1_ESM.tif]

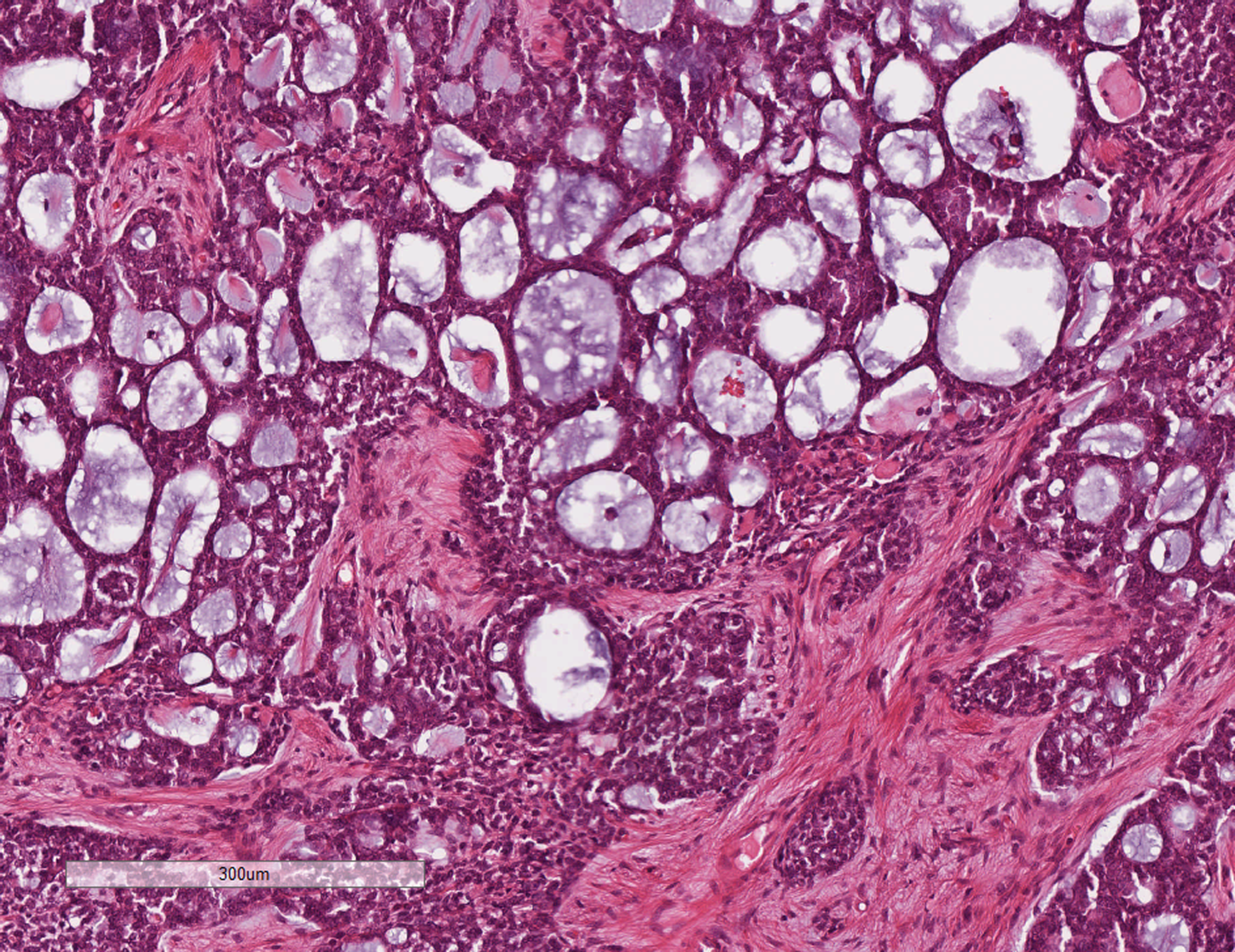

Supplement: Supplementary file 3 — (PNG 15058 kb) [file 428_2021_3174_Fig13_ESM.png]

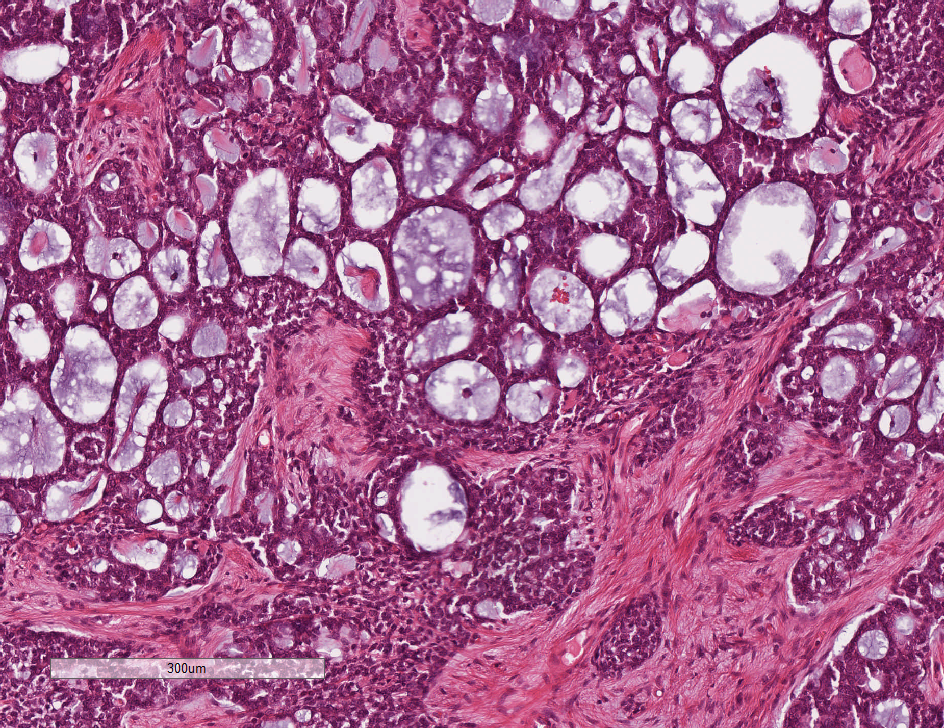

Supplement: Supplementary file 4 — High Resolution Image (TIF 2016 kb) [file 428_2021_3174_MOESM2_ESM.tif]

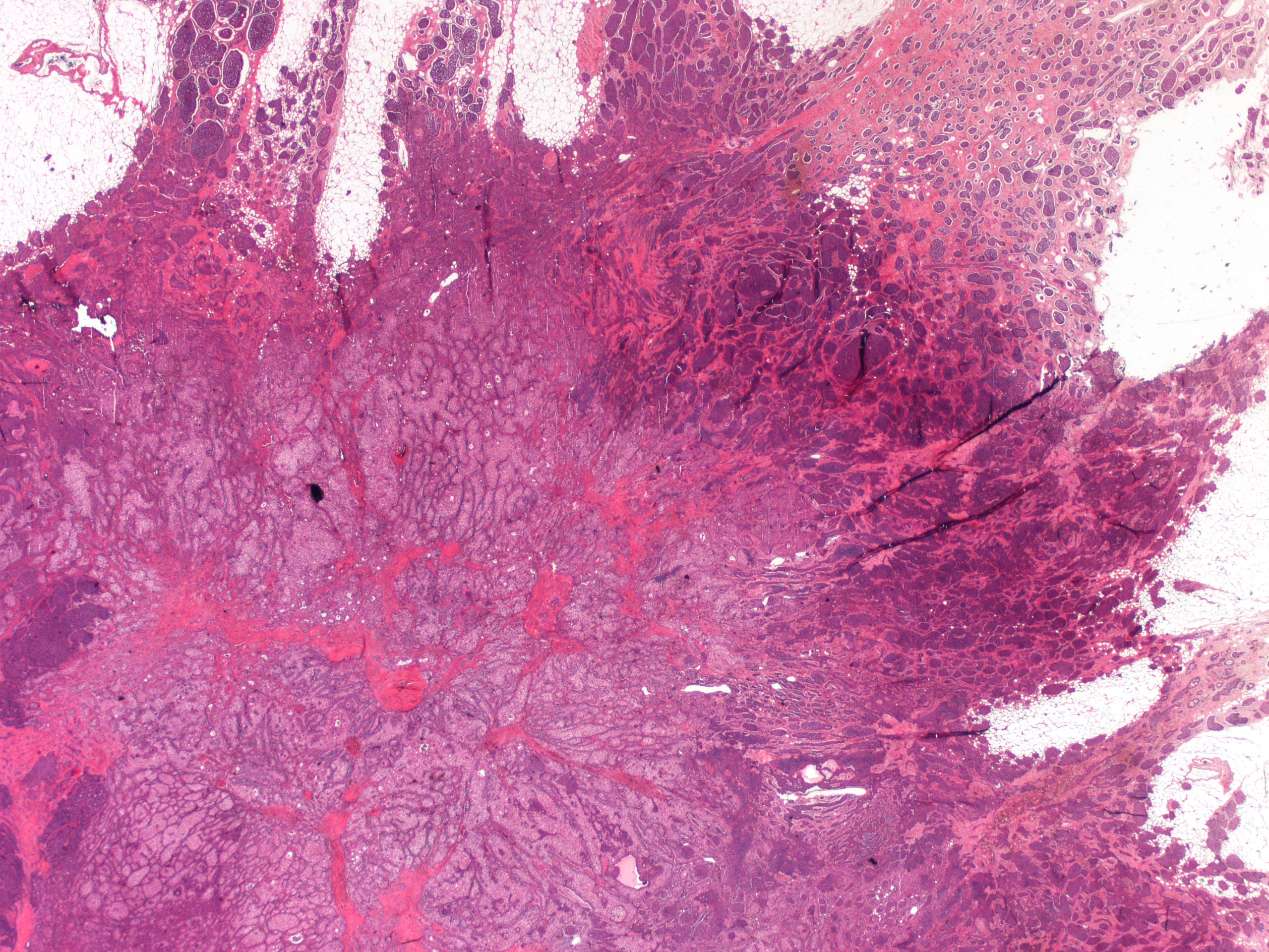

Supplement: Supplementary file 5 — (PNG 10301 kb) [file 428_2021_3174_Fig14_ESM.png]

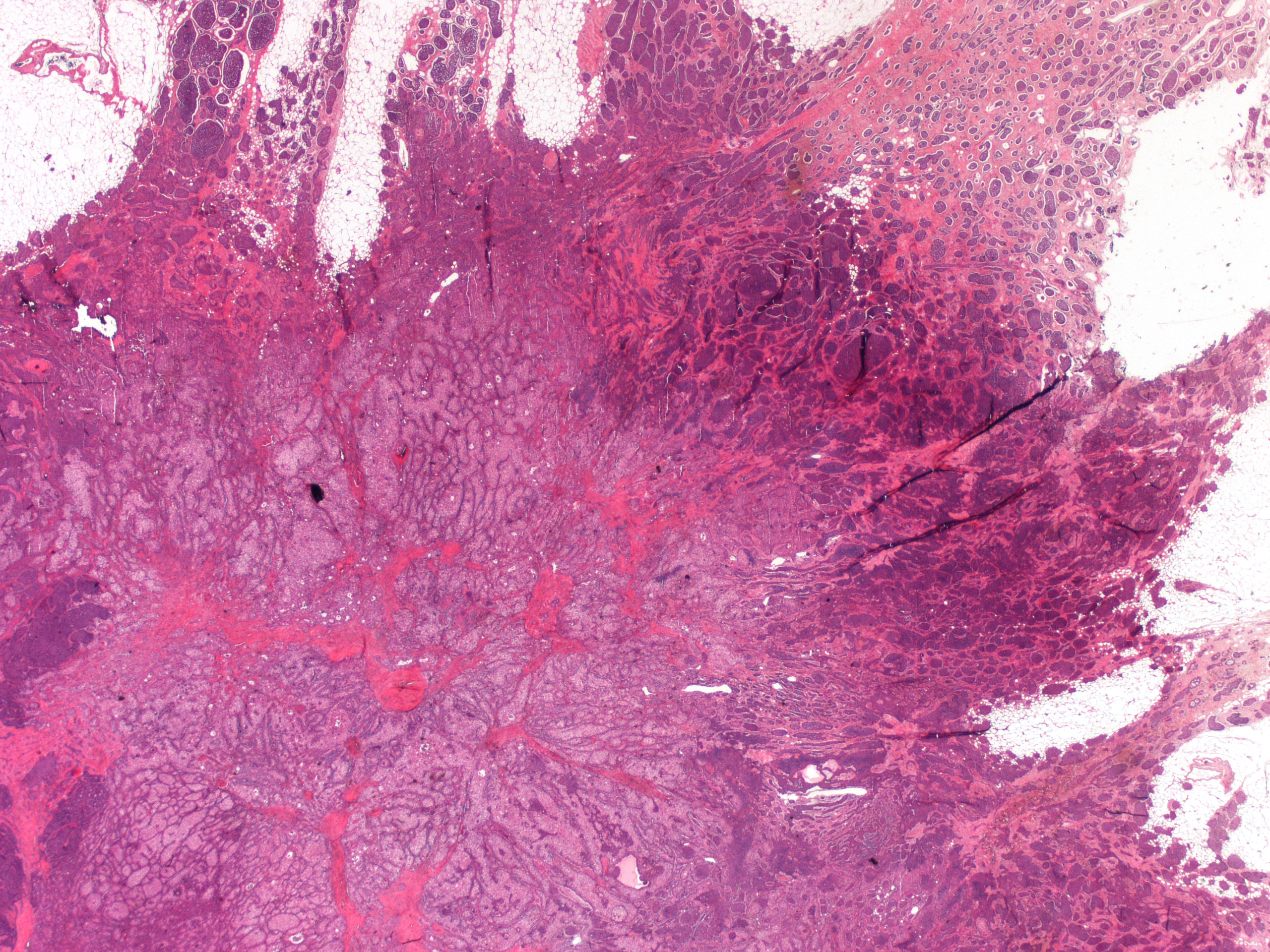

Supplement: Supplementary file 6 — High Resolution Image (TIF 13198 kb) [file 428_2021_3174_MOESM3_ESM.tif]

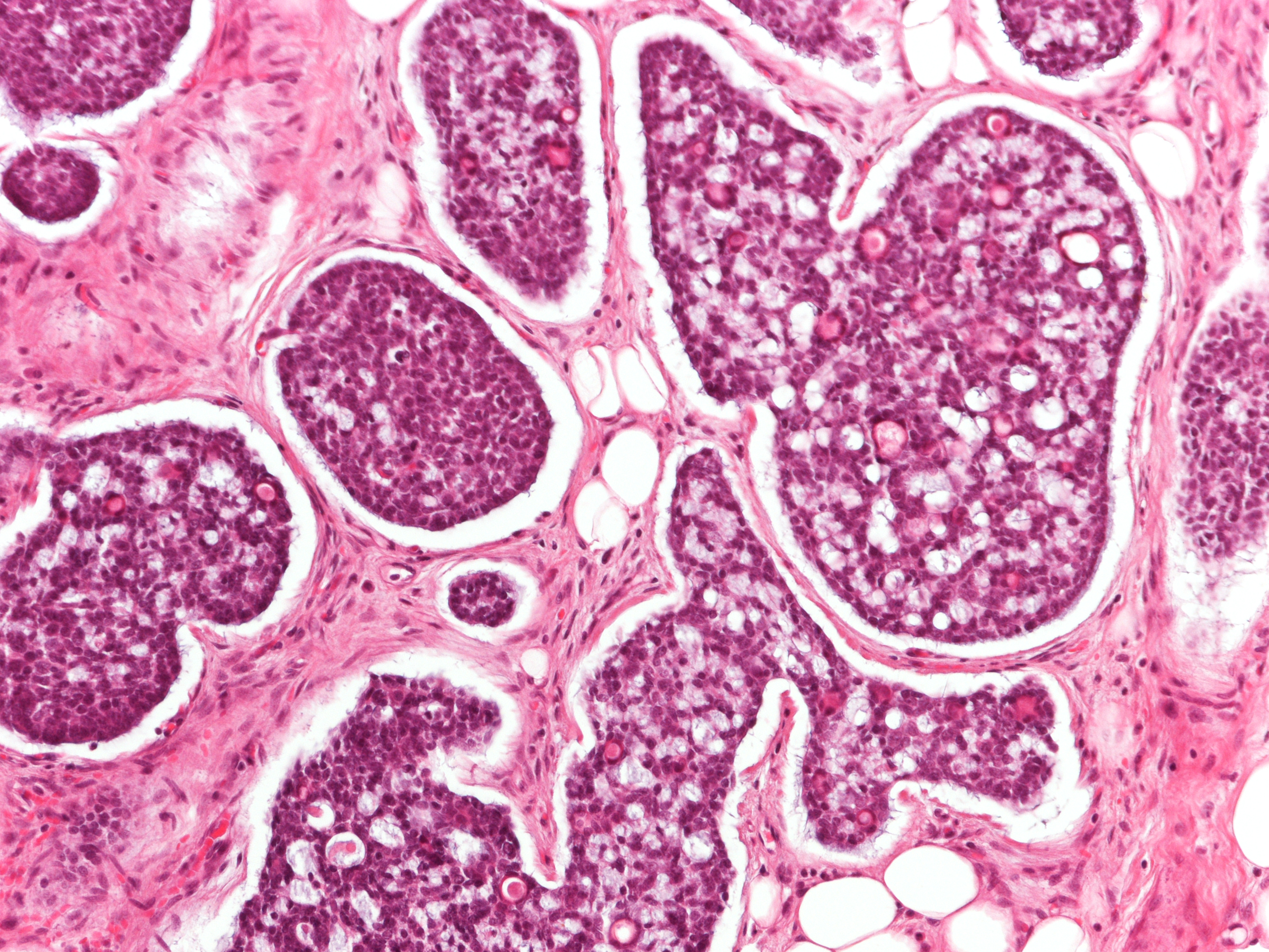

Supplement: Supplementary file 7 — (PNG 9054 kb) [file 428_2021_3174_Fig15_ESM.png]

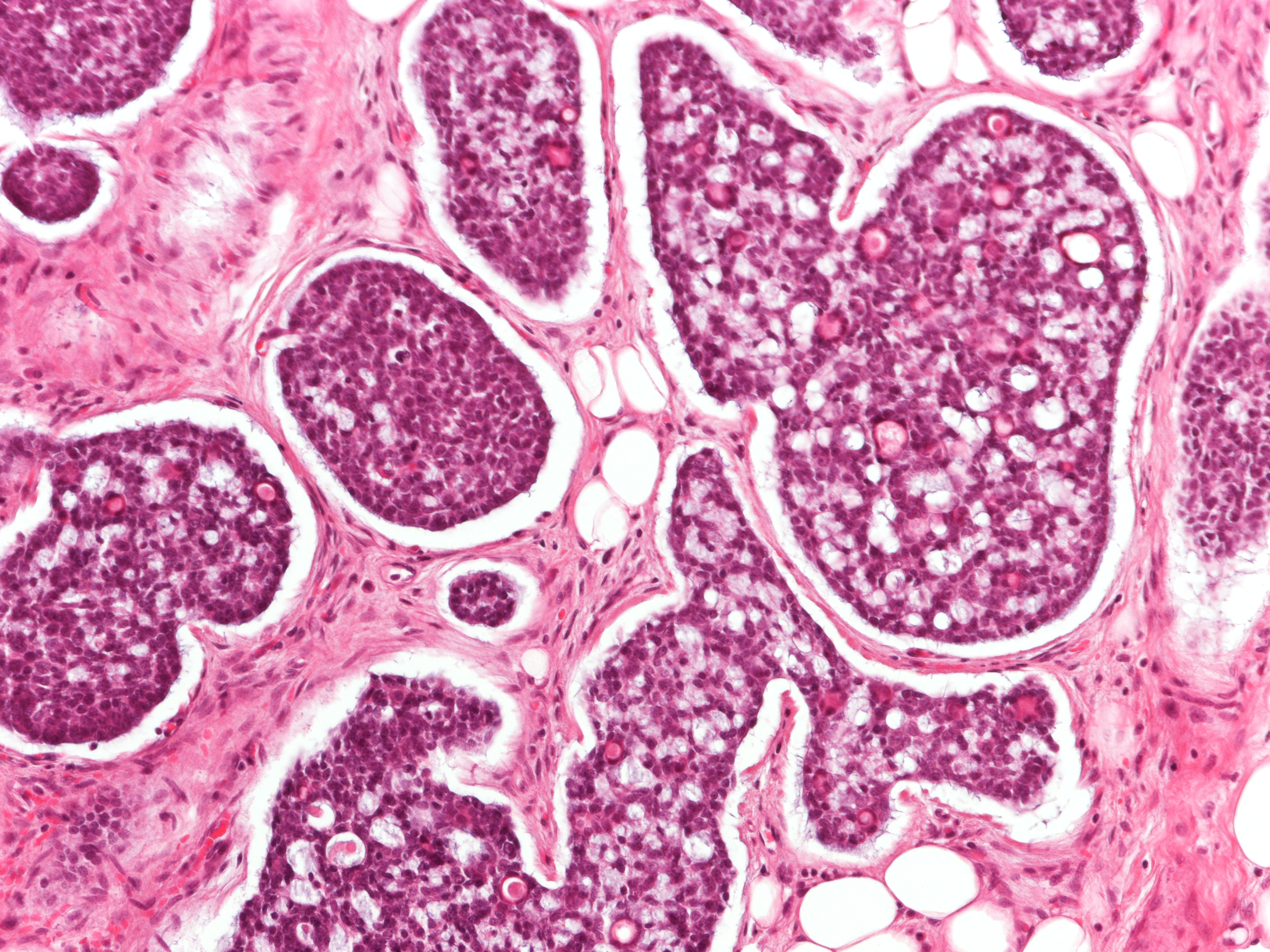

Supplement: Supplementary file 8 — High Resolution Image (TIF 11744 kb) [file 428_2021_3174_MOESM4_ESM.tif]

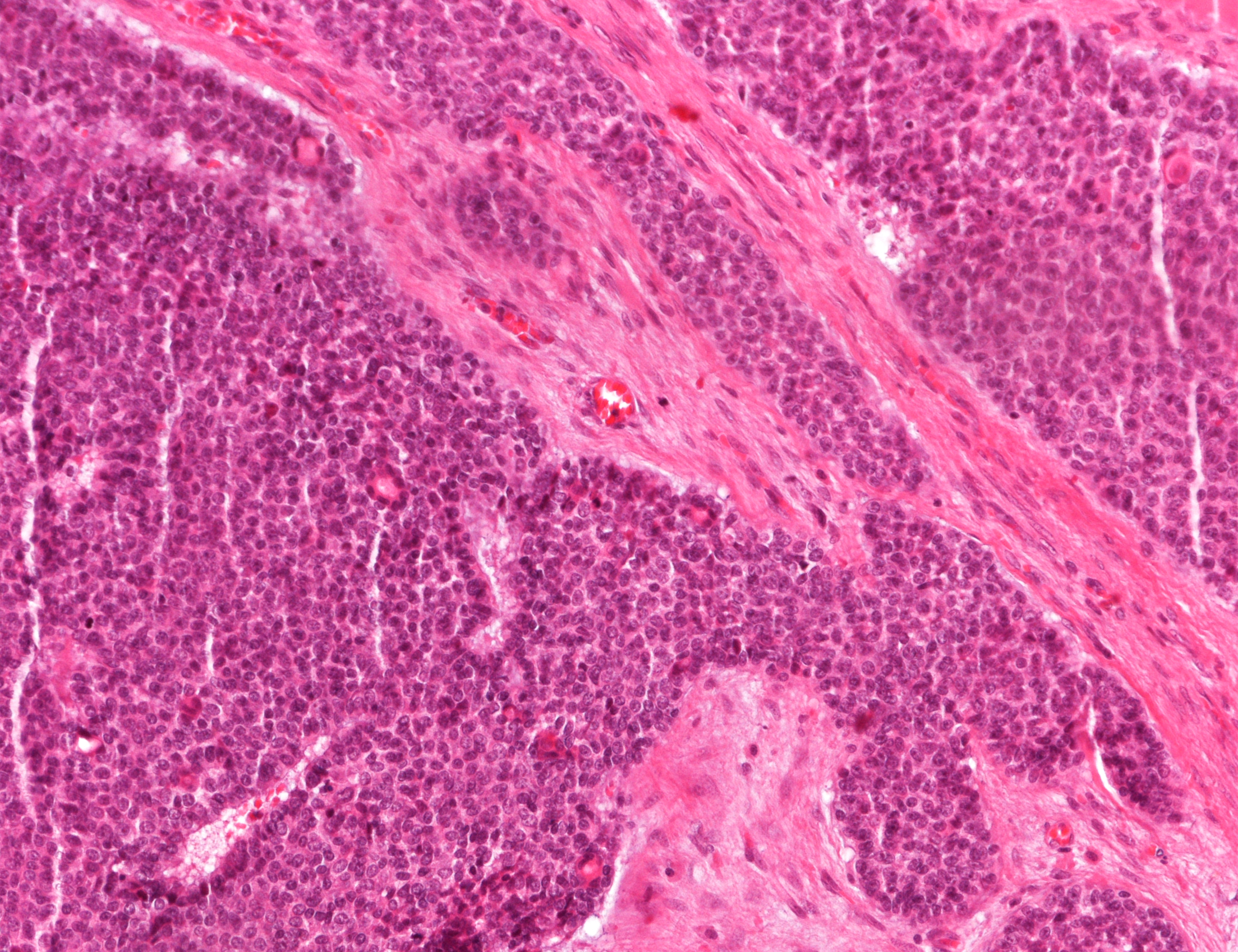

Supplement: Supplementary file 9 — (PNG 7199 kb) [file 428_2021_3174_Fig16_ESM.png]

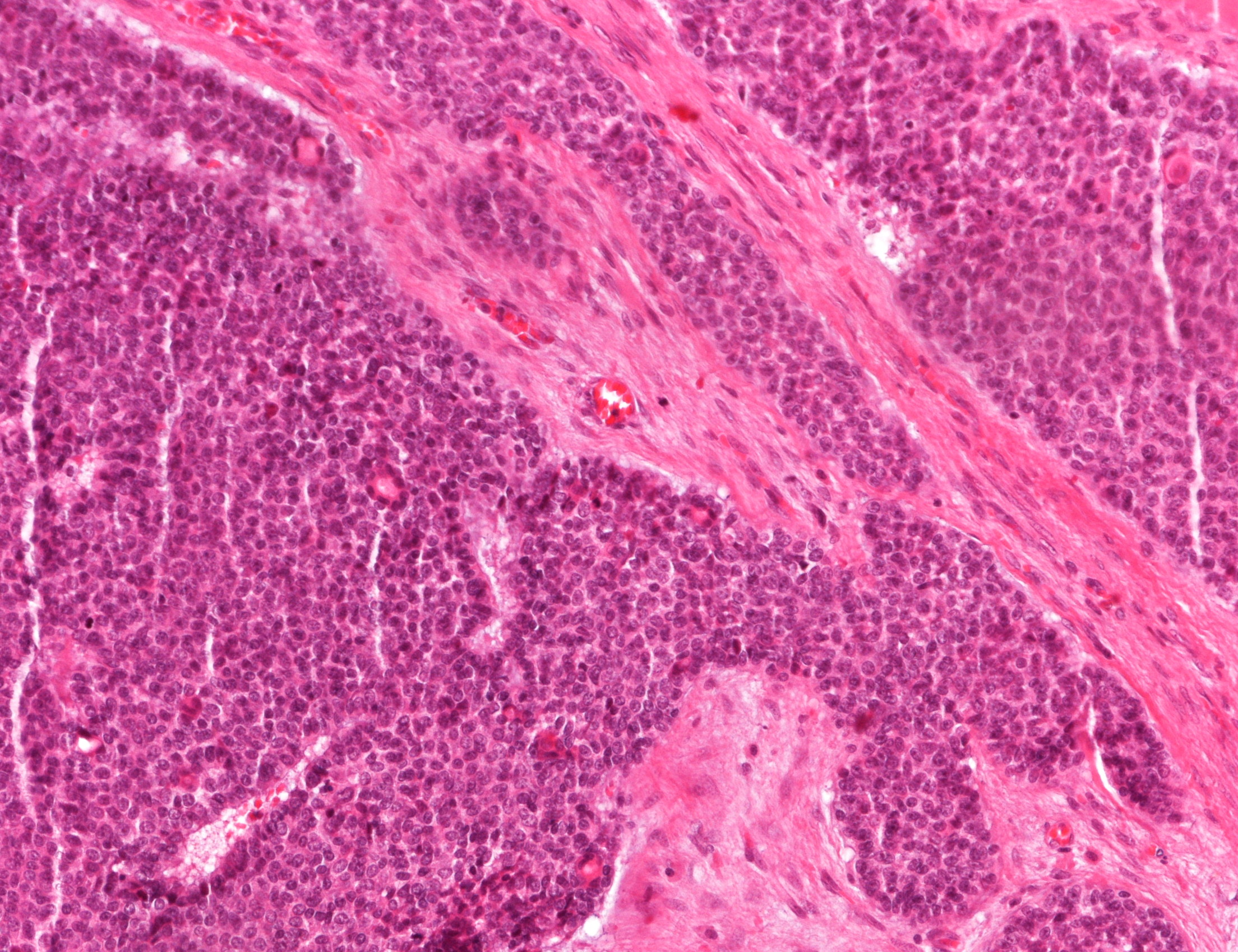

Supplement: Supplementary file 10 — High Resolution Image (TIF 9432 kb) [file 428_2021_3174_MOESM5_ESM.tif]

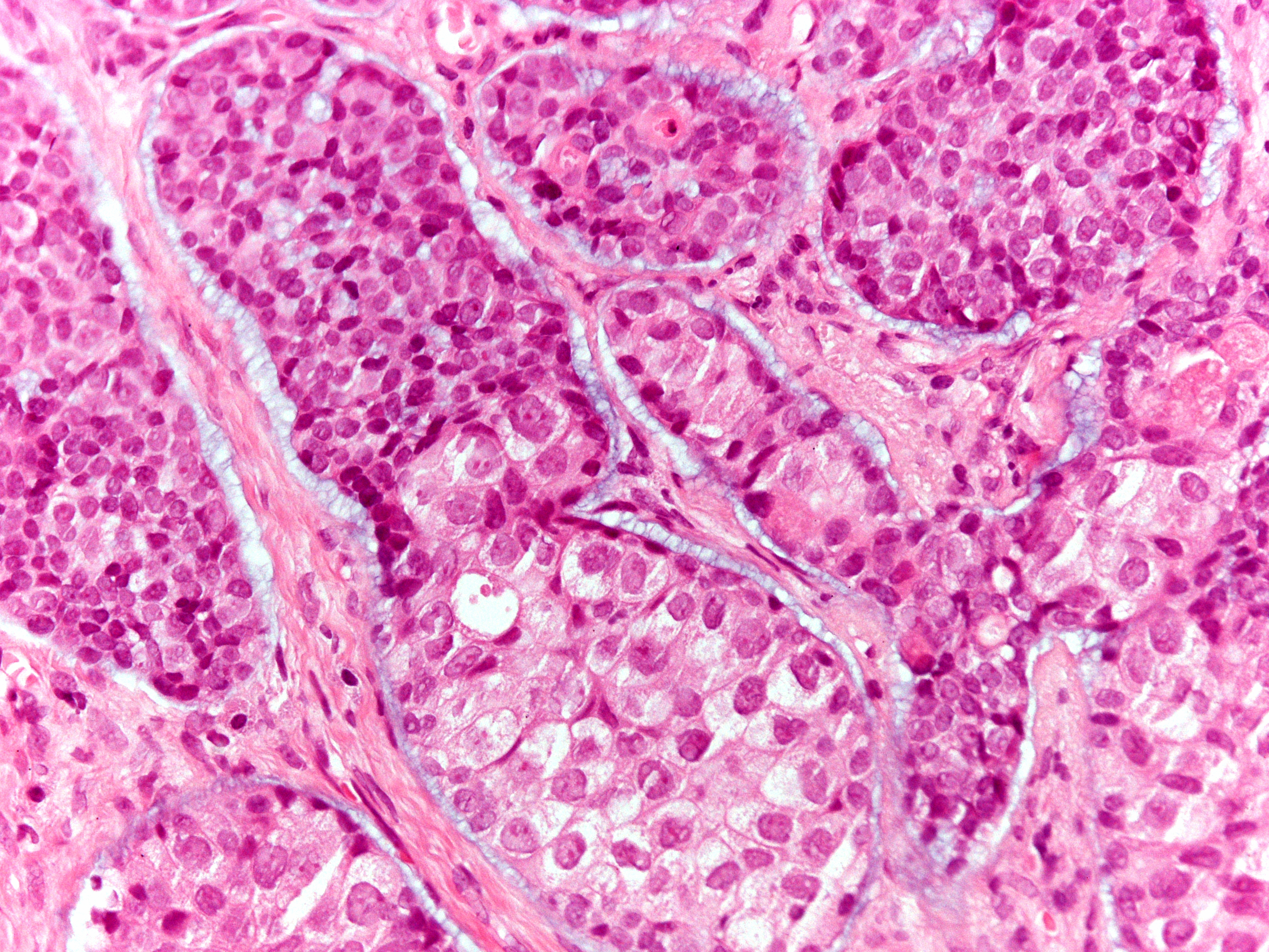

Supplement: Supplementary file 11 — (PNG 10044 kb) [file 428_2021_3174_Fig17_ESM.png]

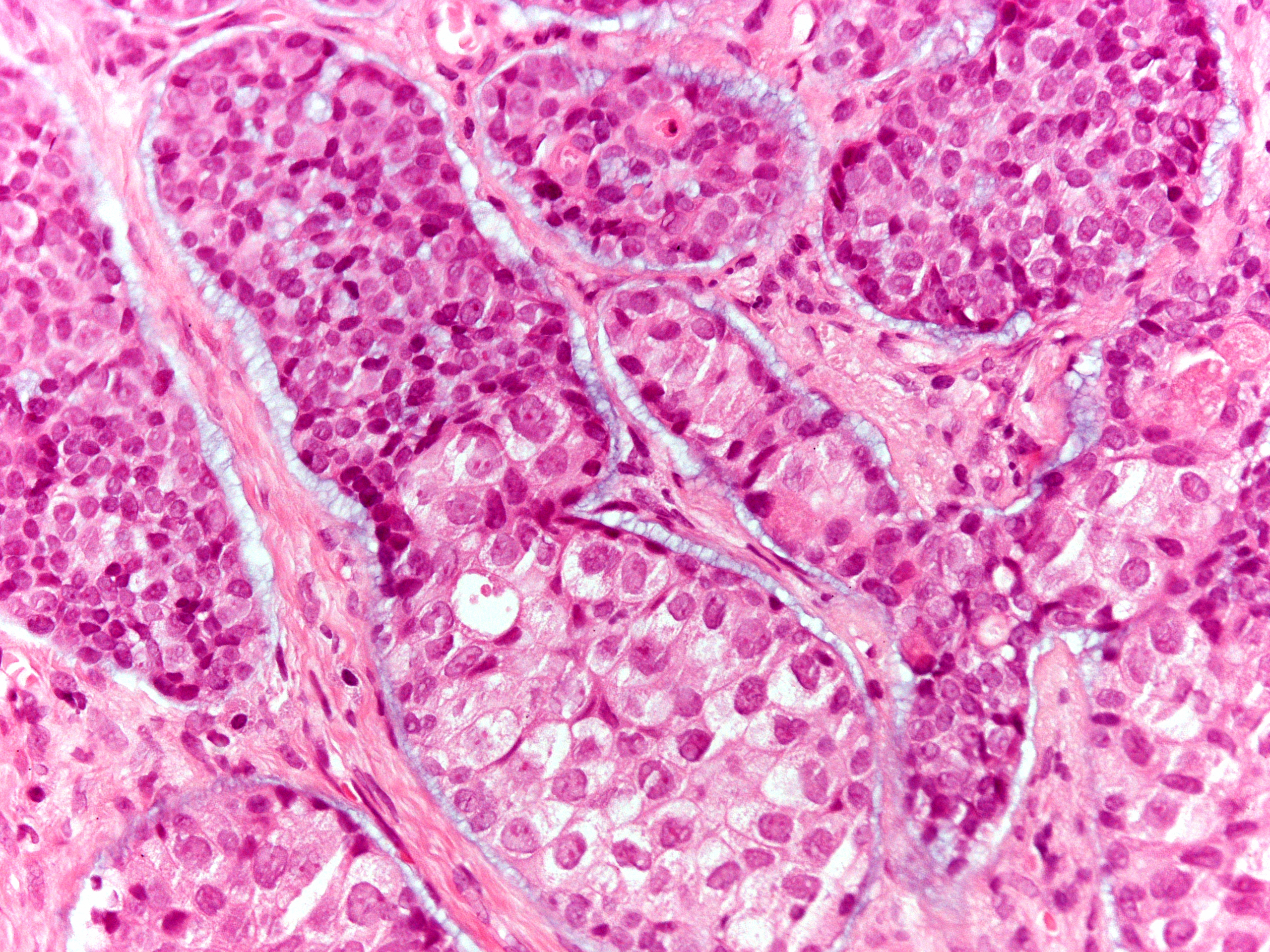

Supplement: Supplementary file 12 — High Resolution Image (TIF 13439 kb) [file 428_2021_3174_MOESM6_ESM.tif]

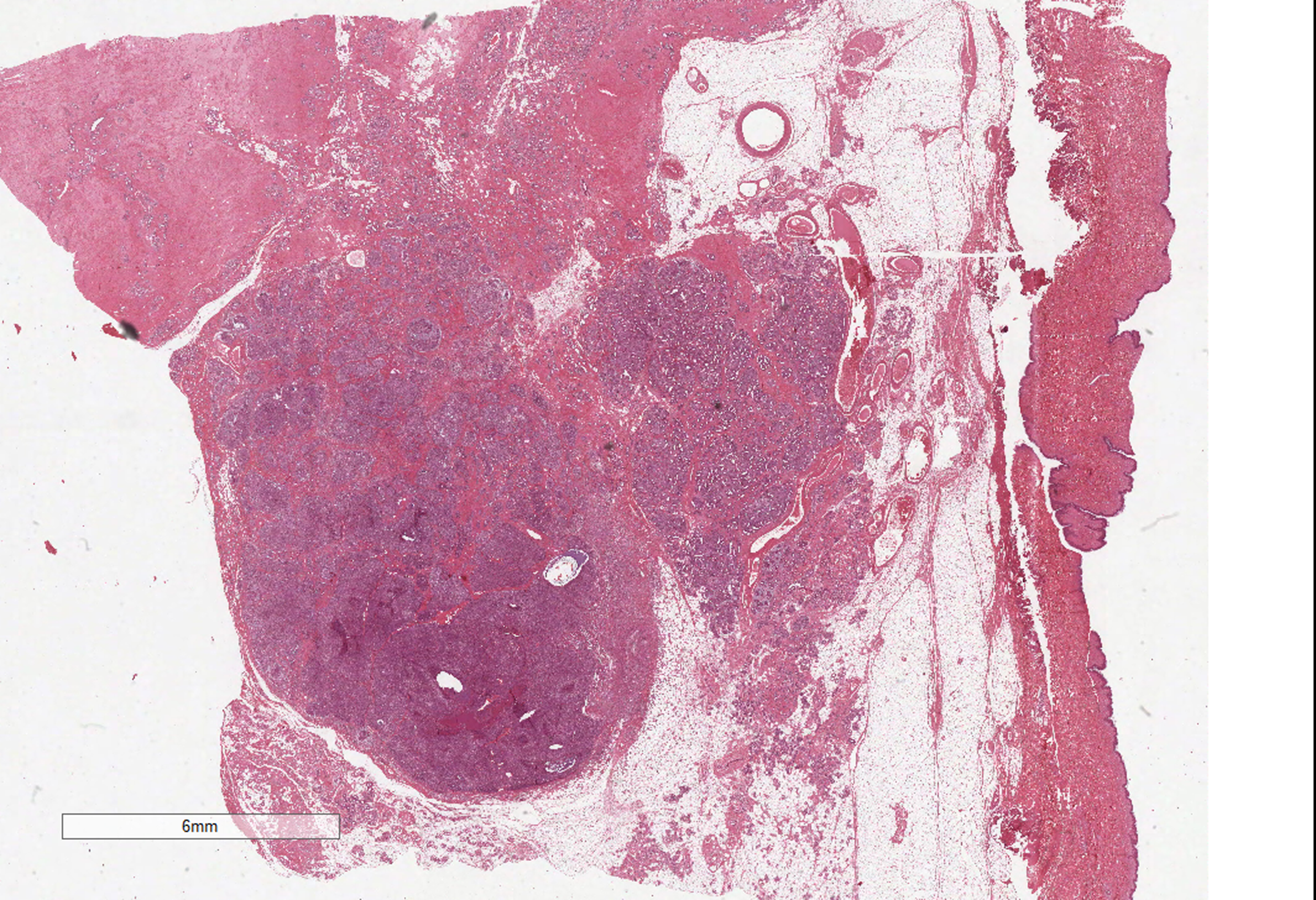

Supplement: Supplementary file 13 — AME showing transition between benign and malignant features. a) At low power the tumour is composed of a central solid component and a peripheral tubular component; b) the peripheral, tubular component shows small glands, lined by an inner epithelial and an outer myoepithelial layer; c, d) atypical areas intermingled with tubular areas; e) the bulk f the tumor is composed of highy atypical cells; a central area of necrosis is present. (PNG 14150 kb) [file 428_2021_3174_Fig18_ESM.png]

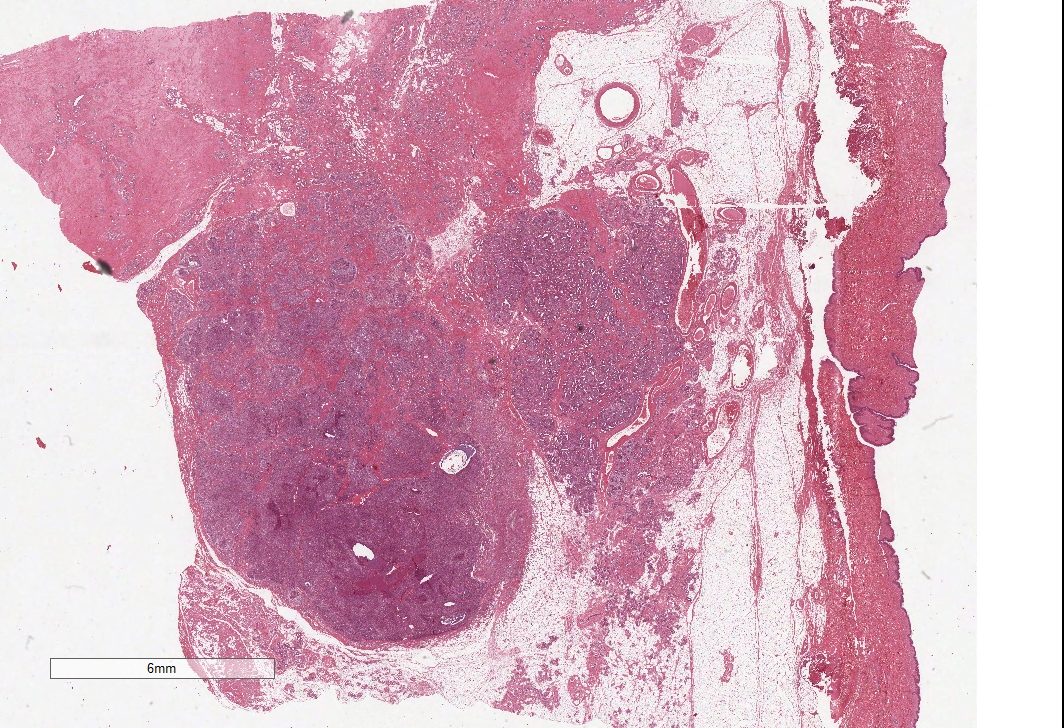

Supplement: Supplementary file 14 — High Resolution Image (TIF 2272 kb) [file 428_2021_3174_MOESM7_ESM.tif]

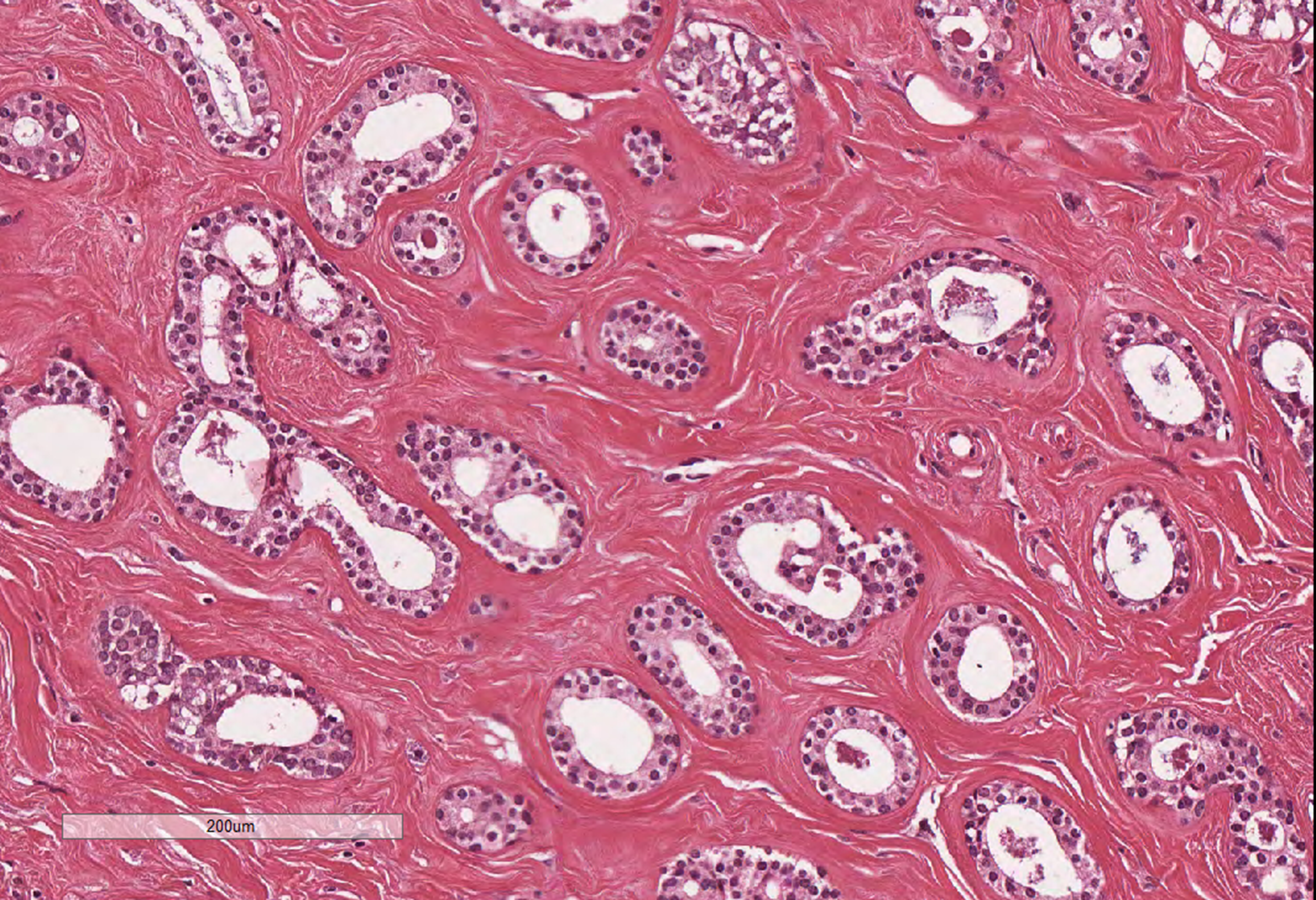

Supplement: Supplementary file 15 — (PNG 14561 kb) [file 428_2021_3174_Fig19_ESM.png]

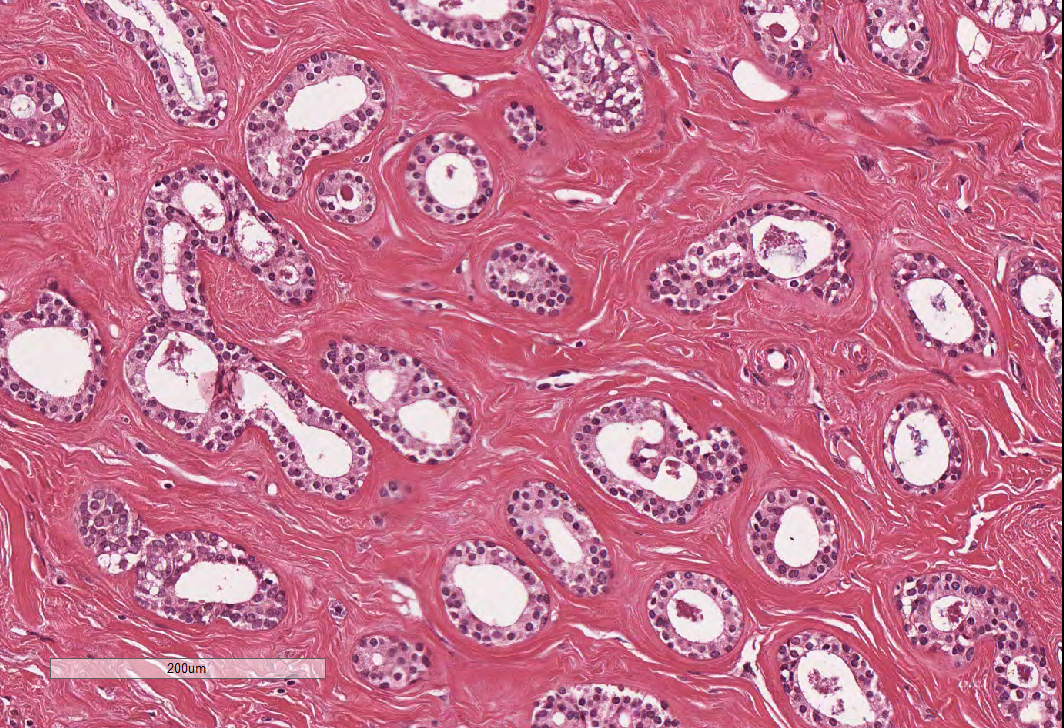

Supplement: Supplementary file 16 — High Resolution Image (TIF 2272 kb) [file 428_2021_3174_MOESM8_ESM.tif]

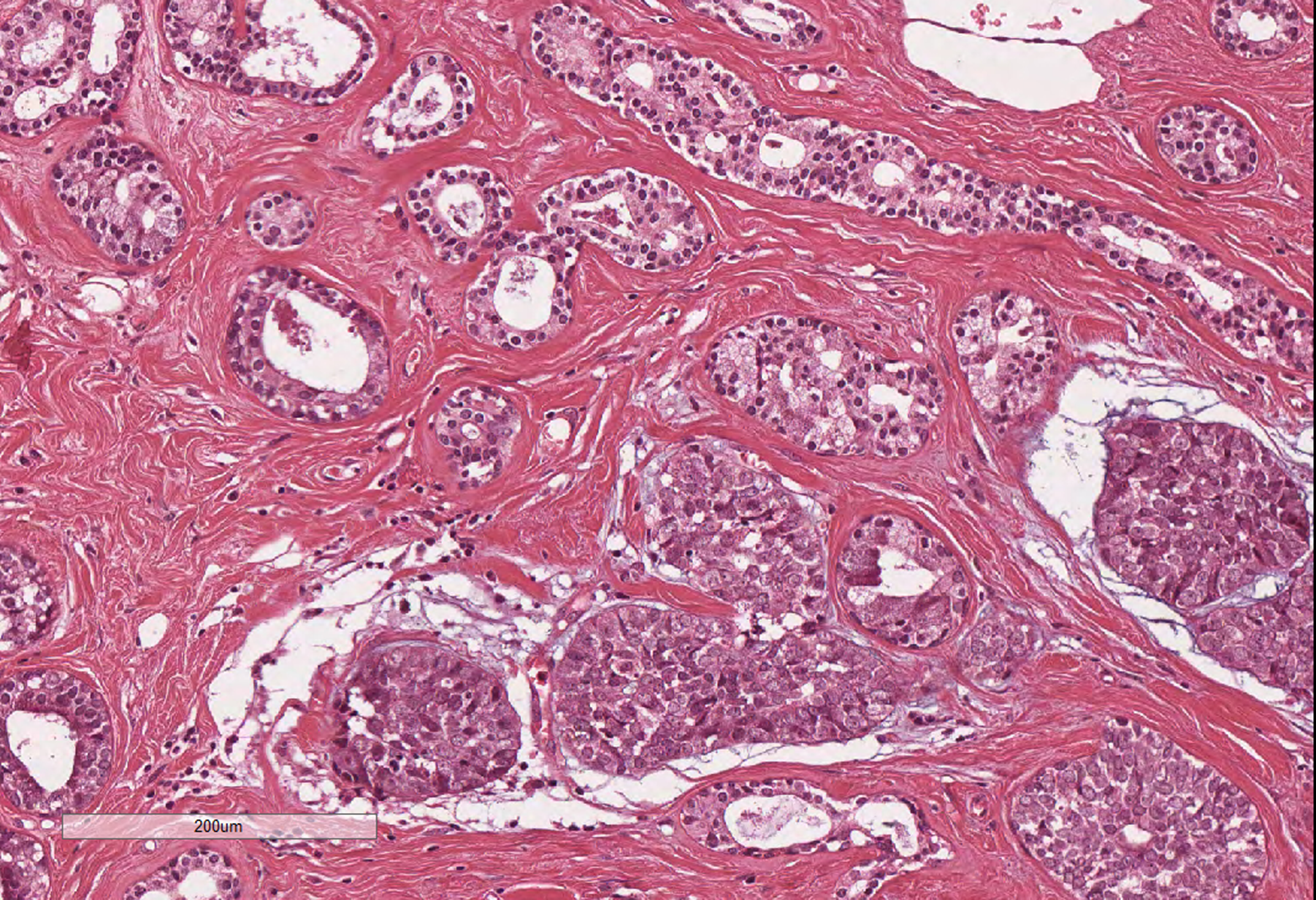

Supplement: Supplementary file 17 — (PNG 15814 kb) [file 428_2021_3174_Fig20_ESM.png]

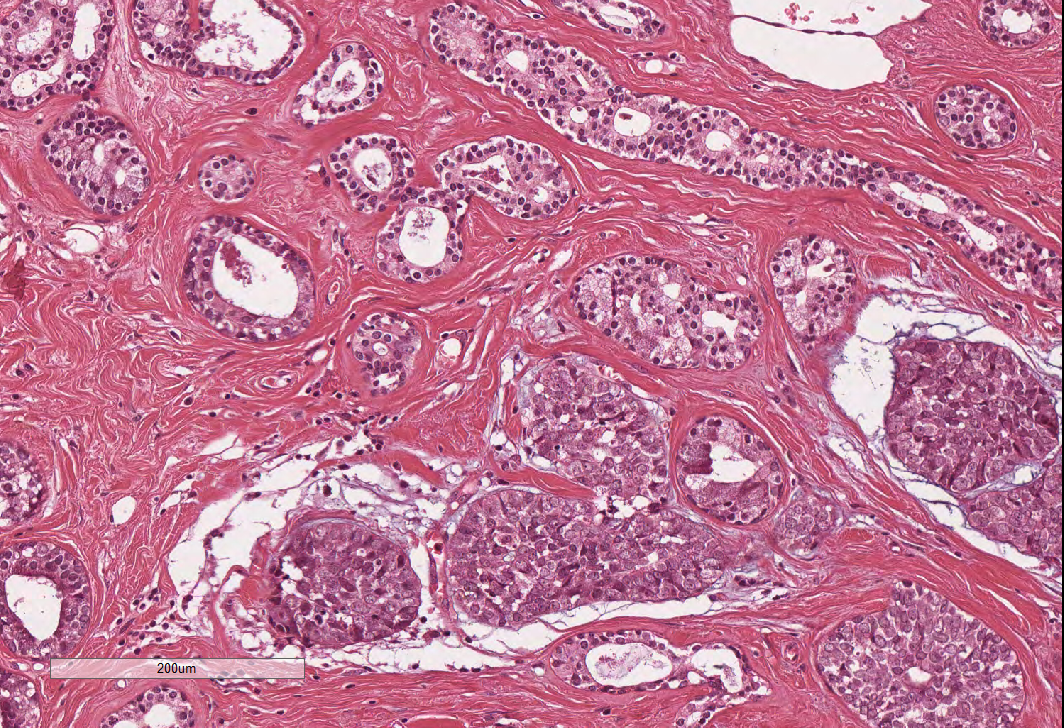

Supplement: Supplementary file 18 — High Resolution Image (TIF 2272 kb) [file 428_2021_3174_MOESM9_ESM.tif]

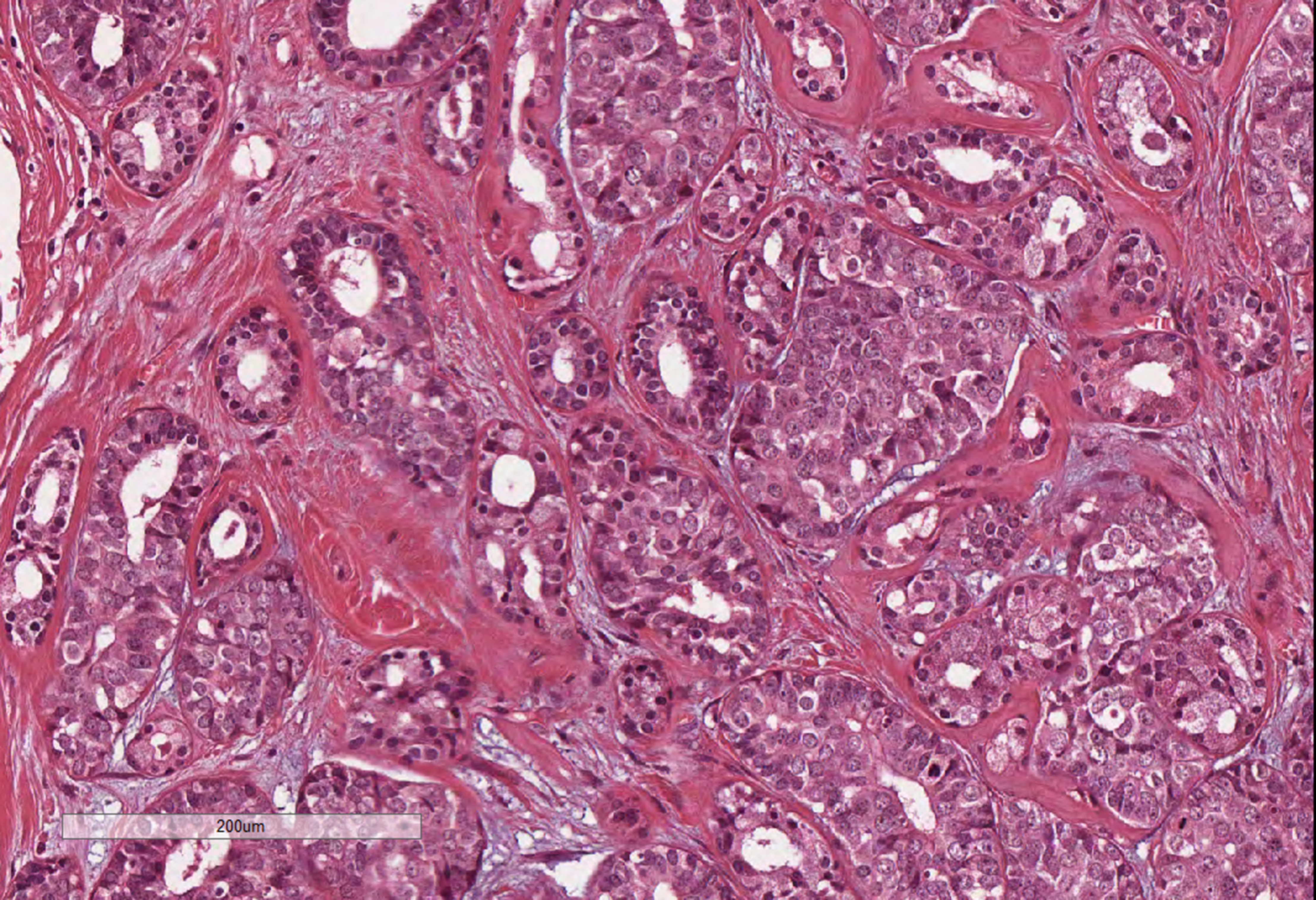

Supplement: Supplementary file 19 — (PNG 15269 kb) [file 428_2021_3174_Fig21_ESM.png]

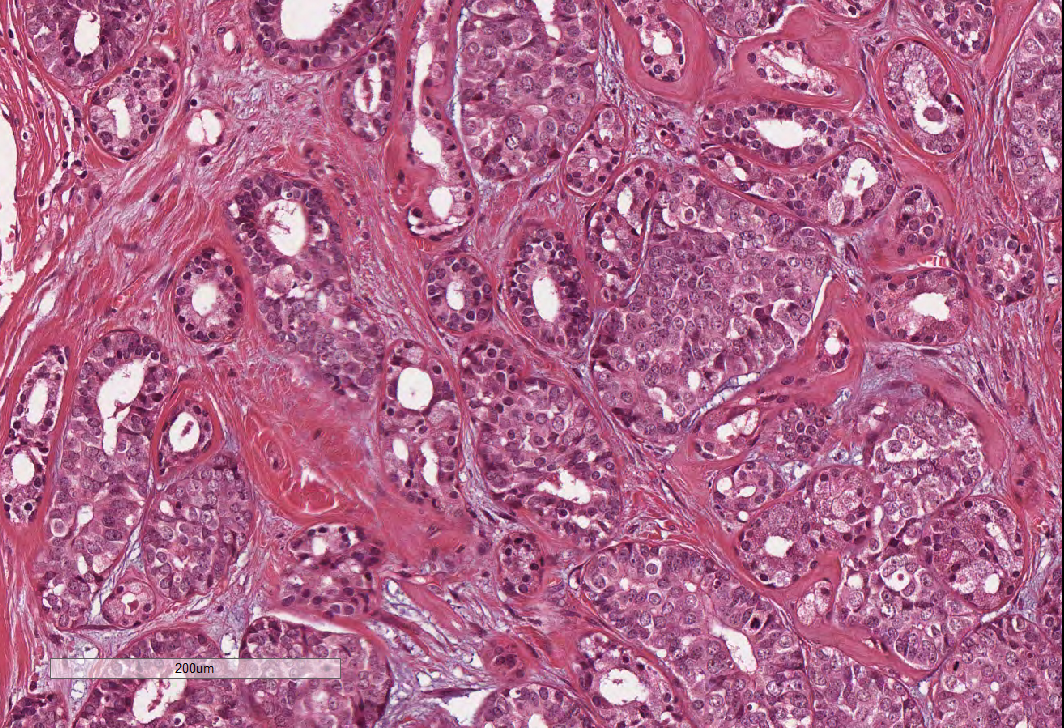

Supplement: Supplementary file 20 — High Resolution Image (TIF 2272 kb) [file 428_2021_3174_MOESM10_ESM.tif]

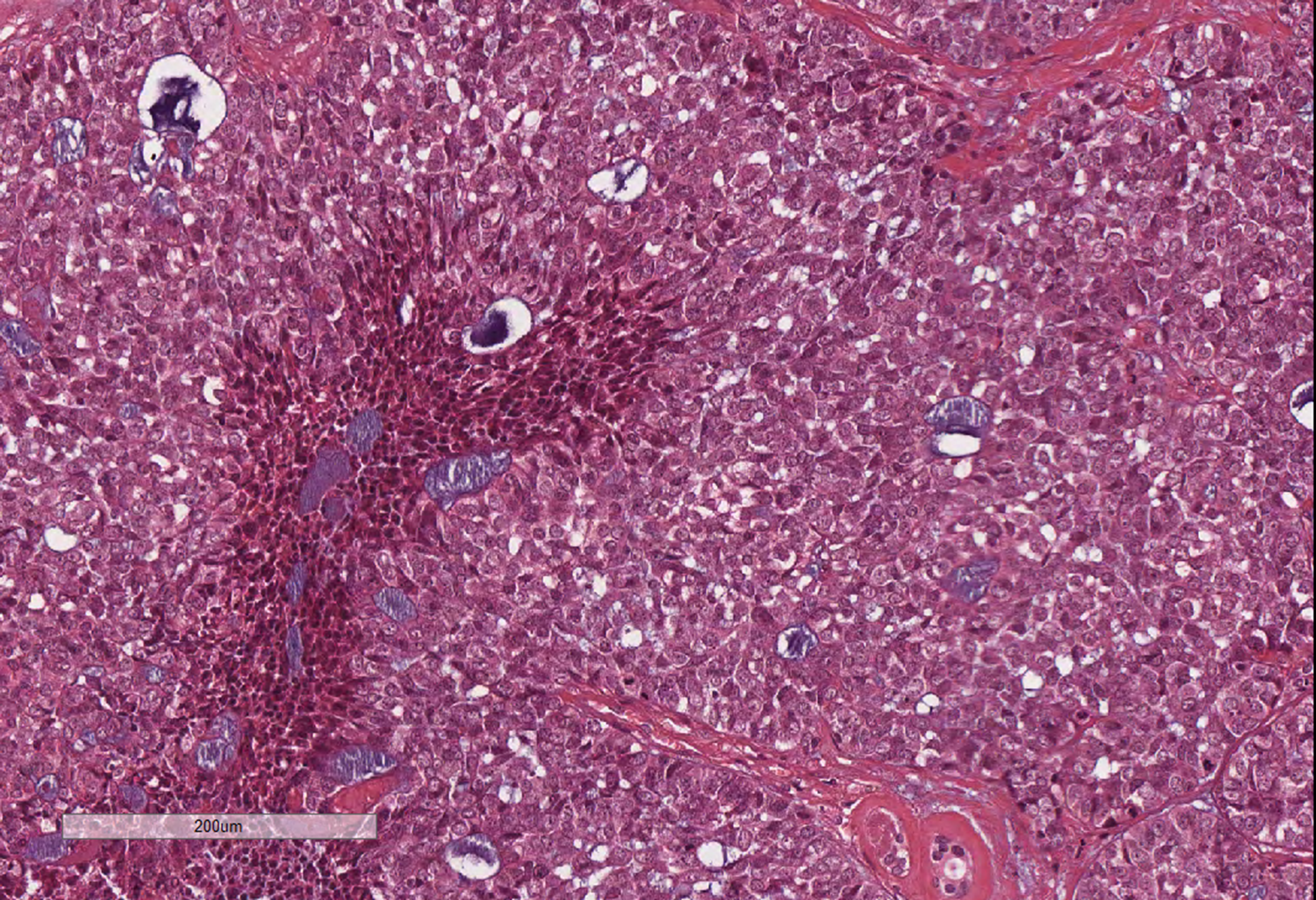

Supplement: Supplementary file 21 — (PNG 16105 kb) [file 428_2021_3174_Fig22_ESM.png]

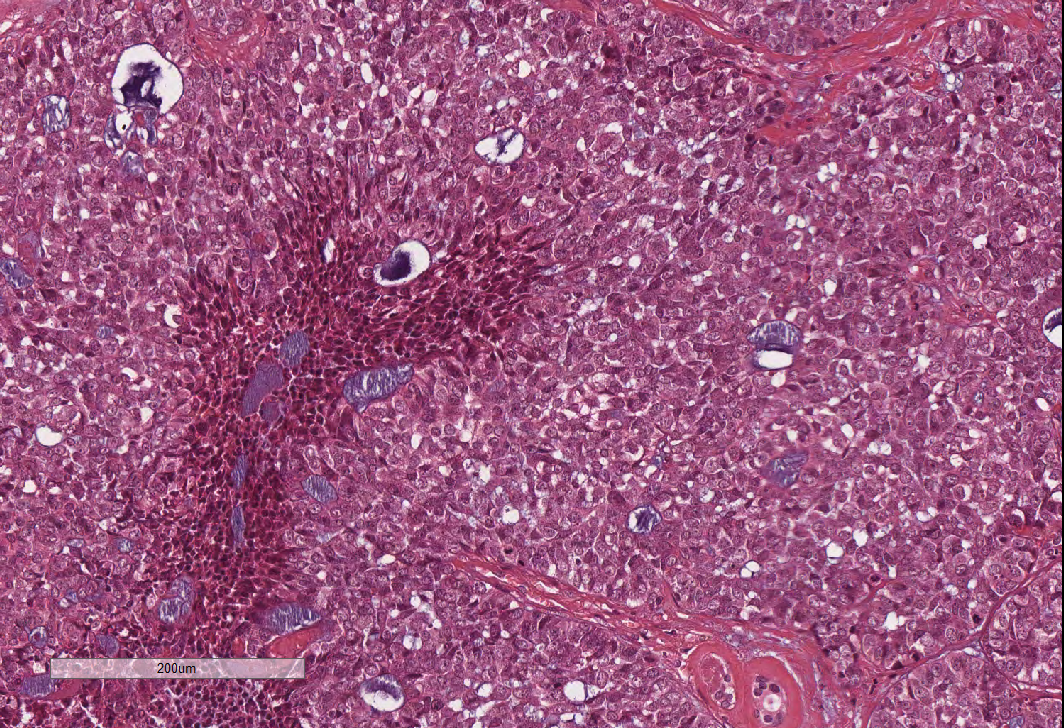

Supplement: Supplementary file 22 — High Resolution Image (TIF 2272 kb) [file 428_2021_3174_MOESM11_ESM.tif]
