# Supplementary material for: Triple-negative breast carcinomas of low malignant potential: review on diagnostic criteria and differential diagnoses
Source: Virchows Arch. 2021 Aug 30;480(1):109–26. doi: 10.1007/s00428-021-03174-7 (PMC8983547; doi:10.1007/s00428-021-03174-7)
Supplement: Supplementary file 23 — (DOC 238 kb) [file 428_2021_3174_MOESM12_ESM.doc]

**Table 1.** Systematic revision of all published cases of adenomyoepithelioma provided of cyto/histological data

| **Authors** | **Year** | **N° of Cases** | **Notes on cases** | **In situ carcinoma** | **Invasive carcinoma** | **Tumor Recurrence/ Metastasis** |
| --- | --- | --- | --- | --- | --- | --- |
| Cameron et al.^1^ | 1974 | 1 | M-AME | No | Yes | Yes / No |
| Zarbo et al.^2^ | 1983 | 1 | C-AME | No | No | No / No |
| Kiaer et al.^3^ | 1984 | 1 | low-grade M-AME in adenomyoepithelial adenosis | No | Yes | Yes / No |
| Eusebi et al.^4^ | 1987 | 2 | C-AME with solid and glandular components | No | No | No / No |
| Young et al.^5^ | 1988 | 3 | 2 C-AME, 1 C-AME in adenomyoepithelial adenosis | No | No | Yes (1) / No |
| Rosen et al.^6^ | 1988 | 18 | C-AME | No | No | Yes (2) / No |
| Jabi et al.^7^ | 1988 | 1 | C-AME | No | No | No / No |
| Tamura et al.^8^ | 1988 | 1 | C-AME | No | No | No / No |
| Weidner et al.^9^ | 1988 | 2 | spindle-cell/tubular C-AME | No | No | No / No |
| Erlandson et al.^10^ | 1989 | 1 | C-AME | No | No | No / No |
| Accurso et al.^11^ | 1990 | 1 | C-AME | No | No | No / No |
| Tavassoli et al.^12^ | 1991 | 28 | 7 spindle-cell C-AME  8 tubular C-AME  10 lobulated C-AME  3 M-AME | No | Yes (3) | Yes (3) / No |
| Saez et al.^13^ | 1992 | 2 | C-AME | No | No | No / No |
| Loose et al.^14^ | 1992 | 6 | 3 tubular C-AME  1 lobulated C-AME  2 M-AME (one with spindle cell sarcoma features) | No | No | Yes (2) / Yes (1)  (lung and brain) |
| Trojani et al.^15^ | 1992 | 1 | M-AME | No | Yes | No / Yes  (lung) |
| Plaza et al.^16^ | 1993 | 2 | C-AME | No | No | No / No |
| Tamura et al.^17^ | 1993 | 1 | spindle-cell C-AME in a male | No | No | No / No |
| Vielh et al.^18^ | 1993 | 1 | C-AME with predominant solid component | No | No | No / No |
| Birdsong et al.^19^ | 1993 | 1 | C-AME | No | No | No / No |
| Michal et al.^20^ | 1994 | 1 | M-AME with undifferentiated carcinoma | No | Yes | Yes / No |
| Chen et al.^21^ | 1994 | 1 | M-AME | No | Yes | No / Yes (widespread) |
| Pauwels et al.^22^ | 1994 | 1 | M-AME | No | Yes | Yes / No |
| Diomandè et al.^23^ | 1994 | 1 | C-AME | No | No | No / No |
| Valente et al.^24^ | 1994 | 1 | C-AME | No | No | No / No |
| Nilsson et al.^25^ | 1994 | 1 | C-AME | No | No | No / No |
| Hock et al.^26^ | 1994 | 1 | C-AME | No | No | No / No |
| Niemann et al.^27^ | 1995 | 1 | A-AME | No | No | No / No |
| Foschini et al.^28^ | 1995 | 6 | 3 C-AME  3 M-AME with low-grade ASC carcinoma | No | Yes (3) | Yes (1) / Yes (1, lung) |
| Khattech et al.^29^ | 1995 | 1 | C-AME | No | No | No / No |
| Meunier et al.^30^ | 1995 | 1 | C-AME | No | No | No / No |
| Choi et al.^31^ | 1996 | 3 | 1 tubular C-AME  2 lobulated C-AME with fibroadenomatous areas | No | No | No / No |
| Nomura et al.^32^ | 1996 | 1 | A-AME | No | No | No / No |
| Park et al.^33^ | 1996 | 1 | C-AME | No | No | No / No |
| Pogacnik et al.^34^ | 1996 | 1 | C-AME | No | No | No / No |
| McGluggage et al.^35^ | 1997 | 2 | C-AME | No | No | No / No |
| Van Dorpe et al.^36^ | 1998 | 1 | C-AME with ACC | No | Yes | No / No |
| Laforga et al.^37^ | 1998 | 2 | C-AME with prominent cystic changes and intranuclear inclusions | No | No | No / No |
| Gupta et al.^38^ | 1998 | 1 | C-AME | No | No | No 7 No |
| Rasbridge et al.^39^ | 1998 | 7 | 4 M-AME  3 A-AME | No | Yes (4) | Yes (5) / Yes (1,  (cerebral cortex) |
| Simpson et al.^40^ | 1998 | 1 | M-AME with mixed osteogenic, spindle cell and carcinomatous differentiation | No | Yes | Yes , Yes  (lung) |
| Takahashi et al.^41^ | 1999 | 1 | M-AME | No | Yes | Yes (bone, lung, lymph node) |
| Singh Gill et al.^42^ | 2000 | 2 | C-AME | No | No | No / No |
| Ahmed et al.^43^ | 2000 | 1 | M-AME | No | Yes | No / No |
| Bult et al.^44^ | 2000 | 1 | M-AME | No | Yes | No / Yes  (thyroid) |
| Lee et al.^45^ | 2000 | 1 | C-AME indistinguishable from PT on cytology | No | No | No / No |
| Rammeh-Rommani et al.^46^ | 2000 | 2 | 1 C-AME  1 A-AME | No | No | No / No |
| Tukel et al.^47^ | 2001 | 1 | C-AME | No | No | No / No |
| Aydin et al.^48^ | 2001 | 1 | C-AME | No | No | No / No |
| Kihara et al.^49^ | 2001 | 1 | M-AME | No | Yes | No / Yes  (lung) |
| Sugano et al.^50^ | 2001 | 2 | M-AME  myxoid/chondroid matrix (both cases), cartilage/mature bone (one case) | No | Yes | Yes (1) / No |
| Viguer et al.^51^ | 2001 | 1 | tubular C-AME | No | No | No / No |
| Chang et al.^52^ | 2002 | 1 | C-AME | No | No | No / No |
| Felipo et al.^53^ | 2002 | 1 | C-AME grossly mimicking a malignancy | No | No | No / No |
| Ng^54^ | 2002 | 3 | 1 C-AME, 2 M-AME with focal carcinomatous transformation | No | Yes (2) | No / No |
| Kinkor et al.^55^ | 2002 | 1 | M-AME | No | Yes | No / No |
| Kurashina^56^ | 2002 | 2 | 1 tubular C-AME  1 M-AME | No | Yes (1) | No / No |
| Popnikolov et al.^57^ | 2003 | 8 | C-AME | No | No | No / No |
| Howlett et al.^58^ | 2003 | 3 | 1 C-AME  1 C-AME with IBC-NST  1 M-AME with high-grade myoepithelial carcinoma | No | Yes (2) | No / No |
| Jones et al.^59^ | 2003 | 1 | M-AME | No | Yes | No / Yes  (liver) |
| Zhang et al.^60^ | 2004 | 1 | A-AME | No | No | No / No |
| Reis-Filho et al.^61^ | 2004 | 1 | C-AME with CS | No | No | No / No |
| Harigopal et al.^62^ | 2004 | 1 | M-AME | No | Yes | No / No |
| Papaevangelou et al.^63^ | 2004 | 1 | Cystic C-AME | No | No | No / No |
| Mathur et al.^64^ | 2004 | 1 | C-AME | No | No | No / No |
| Gatti et al.^65^ | 2004 | 1 | C-AME presenting as a cancer | No | No | No / No |
| Loh et al.^66^ | 2004 | 1 | C-AME | No | No | Yes / No |
| McLaren et al.^67^ | 2005 | 23 | 4 lobulated C-AME  19 encysted C-AME | No | No | No / No |
| Cai et al.^68^ | 2005 | 1 | C-AME with squamous and sebaceous metaplasia | No | No | No / No |
| Salto-Tellez et al.^69^ | 2005 | 5 | 1 C-AME with microsatellite instability for BRCA1 and loss of heterozygosity of HPC1  4 C-AME without genetic alterations | No | No | No / No |
| Gatalica et al.^70^ | 2005 | 1 | C-AME with t (8; 16) (p23; q21) | No | No | No / No |
| Okada et al.^71^ | 2005 | 1 | C-AME with loss of E-cadherin | No | No | No / No |
| Tait et. Al.^72^ | 2005 | 1 | C-AME | No | No | No / No |
| Nadelman et al.^73^ | 2006 | 2 | Metastasizing C-AME | No | No | Yes (1), Yes (2,  lung) |
| Han et al.^74^ | 2006 | 1 | M-AME with point mutation of P53 gene | No | Yes | No / No |
| Buch et al.^75^ | 2006 | 1 | C-AME with PT | No | Yes | No / No |
| Noel et al.^76^ | 2006 | 1 | M-AME | No | Yes | No / No |
| Hikino et al.^77^ | 2007 | 1 | intracystic C-AME | No | No | No / No |
| Oka et al.^78^ | 2007 | 1 | M-AME with matrix production | No | Yes | No / No |
| Fan et al.^79^ | 2007 | 1 | M-AME | No | Yes | No / No |
| Mercado at al.^80^ | 2007 | 4 | C-AME | No | No | No / No |
| Ruiz-Delgado at al.^81^ | 2007 | 1 | C-AME | No | No | No / No |
| Huang et al.^82^ | 2007 | 1 | C-AME | No | No | No / No |
| Catena et al.^83^ | 2008 | 1 | C-AME | No | No | No / No |
| Yahara et al.^84^ | 2008 | 1 | C-AME | No | No | No / No |
| Kuroda et al.^85^ | 2008 | 1 | C-AME with coexistent IBC-NST | No | Yes | No / No |
| Samanta et al.^86^ | 2009 | 1 | Metastasizing C-AME | No | No | No / Yes  (lung) |
| Qureshi et al.^87^ | 2009 | 1 | M-AME | No | Yes | No / No |
| Honda et al.^88^ | 2009 | 1 | M-AME combined with ILC | No | Yes | Yes  (lung, kidneys) |
| Hegyi et al.^89^ | 2009 | 1 | M-AME | No | Yes | No / No |
| Zizi-Sermpetzoglou et al.^90^ | 2009 | 1 | M-AME | No | Yes | No / No |
| Hayes et al.^91^ | 2010 | 25 | 12 C-AME  13 M-AME | No | Yes (13) | No / Yes (4)  (not specified) |
| Buza et al.^92^ | 2010 | 3 | 3 M-AME (1 case with DCIS) | Yes (1) | Yes | No / No |
| Ohta et al.^93^ | 2010 | 1 | C-AME with CS | No | No | No / No |
| Han et al.^94^ | 2010 | 1 | multicentric A-AME with DCIS | Yes | No | No / No |
| Khurana et al.^95^ | 2010 | 1 | M-AME | No | Yes | No / No |
| Ghandi et al.^96^ | 2011 | 1 | M-AME | No | Yes | No / No |
| Adejolu et al.^97^ | 2011 | 1 | C-AME | No | No | No / No |
| Matsumoto et al.^98^ | 2011 | 1 | C-AME | No | No | No / No |
| Saad et al.^99^ | 2012 | 1 | A-AME | No | No | No / No |
| Awamleh et al.^100^ | 2012 | 1 | M-AME | No | Yes | No / Yes  (lymph node) |
| Maffini et al.^101^ | 2013 | 1 | M-AME | No | Yes | No / Yes  (lung) |
| Bajpai et al.^102^ | 2013 | 1 | bilateral C-AME | No | No | No / No |
| Petrozza et al.^103^ | 2013 | 1 | M-AME | No | Yes | No / No |
| Marian et al.^104^ | 2013 | 2 | M-AME | No | Yes | No / No |
| Warrier et al.^105^ | 2013 | 1 | C-AME with MGA-like pattern and DCIS | Yes | No | No / No |
| Wang et al.^106^ | 2013 | 1 | M-AME | No | Yes | Yes  (lymph node) |
| Yang et al.^107^ | 2014 | 1 | M-AME with ACC | No | Yes | No / No |
| Kalyani et al.^108^ | 2014 | 1 | M-AME | No | Yes | No / Yes  (bone) |
| Robinson et al.^109^ | 2014 | 1 | M-AME | No | Yes | No / No |
| Wakasa et al.^110^ | 2014 | 1 | C-AME | No | No | No / No |
| Kamei et al.^111^ | 2015 | 1 | C-AME with DCIS | Yes | No | No / No |
| Zhang et al.^112^ | 2015 | 1 | C-AME with ALH | No | No | No / No |
| Delteil et al.^113^ | 2015 | 1 | C-AME with dominant myoepithelial component | No | No | No / No |
| An Gkali et al.^114^ | 2015 | 1 | lobulated C-AME | No | No | No / No |
| Zhu et al.^115^ | 2015 | 1 | lobulated C-AME | No | No | No / No |
| Ahmadi et al.^116^ | 2015 | 1 | M-AME | No | Yes | No / No |
| Lee et al.^117^ | 2015 | 1 | M-AME responsive to Erbulin | No | Yes | No / Yes  (hepatic, pleural, abdominal) |
| Moritani et al.^118^ | 2015 | 16 | 4 spindle-cell C-AME  9 clear-cell C-AME  3 mixed C-AME | No | No | No / No |
| Korolczuk et al.^119^ | 2016 | 1 | M-AME | No | Yes | No / Yes  (lung) |
| Moritz et al.^120^ | 2016 | 14 | 10 C-AME (5 tubular, 3 spindle-cell, 2 lobulated)  1 C-AME with DCIS  2 C-AME with synchronous IBC-NST  1 M-AME with synchronous malignant PT | Yes (1) | Yes (1) | No / No |
| Xu et al.^121^ | 2017 | 2 | M-AME | No | Yes | No / No |
| Smith Iorfido et al.^122^ | 2017 | 1 | bifocal C-AME | No | No | No / No |
| Jones et al.^123^ | 2017 | 1 | M- AME | No | Yes | No / No |
| Logie et al.^124^ | 2017 | 1 | M-AME | No | Yes | No / Yes  (lymph node) |
| Yuan et al.^125^ | 2017 | 2 | M-AME | No | Yes | No / No |
| Jassar et al.^126^ | 2017 | 1 | tubular C-AME | No | No | No / No |
| Baraban et al.^127^ | 2018 | 11 | 5 C-AME  2 A-AME  4 M-AME | No | Yes (4) | No / No |
| Geyer et al.^128^  Pareja ^129^  Pereja ^130^ | 2018  2019  2020 | 43*  13*  26* | 25 C-AME,  18 A-AME  52% PIK3CA mutations  26% HRAS mutations  13% AKT1 mutations  13% PIK3R1 mutations  1 case with PLAG1/HMGA2 rearrangement  71% of AME with HRAS Q61R mutation were positive for RAS Q61R at IHC | No | No | No / No |
| Del Arco et al.^131^ | 2018 | 4 | C-AME | No | No | No / No |
| Antonelli et al.^132^ | 2018 | 1 | M-AME | No | Yes | No / No |
| Ito et al.^133^ | 2019 | 1 | M-AME | No | Yes | No / No |
| Watanabe et al.^134^ | 2019 | 1 | M-AME with codon-61 HRAS mutation | No | Yes | Yes / Yes (lung) |
| Kakkar et al.^135^ | 2019 | 1 | M-AME | No | Yes | No / No |
| Kim et al.^136^ | 2019 | 1 | M-AME | No | Yes | No / No |
| Hempenstall et al.^137^ | 2019 | 1 | M-AME with multifocal ASC | No | Yes | No / No |
| Zhuo et al.^138^ | 2019 | 1 | C-AME wit ACC | No | Yes | No / No |
| Gafton et al.^139^ | 2019 | 1 | M-AME | No | Yes | No / No |
| Fabres-Aldana et al.^140^ | 2020 | 1 | M-AME with c-Myc amplification | No | Yes | No / No |
| Ginter et al.^141^ | 2020 | 7 | 1 AME, 2 A-AME, 1 MIS-AME,  3 M-AME  PIK3CA mutation (A-AME, MIS- AME)  EGFR amplification (MIS-AME)  AKT1 mutation (C-AME, M--AME)  GNAS mutation (C-AME)  co-occurring HRAS-PIK3CA mutations (1 A-AME) | Yes (1) | Yes (3) | No / Yes (1, lung) |
| Wiens et al.^142^ | 2020 | 12 | 6 C-AME  3 A-AME  3 M-AME | No | Yes (3) | No / No |
| Lari et al.^143^ | 2020 | 1 | M-AME | No | Yes | No / No |
| Moro et al.^144^ | 2020 | 1 | M-AME | No | Yes | Yes / Yes  (lung, brain, heart, kidney) |
| Intagliata et al.^145^ | 2020 | 2 | C-AME | No | No | No / No |
| Harada et al.^146^ | 2021 | 1 | C-AME with PA-like component in a male | No | No | No / No |
| Amano et al.^147^ | 2021 | 1 | C-AME with HER2 expression/amplification and DCIS | Yes (1) | No | No / No |
| El-Helou et al.^148^ | 2021 | 1 | C-AME | No | No | No / No |
| Zhang et al.^149^ | 2021 | 1 | M-AME | No | Yes (1) | No / No |

Abbreviations: N°: number; M-AME: malignant adenomyoepithelioma; C-AME: classical adenomyoepithelioma; ASC: adenosquamous carcinoma; ACC: adenoid-cystic carcinoma; A-AME: atypical adenomyoepithelioma; PT: phyllodes tumor; IBC-NST: invasive breast cancer of no special type; CS: collagenous spherulosis; ILC: invasive lobular carcinoma; DCIS: ductal carcinoma in situ;  MGA: microglandular adenosis; ALH: atypical lobular hyperplasia; MIS-AME: malignant in situ adenomyoepithelioma; PA: pleomorphic adenoma

*****belong to the same case series (Geyer et al. 2018)
