# Supplementary material for: Triple-negative breast carcinomas of low malignant potential: review on diagnostic criteria and differential diagnoses
Source: Virchows Arch. 2021 Aug 30;480(1):109–26. doi: 10.1007/s00428-021-03174-7 (PMC8983547; doi:10.1007/s00428-021-03174-7)
Supplement: Supplementary file 24 — (DOC 112 kb) [file 428_2021_3174_MOESM13_ESM.doc]

**Table 2.** List of published cases of classical adenomyoepithelioma and atypical adenomyoepithelioma with data concerning mitotic count and follow-up

| **Authors** | **Notes on cases** | **Mitoses**  **/10 HPF** | **Follow-up** | | **Rec**  **(N°)** | **Met**  **(N°)** | **Mitoses**  **/10HPF**  **Rec/Met** | **Follow-up Rec** | | **Follow-up Met** | | | |
| --- | --- | --- | --- | --- | --- | --- | --- | --- | --- | --- | --- | --- | --- |
|  |  |  | **Months** | **Years** |  |  |  | **Months** | **Years** | | **Months** | **Years** |  |
| Eusebi^4^ | C-AME | 0 | 36 | 3 | No | No | - | - | - | | - | - |  |
| Young^5^ | C-AME in adenomyoepithelial adenosis | 3 | 9 | 0.75 | No | No | - | - | - | | - | - |  |
| Young^5^ | C-AME | 1 | 15 | 1.25 | No | No | - | - | - | | - | - |  |
| Young^5^ | C-AME | 0 | 84 | 7 | Yes (3) | No | 1-2 (1°)  1-2 (2°)  1-2 (3°) | 36 (1°)  54 (2°)  84 (3°) | 3 (1°)  4,5 (2°)  7 (3°) | | - | - |  |
| Weidner^9^ | spindle-cell/tubular C-AME | 0 | 6 | 0.5 | No | No | - | - | - | | - | - |  |
| Weidner^9^ | spindle-cell/tubular C-AME | 0 | 10 | 0.83 | No | No | - | - | - | | - | - |  |
| Tavassoli^12^ | tubular C-AME | 3 | 102 | 8.5 | Yes  (2) | No | 3 (1°)  3 (2°) | 64 (1°)  77 (2°) | 5.3 (1°)  6.4 (2°) | | - | - |  |
| Tavassoli^12^ | lobulated C-AME | 3 | 8 | 0.66 | Yes  (1*) | No | 8** | 8 | 0.66 | | - | - |  |
| Tavassoli^12^ | lobulated C-AME | 10 | 73.2*** | 6.1*** | No | No | - | - | - | | - | - |  |
| Tavassoli^12^ | lobulated C-AME | 10 | 73.2*** | 6.1*** | No | No | - | - | - | | - | - |  |
| Tavassoli^12^ | lobulated C-AME | 10 | 73.2*** | 6.1*** | No | No | - | - | - | | - | - |  |
| Tavassoli^12^ | lobulated C-AME | 1-2 | 73.2*** | 6.1*** | No | No | - | - | - | | - | - |  |
| Tavassoli^12^ | lobulated C-AME | 1-2 | 73.2*** | 6.1*** | No | No | - | - | - | | - | - |  |
| Tavassoli^12^ | lobulated C-AME | 1-2 | 73.2*** | 6.1*** | No | No | - | - | - | | - | - |  |
| Tavassoli^12^ | lobulated C-AME | 1-2 | 73.2*** | 6.1*** | No | No | - | - | - | | - | - |  |
| Tavassoli^12^ | lobulated C-AME | 1-2 | 73.2*** | 6.1*** | No | No | - | - | - | | - | - |  |
| Tavassoli^12^ | lobulated C-AME | 1-2 | 73.2*** | 6.1*** | No | No | - | - | - | | - | - |  |
| Tavassoli^12^ | lobulated C-AME | 1-2 | 73.2*** | 6.1*** | No | No | - | - | - | | - | - |  |
| Tavassoli^12^ | lobulated C-AME | 1-2 | 73.2*** | 6.1*** | No | No | - | - | - | | - | - |  |
| Loose^14^ | tubular C-AME | 9 | 55 | 4.58 | Yes  (1) | No | 2 | 48 | 4 | | - | - |  |
| Loose^14^ | lobulated C-AME | 3 | 21 | 1.75 | Yes (1) | No | 1 | 60 | 5 | | - | - |  |
| Loose^14^ | tubular C-AME | 2 | 21 | 1.75 | No | No | - | - | - | | - | - |  |
| Loose^14^ | tubular C-AME | 1 | 5 | 0.41 | No | No | - | - | - | | - | - |  |
| Tamura^17^ | spindle-cell C-AME in a male | 1-2 | 3/0.69**** | - | No | No | - | - | - | | - | - |  |
| Pauwels^22^ | tubular C-AME | 3 | 72 | 6 | Yes  (2) | No | 3 (1°)  16 (2°) | 48 (1°)  72 (2°) | 4 (1°)  6 (2°) | | - | - |  |
| Nomura^32^ | A-AME | 10 (EC)  1-2 (MC) | 30 | 2.5 | No | No | - | - | - | | - | - |  |
| Laforga^37^ | cystic C-AME with intranuclear inclusions | 2 | 15 | 1.25 | No | No | - | - | - | | - | - |  |
| Laforga^37^ | cystic C-AME with intranuclear inclusions | 1 | 11 | 0.91 | No | No | - | - | - | | - | - |  |
| Rasbridge^39^ | A-AME | 6 | 210 | 17.5 | Yes  (2) | No | 6 (1°)  16 (2°) | 180 (1°)  210 (2°) | 15 (1°)  17.5 (2°) | | - | - |  |
| Rasbridge^39^ | A-AME | 16 | 6 | 0.5 | Yes  (1) | No | NA | 6 | 0.5 | | - | - |  |
| Rasbridge^39^ | A-AME | 13 | 36 | 3 | No | Yes | - | - | - | | 36 | 3 |  |
| Gill^42^ | C-AME | 0 | 18 | 1.5 | No | No | - | - | - | | - | - |  |
| Gill^42^ | C-AME | 0 | 10 | 0.83 | No | No | - | - | - | | - | - |  |
| Zhang^60^ | A-AME | 4 | 12 | 1 | No | No | - | - | - | | - | - |  |
| Reis-Filho^61^ | C-AME with CS | 2 | 6 | 0.5 | No | No | - | - | - | | - | - |  |
| Gatalica^70^ | C-AME with t (8; 16) (p23; q21) | 1 | 36 | 3 | No | No | - | - | - | | - | - |  |
| Nadelman^73^ | C-AME with lung met | 3 | 72 | 6 | Yes  (2) | Yes  ***** | NA | 48 (1°)  60 (2°) | 4 (1°)  5 (2°) | | 72 | 6 |  |
| Nadelman^73^ | Metastasizing C-AME | 3 | 12 | 1 | No | Yes  ****** | NA | - | - | | 12 | 1 |  |
| Hikino^77^ | intracystic C-AME | 1 | 30 | 2.5 | No | No | - | - | - | | - | - |  |
| Yahara^84^ | C-AME | 0 | 48 | 4 | No | No | - | - | - | | - | - |  |
| Samanta^86^ | Metastasizing C-AME | 1-2 | 18 | 1.5 | No | Yes | NA | - | - | | 18 | 1.5 |  |
| Han^94^ | multicentric A-AME with DCIS | 5 | 43 | 3.58 | No | No | - | - | - | | - | - |  |
| Kamei^111^ | C-AME with DCIS | 1-2 | 40 | 3.33 | No | No | - | - | - | | - | - |  |
| Zhu^115^ | lobulated C-AME | 1 | 10 | 0.83 | No | No | - | - | - | | - | - |  |
| Moritz^120^ | C-AME | 0-2 | 41 | 3.41 | No | No | - | - | - | | - | - |  |
| Moritz^120^ | C-AME | 0-2 | 237 | 19.75 | No | No | - | - | - | | - | - |  |
| Moritz^120^ | C-AME | 0-2 | 137 | 11.41 | No | No | - | - | - | | - | - |  |
| Moritz^120^ | C-AME | 0-2 | 97 | 8.08 | No | No | - | - | - | | - | - |  |
| Moritz^120^ | C-AME | 0-2 | 113 | 9.41 | No | No | - | - | - | | - | - |  |
| Moritz^120^ | C-AME | 0-2 | 83 | 6.92 | No | No | - | - | - | | - | - |  |
| Moritz^120^ | C-AME | 0-2 | 62 | 5.16 | No | No | - | - | - | | - | - |  |
| Moritz^120^ | C-AME | 0-2 | 62 | 5.16 | No | No | - | - | - | | - | - |  |
| Moritz^120^ | C-AME | 0-2 | 35.5 | 2.96 | No | No | - | - | - | | - | - |  |
| Moritz^120^ | C-AME | 0-2 | 15 | 1.25 | No | No | - | - | - | | - | - |  |
| Moritz^120^ | C-AME | 0-2 | <1 | - | No | No | - | - | - | | - | - |  |
| Harada^146^ | C-AME with PA-like component in a male | 0 | 5 | 0.41 | No | No | - | - | - | | - | - |  |
| Amano^147^ | C-AME with DCIS and HER2 expression/amplification | 0 | 23 | 1.91 | No | No | - | - | - | | - | - |  |
| El-Helou^148^ | C-AME | 1 | 5 | 0.41 | No | No | - | - | - | | - | - |  |

Abbreviations, HPF: high power field hight Rec: recurrence; N°: number; Met: metastasis; C-AME: classical adenomyoepithelioma; A-AME: atypical adenomyoepithelioma; EC: epithelial component; MC: myoepithelial component; NA: not available; CS: collagenous spherulosis; DCIS: ductal carcinoma in situ; PA: pleomorphic adenoma.

*four simultaneous recurrent nodules ** 8 mitoses/10 HPF in all the nodules *** average time ****weeks/months *****five-seven simultaneous metastatic nodules ******initial presentation
