# Supplementary material for: Triple-negative breast carcinomas of low malignant potential: review on diagnostic criteria and differential diagnoses
Source: Virchows Arch. 2021 Aug 30;480(1):109–26. doi: 10.1007/s00428-021-03174-7 (PMC8983547; doi:10.1007/s00428-021-03174-7)
Supplement: Supplementary file 25 — (DOC 73 kb) [file 428_2021_3174_MOESM14_ESM.doc]

1. Cameron HM, Hamperl H, Warambo W. Leiomyosarcoma of the breast originating from myothelium (myoepithelium). J Pathol. 1974 Oct;114(2):89-92. doi: 10.1002/path.1711140206. PMID: 4374517.
2. Zarbo RJ, Oberman HA. Cellular adenomyoepithelioma of the breast. Am J Surg Pathol. 1983 Dec;7(8):863-70. PMID: 6318584.
3. Kiaer H, Nielsen B, Paulsen S, Sørensen IM, Dyreborg U, Blichert-Toft M. Adenomyoepithelial adenosis and low-grade malignant adenomyoepithelioma of the breast. Virchows Arch A Pathol Anat Histopathol. 1984;405(1):55-67. doi: 10.1007/BF00694925. PMID: 6438900.
4. Eusebi V, Casadei GP, Bussolati G, Azzopardi JG. Adenomyoepithelioma of the breast with a distinctive type of apocrine adenosis. Histopathology. 1987 Mar;11(3):305-15. doi: 10.1111/j.1365-2559.1987.tb02635.x. PMID: 2828217.
5. Young RH, Clement PB. Adenomyoepithelioma of the breast. A report of three cases and review of the literature. Am J Clin Pathol. 1988 Mar;89(3):308-14. doi: 10.1093/ajcp/89.3.308. PMID: 2831705.
6. Rosen PP. Adenomyoepithelioma of the breast. Hum Pathol. 1987 Dec;18(12):1232-7. doi: 10.1016/s0046-8177(87)80406-9. PMID: 2824328.
7. Jabi M, Dardick I, Cardigos N. Adenomyoepithelioma of the breast. Arch Pathol Lab Med. 1988 Jan;112(1):73-6. PMID: 2447854.
8. Tamura S, Enjoji M, Toyoshima S, Terasaka R. Adenomyoepithelioma of the breast. A case report with an immunohistochemical study. Acta Pathol Jpn. 1988 May;38(5):659-65. PMID: 2850707.
9. Weidner N, Levine JD. Spindle-cell adenomyoepithelioma of the breast. A microscopic, ultrastructural, and immunocytochemical study. Cancer. 1988 Oct 15;62(8):1561-7. doi: 10.1002/1097-0142(19881015)62:8<1561::aid-cncr2820620817>3.0.co;2-z. PMID: 2844382.
10. Erlandson RA. Benign adenomyoepithelioma of the breast. Ultrastruct Pathol. 1989 Mar-Jun;13(2-3):307-14. doi: 10.3109/01913128909057449. PMID: 2544054.
11. Accurso A, Donofrio V, Insabato L, Mosella G. Adenomyoepithelioma of the breast. A case report. Tumori. 1990 Dec 31;76(6):606-10. PMID: 2178287.
12. Tavassoli FA. Myoepithelial lesions of the breast. Myoepitheliosis, adenomyoepithelioma, and myoepithelial carcinoma. Am J Surg Pathol. 1991 Jun;15(6):554-68. doi: 10.1097/00000478-199106000-00004. PMID: 1709559.
13. Saez A, Serrano T, Aspeitia D, Condom E, Moreno A. Adenomyoepithelioma of the breast. A report of two cases. Arch Pathol Lab Med. 1992 Jan;116(1):36-8. PMID: 1310379.
14. Loose JH, Patchefsky AS, Hollander IJ, Lavin LS, Cooper HS, Katz SM. Adenomyoepithelioma of the breast. A spectrum of biologic behavior. Am J Surg Pathol. 1992 Sep;16(9):868-76. doi: 10.1097/00000478-199209000-00005. PMID: 1384377.
15. Trojani M, Guiu M, Trouette H, De Mascarel I, Cocquet M. Malignant adenomyoepithelioma of the breast. An immunohistochemical, cytophotometric, and ultrastructural study of a case with lung metastases. Am J Clin Pathol. 1992 Dec;98(6):598-602. doi: 10.1093/ajcp/98.6.598. PMID: 1334365.
16. Plaza JA, Lopez JI, Garcia S, de Miguel C. Adenomyoepithelioma of the breast. Report of two cases. Arch Anat Cytol Pathol. 1993;41(2):99-101. PMID: 8239754.
17. Tamura G, Monma N, Suzuki Y, Satodate R, Abe H. Adenomyoepithelioma (myoepithelioma) of the breast in a male. Hum Pathol. 1993 Jun;24(6):678-81. doi: 10.1016/0046-8177(93)90250-k. PMID: 8389319.
18. Vielh P, Thiery JP, Validire P, Annick de Maublanc M, Woto G. Adenomyoepithelioma of the breast: fine-needle sampling with histologic, immunohistologic, and electron microscopic analysis. Diagn Cytopathol. 1993;9(2):188-93. doi: 10.1002/dc.2840090216. PMID: 8390345.
19. Birdsong GG, Bishara HM, Costa MJ. Adenomyoepithelioma of the breast: report of a case initially examined by fine-needle aspiration. Diagn Cytopathol. 1993 Oct;9(5):547-50. doi: 10.1002/dc.2840090515. PMID: 8287764.
20. Michal M, Baumruk L, Burger J, Manhalová M. Adenomyoepithelioma of the breast with undifferentiated carcinoma component. Histopathology. 1994 Mar;24(3):274-6. doi: 10.1111/j.1365-2559.1994.tb00522.x. PMID: 7515373.
21. Chen PC, Chen CK, Nicastri AD, Wait RB. Myoepithelial carcinoma of the breast with distant metastasis and accompanied by adenomyoepitheliomas. Histopathology. 1994 Jun;24(6):543-8. doi: 10.1111/j.1365-2559.1994.tb00573.x. PMID: 7520413.
22. Pauwels C, De Potter C. Adenomyoepithelioma of the breast with features of malignancy. Histopathology. 1994 Jan;24(1):94-6. doi: 10.1111/j.1365-2559.1994.tb01280.x. PMID: 8144150.
23. Diomandé MI, Ehouman A, Boni SA, Bialy C. Réflexions à propos d'un cas d'adénomyoépithéliome du sein [Thoughts apropos of a case of adenomyoepithelioma of the breast]. Arch Anat Cytol Pathol. 1994;42(6):328-9. French. PMID: 7748007.
24. Valente PT, Stuckey JH. Fine-needle aspiration cytology of mammary adenomyoepithelioma: report of a case with intranuclear cytoplasmic inclusions. Diagn Cytopathol. 1994;10(2):165-8. doi: 10.1002/dc.2840100215. PMID: 8187599.
25. Nilsson B, Wee A, Rauff A, Raju GC. Adenomyoepithelioma of the breast. Report of a case with fine needle aspiration cytology and histologic, immunohistochemical and ultrastructural correlation. Acta Cytol. 1994 May-Jun;38(3):431-4. PMID: 8191837.
26. Hock YL, Chan SY. Adenomyoepithelioma of the breast. A case report correlating cytologic and histologic features. Acta Cytol. 1994 Nov-Dec;38(6):953-6. PMID: 7992585.
27. Niemann TH, Benda JA, Cohen MB. Adenomyoepithelioma of the breast: fine-needle aspiration biopsy and histologic findings. Diagn Cytopathol. 1995 May;12(3):245-50. doi: 10.1002/dc.2840120311. PMID: 7621720.
28. Foschini MP, Pizzicannella G, Peterse JL, Eusebi V. Adenomyoepithelioma of the breast associated with low-grade adenosquamous and sarcomatoid carcinomas. Virchows Arch. 1995;427(3):243-50. doi: 10.1007/BF00203390. PMID: 7496592.
29. Khattech R, Ben Othman M, Ben Romdhane K, Gamoudi A, Ammar A. Le myoépithéliome du sein. A propos d'un cas [Breast myoepithelioma. Report of a case]. Ann Pathol. 1995;15(2):138-41. French. PMID: 7755804.
30. Meunier B, Levêque J, Le Prise E, Tas P, Grall JY. Adéno-myoépithéliome du sein. A propos d'un cas et revue de la littérature [Breast adenomyoepithelioma. A case report and review of the literature]. J Gynecol Obstet Biol Reprod (Paris). 1995;24(2):158-61. French. PMID: 7782587.
31. Choi JS, Bae JY, Jung WH. Adenomyoepithelioma of the breast--its diagnostic problems and histogenesis. Yonsei Med J. 1996 Aug;37(4):284-9. doi: 10.3349/ymj.1996.37.4.284. PMID: 8942299.
32. Nomura K, Fukunaga M, Uchida K, Aizawa S. Adenomyoepithelioma of the breast with exaggerated proliferation of epithelial cells: report of a case. Pathol Int. 1996 Dec;46(12):1011-4. doi: 10.1111/j.1440-1827.1996.tb03582.x. PMID: 9110355.
33. Park Y, Okuyama N, Hatano Y, Kato N, Sasamoto S, Shimatani S, Yamazaki S, Yanagida M. Adenomyoepithelioma of the Breast: A Case Reprot and a Review of Literature. Breast Cancer. 1996 Mar 29;3(1):65-69. doi: 10.1007/BF02966965. PMID: 11091556.
34. Pogacnik A, Golouh R, Flezar M. Adenomyoepithelioma of the breast diagnosed by fine needle aspiration (FNA) biopsy; a case report. Cytopathology. 1997 Feb;8(1):45-52. doi: 10.1046/j.1365-2303.1997.43275432.x. PMID: 9068956.
35. McCluggage WG, McManus DI, Caughley LM. Fine needle aspiration (FNA) cytology of adenoid cystic carcinoma and adenomyoepithelioma of breast: two lesions rich in myoepithelial cells. Cytopathology. 1997 Feb;8(1):31-9. doi: 10.1046/j.1365-2303.1997.43975439.x. PMID: 9068954.
36. Van Dorpe J, De Pauw A, Moerman P. Adenoid cystic carcinoma arising in an adenomyoepithelioma of the breast. Virchows Arch. 1998 Feb;432(2):119-22. doi: 10.1007/s004280050144. PMID: 9504855.
37. Laforga JB, Aranda FI, Sevilla F. Adenomyoepithelioma of the breast: report of two cases with prominent cystic changes and intranuclear inclusions. Diagn Cytopathol. 1998 Jul 1;19(1):55-8. doi: 10.1002/(sici)1097-0339(199807)19:1<55::aid-dc11>3.0.co;2-7. PMID: 9664185.
38. Gupta RK, Dowle CS. Immunocytochemical study in a case of adenomyoepithelioma of the breast. Diagn Cytopathol. 1998 Jun;18(6):468-70. doi: 10.1002/(sici)1097-0339(199806)18:6<468::aid-dc17>3.0.co;2-f. PMID: 9626524.
39. Rasbridge SA, Millis RR. Adenomyoepithelioma of the breast with malignant features. Virchows Arch. 1998 Feb;432(2):123-30. doi: 10.1007/s004280050145. PMID: 9504856.
40. Simpson RH, Cope N, Skálová A, Michal M. Malignant adenomyoepithelioma of the breast with mixed osteogenic, spindle cell, and carcinomatous differentiation. Am J Surg Pathol. 1998 May;22(5):631-6. doi: 10.1097/00000478-199805000-00015. PMID: 9591734.
41. Takahashi I I, Tashiro H, Wakasugi K, Onohara T, Nishizaki T, Ishikawa T, Matsusaka T, Kume K, Yamamoto I I. Malignant Adenomyoepithelioma of the Breast:A Case with Distant Metastases. Breast Cancer. 1999 Jan 25;6(1):73-77. doi: 10.1007/BF02966911. PMID: 11091695.
42. Singh Gill T, Clarke D, Douglas-Jones AG, Sweetland HM, Mansel RE. Adenomyoepithelioma of the breast: a diagnostic dilemma. Eur J Surg Oncol. 2000 Apr;26(3):316-8. doi: 10.1053/ejso.1999.0890. PMID: 10753541.
43. Ahmed AA, Heller DS. Malignant adenomyoepithelioma of the breast with malignant proliferation of epithelial and myoepithelial elements: a case report and review of the literature. Arch Pathol Lab Med. 2000 Apr;124(4):632-6. doi: 10.1043/0003-9985(2000)124<0632:MAOTBW>2.0.CO;2. PMID: 10747327.
44. Bult P, Verwiel JM, Wobbes T, Kooy-Smits MM, Biert J, Holland R. Malignant adenomyoepithelioma of the breast with metastasis in the thyroid gland 12 years after excision of the primary tumor. Case report and review of the literature. Virchows Arch. 2000 Feb;436(2):158-66. doi: 10.1007/pl00008216. PMID: 10755607.
45. Lee WY. Fine needle aspiration cytology of adenomyoepithelioma of the breast: a case indistinguishable from phyllodes tumor in cytologic findings and clinical behavior. Acta Cytol. 2000 May-Jun;44(3):488-90. PMID: 10834021.
46. Rammeh-Rommani S, Mrad K, Dhouib R, Sassi S, Driss M, Khattech R, Ben Romdhane K. Les adénomyoépithéliomes du sein: à propos de deux cas [Adenomyoepithelioma of the breast: two cases]. Ann Pathol. 2000 Sep;20(4):365-8. French. PMID: 11015657.
47. Tukel S, Ustuner E, Aytac SK. Adenomyoepithelioma of the breast. J Ultrasound Med. 2001 Sep;20(9):1021-4. doi: 10.7863/jum.2001.20.9.1021. PMID: 11549151.
48. Aydin O, Cinel L, Egilmez R, Ocal K, Ozer C. Adenomyoepithelioma of the breast. Diagn Cytopathol. 2001 Sep;25(3):194-6. doi: 10.1002/dc.2036. PMID: 11536445.
49. Kihara M, Yokomise H, Irie A, Kobayashi S, Kushida Y, Yamauchi A. Malignant adenomyoepithelioma of the breast with lung metastases: report of a case. Surg Today. 2001;31(10):899-903. doi: 10.1007/s005950170031. PMID: 11759886.
50. Sugano I, Nagao T, Tajima Y, Ishida Y, Nagao K, Ooeda Y, Takahashi T. Malignant adenomyoepithelioma of the breast: a non-tubular and matrix-producing variant. Pathol Int. 2001 Mar;51(3):193-9. doi: 10.1046/j.1440-1827.2001.01178.x. PMID: 11328535.
51. Viguer JM, López-Ferrer P, Vicandi B, Jiménez-Heffernan JA. Cytologic features of the tubular variant of breast adenomyoepithelioma. Acta Cytol. 2001 Nov-Dec;45(6):1090-2. PMID: 11726113.
52. Chang A, Bassett L, Bose S. Adenomyoepithelioma of the breast: a cytologic dilemma. Report of a case and review of the literature. Diagn Cytopathol. 2002 Mar;26(3):191-6. doi: 10.1002/dc.10073. PMID: 11892027.
53. Felipo F, Del Agua C, Eguizabal C, Vaquero M. Benign adenomyoepithelioma of the breast: a case with gross mimicking of malignancy. Breast J. 2002 Nov-Dec;8(6):383-4. doi: 10.1046/j.1524-4741.2002.08610.x. PMID: 12390363.
54. Ng WK. Adenomyoepithelioma of the breast. A review of three cases with reappraisal of the fine needle aspiration biopsy findings. Acta Cytol. 2002 Mar-Apr;46(2):317-24. doi: 10.1159/000326728. PMID: 11917579.
55. Kinkor Z. Myoepiteliálníkarcinom v adenomyoepiteliomu prsu (maligní adenomyoepiteliom)--kazuistika [Myoepithelial carcinoma in adenomyoepithelioma of the breast (malignant adenomyoepithelioma)--case report]. Cesk Patol. 2002 Jan;38(1):46-50. Czech. PMID: 11933463.
56. Kurashina M. Fine-needle aspiration cytology of benign and malignant adenomyoepithelioma: report of two cases. Diagn Cytopathol. 2002 Jan;26(1):29-34. doi: 10.1002/dc.10037. PMID: 11782084.
57. Popnikolov NK, Ayala AG, Graves K, Gatalica Z. Benign myoepithelial tumors of the breast have immunophenotypic characteristics similar to metaplastic matrix-producing and spindle cell carcinomas. Am J Clin Pathol. 2003 Aug;120(2):161-7. doi: 10.1309/G6CT-R8MD-TFUW-19XV. PMID: 12931544.
58. Howlett DC, Mason CH, Biswas S, Sangle PD, Rubin G, Allan SM. Adenomyoepithelioma of the breast: spectrum of disease with associated imaging and pathology. AJR Am J Roentgenol. 2003 Mar;180(3):799-803. doi: 10.2214/ajr.180.3.1800799. PMID: 12591699.
59. Jones C, Tooze R, Lakhani SR. Malignant adenomyoepithelioma of the breast metastasizing to the liver. Virchows Arch. 2003 May;442(5):504-6. doi: 10.1007/s00428-003-0806-2. Epub 2003 Apr 12. PMID: 12692727.
60. Zhang C, Quddus MR, Sung CJ. Atypical adenomyoepithelioma of the breast: diagnostic problems and practical approaches in core needle biopsy. Breast J. 2004 Mar-Apr;10(2):154-5. doi: 10.1111/j.1075-122x.2004.21414.x. PMID: 15009045.
61. Reis-Filho JS, Fulford LG, Crebassa B, Carpentier S, Lakhani SR. Collagenous spherulosis in an adenomyoepithelioma of the breast. J Clin Pathol. 2004 Jan;57(1):83-6. doi: 10.1136/jcp.57.1.83. PMID: 14693844; PMCID: PMC1770151.
62. Harigopal M, Park K, Chen X, Rosen PP. Pathologic quiz case: a rapidly increasing breast mass in a postmenopausal woman. Malignant adenomyoepithelioma. Arch Pathol Lab Med. 2004 Feb;128(2):235-6. doi: 10.1043/1543-2165(2004)128<235:PQCARI>2.0.CO;2. PMID: 14736274.
63. Papaevangelou A, Pougouras I, Liapi G, Pierrakakis S, Tibishrani M, Setakis N. Cystic adenomyoepithelioma of the breast. Breast. 2004 Aug;13(4):356-8. doi: 10.1016/j.breast.2003.11.006. PMID: 15325675.
64. Mathur SR, Karak K, Verma K. Adenomyoepithelioma of the breast: a potential diagnostic pitfall on fine needle aspiration cytology. Indian J Pathol Microbiol. 2004 Apr;47(2):243-5. PMID: 16295485.
65. Gatti G, Viale G, Simsek S, Zurrida S, Intra M, Caldarella P, Luini A. Adenomyoepithelioma of the breast, presenting as a cancer. Tumori. 2004 May-Jun;90(3):337-9. PMID: 15315316.
66. Loh HL, Kumarasinghe P, Tan PH. Test and Teach. Recurrent breast lumps in a Chinese woman. Adenomyoepithelioma of the breast. Pathology. 2004 Jun;36(3):269-72. doi: 10.1080/00313020410001692611. PMID: 15203734.
67. McLaren BK, Smith J, Schuyler PA, Dupont WD, Page DL. Adenomyoepithelioma: clinical, histologic, and immunohistologic evaluation of a series of related lesions. Am J Surg Pathol. 2005 Oct;29(10):1294-9. doi: 10.1097/01.pas.0000164615.38200.86. PMID: 16160470.
68. Cai RZ, Tan PH. Adenomyoepithelioma of the breast with squamous and sebaceous metaplasia. Pathology. 2005 Dec;37(6):557-9. doi: 10.1080/00313020500368394. PMID: 16373236.
69. Salto-Tellez M, Putti TC, Lee CK, Chiu LL, Koay ES. Adenomyoepithelioma of the breast: description of allelic imbalance and microsatellite instability. Histopathology. 2005 Feb;46(2):230-1. doi: 10.1111/j.1365-2559.2004.01984.x. PMID: 15693897.
70. Gatalica Z, Velagaleti G, Kuivaniemi H, Tromp G, Palazzo J, Graves KM, Guigneaux M, Wood T, Sinha M, Luxon B. Gene expression profile of an adenomyoepithelioma of the breast with a reciprocal translocation involving chromosomes 8 and 16. Cancer Genet Cytogenet. 2005 Jan 1;156(1):14-22. doi: 10.1016/j.cancergencyto.2004.04.024. PMID: 15588851.
71. Okada Y, Takeuchi T, Ohtsuki Y, Hachisuka Y, Kimura M, Watanabe R, Numoto S. Loose intercellular adhesion in adenomyoepithelioma of the breast. Cytopathology. 2005 Oct;16(5):265-6. doi: 10.1111/j.1365-2303.2005.00242.x. PMID: 16181318.
72. Tait R, Pinder SE, Ellis IO, Purushotham AD. Adenomyoepithelioma of the breast; a case report and literature review. J BUON. 2005 Jul-Sep;10(3):393-5. PMID: 17357195.
73. Nadelman CM, Leslie KO, Fishbein MC. "Benign," metastasizing adenomyoepithelioma of the breast: a report of 2 cases. Arch Pathol Lab Med. 2006 Sep;130(9):1349-53. doi: 10.1043/1543-2165(2006)130[1349:BMAOTB]2.0.CO;2. PMID: 16948523.
74. Han B, Mori I, Nakamura M, Wang X, Ozaki T, Nakamura Y, Kakudo K. Myoepithelial carcinoma arising in an adenomyoepithelioma of the breast: case report with immunohistochemical and mutational analysis. Pathol Int. 2006 Apr;56(4):211-6. doi: 10.1111/j.1440-1827.2006.01948.x. PMID: 16634967.
75. Buch A, Rout P, Makhija P. Adenomyoepithelioma with phyllodes tumor--a rare combination in a solitary breast lump. Indian J Pathol Microbiol. 2006 Apr;49(2):259-61. Erratum in: Indian J Pathol Microbiol. 2006 Jul;49(3):476. PMID: 16933731.
76. Noël JC, Simon P, Aguilar SF. Malignant myoepithelioma arising in cystic adenomyoepithelioma. Breast J. 2006 Jul-Aug;12(4):386. doi: 10.1111/j.1075-122X.2006.00286.x. PMID: 16848857
77. Hikino H, Kodama K, Yasui K, Ozaki N, Nagaoka S, Miura H. Intracystic adenomyoepithelioma of the breast--case report and review. Breast Cancer. 2007;14(4):429-33. doi: 10.2325/jbcs.14.429. PMID: 17986811.
78. Oka K, Sando N, Moriya T, Yatabe Y. Malignant adenomyoepithelioma of the breast with matrix production may be compatible with one variant form of matrix-producing carcinoma: a case report. Pathol Res Pract. 2007;203(8):599-604. doi: 10.1016/j.prp.2007.04.004. Epub 2007 Jul 19. PMID: 17658224.
79. Fan F, Smith W, Wang X, Jewell W, Thomas PA, Tawfik O. Myoepithelial carcinoma of the breast arising in an adenomyoepithelioma: mammographic, ultrasound and histologic features. Breast J. 2007 Mar-Apr;13(2):203-4. doi: 10.1111/j.1524-4741.2007.00403.x. PMID: 17319866.
80. Mercado CL, Toth HK, Axelrod D, Cangiarella J. Fine-needle aspiration biopsy of benign adenomyoepithelioma of the breast: radiologic and pathologic correlation in four cases. Diagn Cytopathol. 2007 Nov;35(11):690-4. doi: 10.1002/dc.20722. PMID: 17924402.
81. Ruiz-Delgado ML, López-Ruiz JA, Eizaguirre B, Saiz A, Astigarraga E, Fernández-Temprano Z. Benign adenomyoepithelioma of the breast: imaging findings mimicking malignancy and histopathological features. Acta Radiol. 2007 Feb;48(1):27-9. doi: 10.1080/02841850601080432. PMID: 17325921.
82. Huang CY, Sheen-Chen SM, Eng HL, Ko SF. Adenomyoepithelioma of the breast. Tumori. 2007 Sep-Oct;93(5):493-5. PMID: 18038884.
83. Catena F, Santini D, Di Saverio S, Ansaloni L, Taffurelli M. Adenomyoepithelioma of the Breast: An Intricate Diagnostic Problem. Breast Care (Basel). 2008;3(2):125-127. doi: 10.1159/000119727. Epub 2008 Apr 15. PMID: 21373216; PMCID: PMC2931087.
84. Yahara T, Yamaguchi R, Yokoyama G, Yamaguchi M, Nakagawa S, Toh U, Shirouzu K, Kage M, Fujii T. Adenomyoepithelioma of the breast diagnosed by a mammotome biopsy: report of a case. Surg Today. 2008;38(2):144-6. doi: 10.1007/s00595-007-3591-8. Epub 2008 Feb 1. PMID: 18239872.
85. Kuroda N, Fujishima N, Ohara M, Hirouchi T, Mizuno K, Hayashi Y, Lee GH. Coexistent adenomyoepithelioma and invasive ductal carcinoma of the breast: presentation as separate tumors. Med Mol Morphol. 2008 Dec;41(4):238-42. doi: 10.1007/s00795-008-0376-0. Epub 2008 Dec 24. PMID: 19107615.
86. Samanta DR, Senapati SN, Sharma PK, Mohanty AK. Adenomyoepithelioma of the breast. Hematol Oncol Stem Cell Ther. 2009;2(2):364-6. doi: 10.1016/s1658-3876(09)50028-3. PMID: 20118063.
87. Qureshi A, Kayani N, Gulzar R. Malignant adenomyoepithelioma of the breast: a case report with review of literature. BMJ Case Rep. 2009;2009:bcr01.2009.1442. doi: 10.1136/bcr.01.2009.1442. Epub 2009 Jun 18. PMID: 21687020; PMCID: PMC3027707.
88. Honda Y, Iyama K. Malignant adenomyoepithelioma of the breast combined with invasive lobular carcinoma. Pathol Int. 2009 Mar;59(3):179-84. doi: 10.1111/j.1440-1827.2009.02347.x. PMID: 19261096.
89. Hegyi L, Thway K, Newton R, Osin P, Nerurkar A, Hayes AJ, Fisher C. Malignant myoepithelioma arising in adenomyoepithelioma of the breast and coincident multiple gastrointestinal stromal tumours in a patient with neurofibromatosis type 1. J Clin Pathol. 2009 Jul;62(7):653-5. doi: 10.1136/jcp.2008.063628. PMID: 19561236.
90. Zizi-Sermpetzoglou A, Vasilakaki T, Grammatoglou X, Petrakopoulou N, Nikolaidou ME, Glava C. Malignant adenomyoepithelioma of the breast--case report. Eur J Gynaecol Oncol. 2009;30(2):234-6. PMID: 19480267.
91. Hayes MM. Adenomyoepithelioma of the breast: a review stressing its propensity for malignant transformation. J Clin Pathol. 2011 Jun;64(6):477-84. doi: 10.1136/jcp.2010.087718. Epub 2011 Feb 9. PMID: 21307156.
92. Buza N, Zekry N, Charpin C, Tavassoli FA. Myoepithelial carcinoma of the breast: a clinicopathological and immunohistochemical study of 15 diagnostically challenging cases. Virchows Arch. 2010 Sep;457(3):337-45. doi: 10.1007/s00428-010-0950-4. Epub 2010 Jul 24. PMID: 20658149.
93. Ohta M, Mori M, Kawada T, Maegawa H, Yamamoto S, Imamura Y. Collagenous spherulosis associated with adenomyoepithelioma of the breast: a case report. Acta Cytol. 2010 May-Jun;54(3):314-8. doi: 10.1159/000325041. PMID: 20518417.
94. Han JS, Peng Y. Multicentric adenomyoepithelioma of the breast with atypia and associated ductal carcinoma in situ. Breast J. 2010 Sep-Oct;16(5):547-9. doi: 10.1111/j.1524-4741.2010.00954.x. Epub 2010 Jun 29. PMID: 20604795.
95. Khurana A, Jalpota Y. Myoepithelial carcinoma arising in an adenomyoepithelioma of the breast: a case report of a rare entity. Indian J Pathol Microbiol. 2010 Apr-Jun;53(2):310-2. doi: 10.4103/0377-4929.64296. PMID: 20551541.
96. Gandhi JS, Mehta A, Gupta G, Sardana M. Malignant adenomyoepithelioma: a tumor of low malignant potential despite basal phenotype. Indian J Pathol Microbiol. 2011 Jan-Mar;54(1):230-2. doi: 10.4103/0377-4929.77421. PMID: 21393936.
97. Adejolu M, Wu Y, Santiago L, Yang WT. Adenomyoepithelial tumors of the breast: imaging findings with histopathologic correlation. AJR Am J Roentgenol. 2011 Jul;197(1):W184-90. doi: 10.2214/AJR.10.6064. PMID: 21700984.
98. Matsumoto K, Enomoto K, Sakura K. [A case of breast adenomyoepithelioma that was diagnosed by local excision]. Gan To Kagaku Ryoho. 2011 Nov;38(12):2168-70. Japanese. PMID: 22202318.
99. Saad RS, Richmond L, Nofech-Mozes S, Ghorab Z. Fine-needle aspiration biopsy of breast adenomyoepithelioma: a potential false positive pitfall and presence of intranuclear cytoplasmic inclusions. Diagn Cytopathol. 2012 Nov;40(11):1005-9. doi: 10.1002/dc.21705. Epub 2011 May 4. PMID: 21548117.
100. Awamleh AA, Gudi M, Shousha S. Malignant adenomyoepithelioma of the breast with lymph node metastasis: a detailed immunohistochemical study. Case Rep Pathol. 2012;2012:305858. doi: 10.1155/2012/305858. Epub 2012 Jan 18. PMID: 22934213; PMCID: PMC3420733.
101. Maffini F, Renne G, Olivadese R, Solli P, Locatelli M, Pruneri G, Barberis M, Viale G. A rare case of lung metastasis from a malignant adenomyoepithelioma of the breast: histological features and therapeutic implications. Ecancermedicalscience. 2013 Nov 12;7:372. doi: 10.3332/ecancer.2013.372. PMID: 24244218; PMCID: PMC3826808.
102. Bajpai J, Punatar SB, Gupta A, Badwe R, Gupta S. Bilateral adenomyoepithelioma of breast. J Cancer Res Ther. 2013 Jul-Sep;9(3):523-5. doi: 10.4103/0973-1482.119370. PMID: 24125999.
103. Petrozza V, Pasciuti G, Pacchiarotti A, Tomao F, Zoratto F, Rossi L, Fontana A, Censi F, Sardella B, Di Cristofano C, Porta N, Della Rocca C. Breast adenomyoepithelioma: a case report with malignant proliferation of epithelial and myoepithelial elements. World J Surg Oncol. 2013 Oct 30;11:285. doi: 10.1186/1477-7819-11-285. PMID: 24171817; PMCID: PMC3816540.
104. Marian C, Boila A, Soanca D, Malau M, Podeanu DM, Resetkova E, Stolnicu S. Malignant transformation of adenomyoepithelioma of the breast by a monophasic population: a report of two cases and review of literature. APMIS. 2013 Apr;121(4):272-9. doi: 10.1111/j.1600-0463.2012.02982.x. Epub 2012 Sep 13. PMID: 23030630.
105. Warrier S, Hwang S, Ghaly M, Matthews A. Adenomyoepithelioma with ductal carcinoma in situ: a case report and review of the literature. Case Rep Surg. 2013;2013:521417. doi: 10.1155/2013/521417. Epub 2013 Feb 25. PMID: 23533914; PMCID: PMC3596908.
106. Wang LB, Chen HY, Ma WB, Lu JP. [Malignant adenomyoepithelioma of breast with lymph node metastasis: report of a case]. Zhonghua Bing Li Xue Za Zhi. 2013 Jun;42(6):408-9. Chinese. doi: 10.3760/cma.j.issn.0529-5807.2013.06.014. PMID: 24060079.
107. Yang Y, Wang Y, He J, Pan G, Tuo X, Jiang A, Bian L. Malignant adenomyoepithelioma combined with adenoid cystic carcinoma of the breast: a case report and literature review. Diagn Pathol. 2014 Jul 23;9:148. doi: 10.1186/1746-1596-9-148. PMID: 25056281; PMCID: PMC4222729.
108. R K, Murthy V S. Malignant adenomyoepithelioma of breast masquerading as soft tissue lytic lesion of right iliac bone: a rare entity. J Clin Diagn Res. 2014 Sep;8(9):FD03-4. doi: 10.7860/JCDR/2014/8959.4780. Epub 2014 Sep 20. PMID: 25386443; PMCID: PMC4225895.
109. Robinson A, Mayne J, Boroumand N, Silva HC, Eltorky M, Mayne J. Malignant adenomyoepithelioma: a rare entity. Breast J. 2014 Jan-Feb;20(1):94-6. doi: 10.1111/tbj.12214. Epub 2013 Nov 22. PMID: 24261872.
110. Wakasa T, Nakamura M, Kagiya T, Taniguchi E, Sakurai T, Kakudo K. Loss of cellular cohesion in cytology composes a special subgroup of breast tumors - analysis of 37 cases. Acta Cytol. 2014;58(1):89-95. doi: 10.1159/000356405. Epub 2013 Dec 10. PMID: 24334922.
111. Kamei M, Daa T, Miyawaki M, Suehiro S, Sugio K. Adenomyoepithelioma of the breast coexisting with ductal carcinoma in situ: a case report and review of the literature. Surg Case Rep. 2015 Sep 11;1:81. doi: 10.1186/s40792-015-0083-8. PMID: 26380805; PMCID: PMC4567590.
112. Zhang S, Huo L, Arribas E, Middleton LP. Adenomyoepithelioma of the breast with associated atypical lobular hyperplasia: a previously unrecognized association with management implications. Ann Diagn Pathol. 2015 Feb;19(1):20-3. doi: 10.1016/j.anndiagpath.2014.11.002. Epub 2014 Dec 13. PMID: 25631879.
113. Delteil C, Jalaguier Coudray A, Charafe Jauffret E, Thomassin Piana J. Adénomyoépithéliome avec contingent myoépithélial prédominant du sein : à propos d'une observation et revue de la littérature [Adenomyoepithelioma with dominant myoepithelial contingent of the breast: A case report and literature review]. Ann Pathol. 2015 Oct;35(5):449-53. French. doi: 10.1016/j.annpat.2015.05.006. Epub 2015 Sep 16. PMID: 26386768.
114. Gkali CA, Chalazonitis AN, Feida E, Dimitrakakis C, Sotiropoulou M. Breast Adenomyoepithelioma: Ultrasonography, Elastography, Digital Mammography, Contrast-Enhanced Digital Mammography, and Pathology Findings of This Rare Type of Breast Tumor. Ultrasound Q. 2015 Sep;31(3):185-8. doi: 10.1097/RUQ.0000000000000119. PMID: 26366690.
115. Zhu J, Ni G, Wang D, He Q, Li P. Lobulated adenomyoepithelioma: a case report showing immunohistochemical profiles. Int J Clin Exp Pathol. 2015 Nov 1;8(11):15407-11. PMID: 26823903; PMCID: PMC4713689.
116. Ahmadi N, Negahban S, Aledavood A, Daneshbod K, Daneshbod Y. Malignant adenomyoepithelioma of the breast: a review. Breast J. 2015 May-Jun;21(3):291-6. doi: 10.1111/tbj.12390. Epub 2015 Mar 13. PMID: 25772218.
117. Lee S, Oh SY, Kim SH, Lee JH, Kim DC, Cho SH, Lee M, Kim HJ. Malignant Adenomyoepithelioma of the Breast and Responsiveness to Eribulin. J Breast Cancer. 2015 Dec;18(4):400-3. doi: 10.4048/jbc.2015.18.4.400. Epub 2015 Dec 23. PMID: 26770248; PMCID: PMC4705093.
118. Moritani S, Ichihara S, Yatabe Y, Hasegawa M, Iwakoshi A, Hosoda W, Narita M, Nagai Y, Asai M, Ujihira N, Yuba Y, Jijiwa M. Immunohistochemical expression of myoepithelial markers in adenomyoepithelioma of the breast: a unique paradoxical staining pattern of high-molecular weight cytokeratins. Virchows Arch. 2015 Feb;466(2):191-8. doi: 10.1007/s00428-014-1687-2. Epub 2014 Dec 6. PMID: 25479938.
119. Korolczuk A, Amarowicz M, Bąk K, Korobowicz E, Koncewicz T. Adenomyoepithelioma of the breast with late pulmonary metastases - case report and review of the literature. J Cardiothorac Surg. 2016 Aug 4;11(1):121. doi: 10.1186/s13019-016-0518-8. PMID: 27487934; PMCID: PMC4973092.
120. Moritz AW, Wiedenhoefer JF, Profit AP, Jagirdar J. Breast adenomyoepithelioma and adenomyoepithelioma with carcinoma (malignant adenomyoepithelioma) with associated breast malignancies: A case series emphasizing histologic, radiologic, and clinical correlation. Breast. 2016 Oct;29:132-9. doi: 10.1016/j.breast.2016.07.018. Epub 2016 Aug 3. PMID: 27494340.
121. Xu J, Tang X, Iida Y, Fuchinoue F, Kusumi T, Yagihashi N, Kawachi K, Shimizu S, Masuda S. Adenomyoepithelioma with carcinoma of the breast: A report of two cases and a review of the literature. Pathol Res Pract. 2016 Feb;212(2):130-4. doi: 10.1016/j.prp.2015.09.008. Epub 2015 Sep 8. PMID: 26596263.
122. Smith Iorfido SM, Shah M, Iorfido SB, Yasir Zaidi S. Adenomyoepithelioma of the breast. Breast J. 2017 Nov;23(6):755-756. doi: 10.1111/tbj.12820. Epub 2017 Apr 10. PMID: 28397340.
123. Jones M, Fletcher J. Malignant adenomyoepithelioma of the breast. Pathology. 2017 Apr;49(3):322-325. doi: 10.1016/j.pathol.2016.11.015. Epub 2017 Feb 27. PMID: 28249686.
124. Logie N, Hugh J, Paulson K, Pearcey R, King KM. Radiotherapy in the Multidisciplinary Management of Adenomyoepithelioma of the Breast with an Axillary Lymph Node Metastasis: A Case Report and Review of the Literature. Cureus. 2017 Jun 21;9(6):e1380. doi: 10.7759/cureus.1380. PMID: 28775920; PMCID: PMC5522019.
125. Yuan Z, Qu X, Zhang ZT, Jiang WG. Lessons From Managing the Breast Malignant Adenomyoepithelioma and the Discussion on Treatment Strategy. World J Oncol. 2017 Aug;8(4):126-131. doi: 10.14740/wjon1055e. Epub 2017 Aug 27. PMID: 29147448; PMCID: PMC5650010.
126. Jassar A, Pathania K. Tubular variant of mammary adenomyoepithelioma: Diagnostic challenges and cytomorphological correlation in two cases. Cytojournal. 2017 Dec 27;14:29. doi: 10.4103/cytojournal.cytojournal_26_17. PMID: 29333189; PMCID: PMC5757281.
127. Baraban E, Zhang PJ, Jaffer S, Lubin D, Feldman M, Bleiweiss IJ, Nayak A. MYB rearrangement and immunohistochemical expression in adenomyoepithelioma of the breast: a comparison with adenoid cystic carcinoma. Histopathology. 2018 Dec;73(6):897-903. doi: 10.1111/his.13708. Epub 2018 Oct 22. PMID: 30003572.
128. Geyer FC, Li A, Papanastasiou AD, Smith A, Selenica P, Burke KA, Edelweiss M, Wen HC, Piscuoglio S, Schultheis AM, Martelotto LG, Pareja F, Kumar R, Brandes A, Fan D, Basili T, Da Cruz Paula A, Lozada JR, Blecua P, Muenst S, Jungbluth AA, Foschini MP, Wen HY, Brogi E, Palazzo J, Rubin BP, Ng CKY, Norton L, Varga Z, Ellis IO, Rakha EA, Chandarlapaty S, Weigelt B, Reis-Filho JS. Recurrent hotspot mutations in HRAS Q61 and PI3K-AKT pathway genes as drivers of breast adenomyoepitheliomas. Nat Commun. 2018 May 8;9(1):1816. doi: 10.1038/s41467-018-04128-5. PMID: 29739933; PMCID: PMC5940840.
129. Pareja F, Geyer FC, Brown DN, Sebastião APM, Gularte-Mérida R, Li A, Edelweiss M, Da Cruz Paula A, Selenica P, Wen HY, Jungbluth AA, Varga Z, Palazzo J, Rubin BP, Ellis IO, Brogi E, Rakha EA, Weigelt B, Reis-Filho JS. Assessment of *HMGA2* and *PLAG1* rearrangements in breast adenomyoepitheliomas. NPJ Breast Cancer. 2019 Jan 18;5:6. doi: 10.1038/s41523-018-0101-7. PMID: 30675516; PMCID: PMC6338730.
130. Pareja F, Toss MS, Geyer FC, da Silva EM, Vahdatinia M, Sebastiao APM, Selenica P, Szatrowski A, Edelweiss M, Wen HY, Mihai R, Varga Z, Foschini MP, Rubin BP, Ellis IO, Chandarlapaty S, Jungbluth AA, Brogi E, Weigelt B, Reis-Filho JS, Rakha EA. Immunohistochemical assessment of HRAS Q61R mutations in breast adenomyoepitheliomas. Histopathology. 2020 May;76(6):865-874. doi: 10.1111/his.14057. PMID: 31887226; PMCID: PMC7225035.
131. Díaz Del Arco C, Estrada Muñoz L, Pascual Martín A, Pelayo Alarcón A, de Pablo Velasco D, Ortega Medina L. Adenomioepitelioma de mama: descripción de cuatro casos y revisión de la literatura [Adenomyoepithelioma of the breast: Report of four cases and literature review]. Rev Esp Patol. 2018 Jan-Mar;51(1):55-60. Spanish. doi: 10.1016/j.patol.2016.12.001. Epub 2017 Jan 6. PMID: 29290325.
132. Antonelli MS, Mallel G, Pecoraro A, Vitale V, Maggi S, Lombardi A, Stanzani G, Amanti C. Adenomyoepithelioma of the breast: case report and literature review. G Chir. 2018 Jul-Aug;39(4):255-257. PMID: 30039795.
133. Ito R, Ota D, Ando S, Mori M, Fukuuchi A. A case of adenomyoepithelioma with myoepithelial carcinoma of the breast. Clin Case Rep. 2019 Mar 30;7(5):930-934. doi: 10.1002/ccr3.2100. PMID: 31110717; PMCID: PMC6509899.
134. Watanabe S, Otani T, Iwasa T, Takahama T, Takeda M, Sakai K, Nishio K, Ito A, Nakagawa K. A Case of Metastatic Malignant Breast Adenomyoepithelioma With a Codon-61 Mutation of HRAS. Clin Breast Cancer. 2019 Oct;19(5):e589-e592. doi: 10.1016/j.clbc.2019.05.001. Epub 2019 Jun 20. PMID: 31301988.
135. Kakkar A, Jangra K, Kumar N, Sharma MC, Mathur SR, Deo SS. Epithelial-myoepithelial carcinoma of the breast: A rare type of malignant adenomyoepithelioma. Breast J. 2019 Nov;25(6):1273-1275. doi: 10.1111/tbj.13463. Epub 2019 Jul 16. PMID: 31310415.
136. Kim MJ, Kim CS, Ju MJ, Park YS. Malignant adenomyoepithelioma of the breast: A rare case report. Int J Surg Case Rep. 2019;59:111-114. doi: 10.1016/j.ijscr.2019.04.045. Epub 2019 May 7. PMID: 31128547; PMCID: PMC6535691.
137. Hempenstall LE, Saxena M, Donaldson E. Malignant adenomyoepithelioma with multifocal adenosquamous carcinoma of the breast: A case report. Breast J. 2019 Jul;25(4):731-732. doi: 10.1111/tbj.13323. Epub 2019 May 6. PMID: 31062450.
138. Zhuo J, Li F, Wang ZD, Shi Y, Zhan SN, Du L. [Adenomyoepithelioma with carcinoma of the breast combined with adenoid cystic carcinoma: a case report]. Zhonghua Bing Li Xue Za Zhi. 2019 Jul 8;48(7):570-572. Chinese. doi: 10.3760/cma.j.issn.0529-5807.2019.07.017. PMID: 31288319.
139. Gafton B, Scripcariu V, Prutianu I, Alexa-Stratulat T, Terinte C, Nicolau A, Moisiuc D, Radu I. Challenges in management of male breast adenomioepithelioma with malignant behavior: Case report. Medicine (Baltimore). 2019 Oct;98(43):e17587. doi: 10.1097/MD.0000000000017587. PMID: 31651866; PMCID: PMC6824645.
140. Febres-Aldana CA, Mejia-Mejia O, Krishnamurthy K, Mesko T, Poppiti R. Malignant transformation in a Breast Adenomyoepithelioma Caused by Amplification of *c-MYC*: A Common pathway to Cancer in a Rare Entity. J Breast Cancer. 2019 Nov 8;23(1):93-99. doi: 10.4048/jbc.2020.23.e2. PMID: 32140273; PMCID: PMC7043941.
141. Ginter PS, McIntire PJ, Kurtis B, Mirabelli S, Motanagh S, Hoda S, Elemento O, Shin SJ, Mosquera JM. Adenomyoepithelial tumors of the breast: molecular underpinnings of a rare entity. Mod Pathol. 2020 Sep;33(9):1764-1772. doi: 10.1038/s41379-020-0552-x. Epub 2020 Apr 30. PMID: 32355271.
142. Wiens N, Hoffman DI, Huang CY, Nayak A, Tchou J. Clinical characteristics and outcomes of benign, atypical, and malignant breast adenomyoepithelioma: a single institution's experience. Am J Surg. 2020 Apr;219(4):651-654. doi: 10.1016/j.amjsurg.2019.03.026. Epub 2019 Apr 1. PMID: 30982573.
143. Lari EA, Lari AA, Alsaeed T. Malignant adenomyoepithelioma of the breast: A case report. Int J Surg Case Rep. 2020;72:56-58. doi: 10.1016/j.ijscr.2020.05.061. Epub 2020 May 30. PMID: 32506031; PMCID: PMC7283092.
144. Moro K, Sakata E, Nakahara A, Hashidate H, Gabriel E, Makino H. Malignant adenomyoepithelioma of the breast. Surg Case Rep. 2020 May 29;6(1):118. doi: 10.1186/s40792-020-00881-2. PMID: 32472226; PMCID: PMC7260303
145. Intagliata E, Gangi S, Trovato C, Vecchio R, Strazzanti A. Benign adenomyoepitelioma of the breast: Presentation of two rare cases and review of literature. Int J Surg Case Rep. 2020;67:1-4. doi: 10.1016/j.ijscr.2020.01.010. Epub 2020 Jan 23. PMID: 31991375; PMCID: PMC6992528.
146. Harada J, Horiguchi SI, Ishiba T, Kubota H, Ichida M, Sakurai N, Arai H, Motoi T, Aruga T, Hishima T. A Case of Adenomyoepithelioma With a Pleomorphic Adenoma-Like Component of the Male Breast. Int J Surg Pathol. 2020 Dec 8:1066896920978824. doi: 10.1177/1066896920978824. Epub ahead of print. PMID: 33289419.
147. Amano Y, Sakaguchi-Tamba M, Sasaki Y, Oshiro H, Fukushima N, Fujita T, Masuda S, Niki T. Adenomyoepithelioma with a human epidermal growth factor receptor 2-fluorescence in situ hybridization-confirmed ductal carcinoma in situ component: A case report and review of the literature. Medicine (Baltimore). 2020 Oct 16;99(42):e22665. doi: 10.1097/MD.0000000000022665. PMID: 33080708; PMCID: PMC7572011.
148. El-Helou E, Terro JJ, Kansoun A, Neaime GR, Mochairefa H, Ismail N, Naccour J, Zaarour M, Alam H. Breast adenomyoepithelioma, a case report. Int J Surg Case Rep. 2020;77:660-663. doi: 10.1016/j.ijscr.2020.11.110. Epub 2020 Nov 21. PMID: 33395868; PMCID: PMC7708873.
149. Zhang Z, Wang Y, Xie X, Peng J, Hong J, Bi L, Yang M. Malignant adenomyoepithelioma of the breast: A case report. Medicine (Baltimore). 2021 Feb 5;100(5):e24461. doi: 10.1097/MD.0000000000024461. PMID: 33592899; PMCID: PMC7870234.
